# High-quality chromosome-level genome assembly and full-length transcriptome analysis of the pharaoh ant *Monomorium pharaonis*

**DOI:** 10.1093/gigascience/giaa143

**Published:** 2020-12-15

**Authors:** Qionghua Gao, Zijun Xiong, Rasmus Stenbak Larsen, Long Zhou, Jie Zhao, Guo Ding, Ruoping Zhao, Chengyuan Liu, Hao Ran, Guojie Zhang

**Affiliations:** State Key Laboratory of Genetic Resources and Evolution, Kunming Institute of Zoology, Chinese Academy of Sciences, Kunming, Yunnan 650223, China; State Key Laboratory of Genetic Resources and Evolution, Kunming Institute of Zoology, Chinese Academy of Sciences, Kunming, Yunnan 650223, China; BGI Education Center, University of Chinese Academy of Sciences, Shenzhen 518083, China; BGI-Shenzhen, Beishan Industrial Zone, Shenzhen 518083, China; Villum Center for Biodiversity Genomics, Section for Ecology and Evolution, Department of Biology, University of Copenhagen, Copenhagen DK-2100, Denmark; BGI-Shenzhen, Beishan Industrial Zone, Shenzhen 518083, China; State Key Laboratory of Genetic Resources and Evolution, Kunming Institute of Zoology, Chinese Academy of Sciences, Kunming, Yunnan 650223, China; State Key Laboratory of Genetic Resources and Evolution, Kunming Institute of Zoology, Chinese Academy of Sciences, Kunming, Yunnan 650223, China; BGI-Shenzhen, Beishan Industrial Zone, Shenzhen 518083, China; Villum Center for Biodiversity Genomics, Section for Ecology and Evolution, Department of Biology, University of Copenhagen, Copenhagen DK-2100, Denmark; State Key Laboratory of Genetic Resources and Evolution, Kunming Institute of Zoology, Chinese Academy of Sciences, Kunming, Yunnan 650223, China; State Key Laboratory of Genetic Resources and Evolution, Kunming Institute of Zoology, Chinese Academy of Sciences, Kunming, Yunnan 650223, China; State Key Laboratory of Genetic Resources and Evolution, Kunming Institute of Zoology, Chinese Academy of Sciences, Kunming, Yunnan 650223, China; State Key Laboratory of Genetic Resources and Evolution, Kunming Institute of Zoology, Chinese Academy of Sciences, Kunming, Yunnan 650223, China; BGI-Shenzhen, Beishan Industrial Zone, Shenzhen 518083, China; Villum Center for Biodiversity Genomics, Section for Ecology and Evolution, Department of Biology, University of Copenhagen, Copenhagen DK-2100, Denmark; Center for Excellence in Animal Evolution and Genetics, Chinese Academy of Sciences, 32 Jiaochang Donglu, Kunming 650223, China

**Keywords:** social insects, *Monomorium pharaonis*, long-read sequencing, alternative splicing, long non-coding RNA

## Abstract

**Background:**

Ants with complex societies have fascinated scientists for centuries. Comparative genomic and transcriptomic analyses across ant species and castes have revealed important insights into the molecular mechanisms underlying ant caste differentiation. However, most current ant genomes and transcriptomes are highly fragmented and incomplete, which hinders our understanding of the molecular basis for complex ant societies.

**Findings:**

By hybridizing Illumina, Pacific Biosciences, and Hi-C sequencing technologies, we *de novo* assembled a chromosome-level genome for *Monomorium pharaonis*, with a scaffold N50 of 27.2 Mb. Our new assembly provides better resolution for the discovery of genome rearrangement events at the chromosome level. Analysis of full-length isoform sequencing (ISO-seq) suggested that ∼15 Gb of ISO-seq data were sufficient to cover most expressed genes, but the number of transcript isoforms steadily increased with sequencing data coverage. Our high-depth ISO-seq data greatly improved the quality of gene annotation and enabled the accurate detection of alternative splicing isoforms in different castes of *M. pharaonis*. Comparative transcriptome analysis across castes based on the ISO-seq data revealed an unprecedented number of transcript isoforms, including many caste-specific isoforms. We also identified a number of conserved long non-coding RNAs that evolved specifically in ant lineages and several that were conserved across insect lineages.

**Conclusions:**

We produced a high-quality chromosome-level genome for *M. pharaonis*, which significantly improved previous short-read assemblies. Together with full-length transcriptomes for all castes, we generated a highly accurate annotation for this ant species. These long-read sequencing results provide a useful resource for future functional studies on the genetic mechanisms underlying the evolution of social behaviors and organization in ants.

## Background

Ants are an ecologically diverse and extraordinarily successful animal group, which occupy almost all terrestrial ecological niches [1]. As social insects, ants live in colonies composed of up to millions of individuals, which develop into different social castes with remarkable division of labor and substantial variations in morphology, physiology, and behavior [2]. The sexual castes, including reproductively active queens, gynes (virgin queens), and males, are specialized for sexual reproduction, whereas the worker caste, which can be divided into distinct sub-castes in some species, are specialized for non-reproductive support roles, such as constructing, maintaining, and defending the nest, collecting food, and rearing the brood [3].

Understanding the genetic mechanisms underlying caste development and differentiation processes has been the major focus of recent studies on social insects. Such research has indicated that caste differentiation involves the regulation of both genetic and epigenetic factors [4]. Comparative genome and transcriptome studies have identified several key genes that show differential expression patterns among castes and may contribute to caste-specific phenotypes, e.g., *vitellogenin, foraging, arrestin*, and *insulin/insulin-like growth factor signaling* [1, 5–12]. Recent studies also suggest that alternative splicing Alternative splicing (AS), which can increase genetic regulatory complexity, may contribute to phenotypic plasticity in eusocial insects [11, 13–16]. Additionally, epigenetic mechanisms, such as long non-coding RNAs (lncRNAs), may also participate in gene expression regulation during caste differentiation [17, 18]. Particularly, comparative genomic studies cross multiple ant lineages have identified many conserved lncRNAs that may play potential roles in the evolution of the caste system in ants [19, 20].

However, most previous genome studies have relied on short-read sequencing technology [1, 21]. This has resulted in fragmented assemblies with many sequencing gaps, which is primarily due to high GC content or repeat regions failing to sequence. Additionally, short-read–based RNA-seq also often fails to resolve complex Alternative splicing (AS) isoforms, which are ubiquitously present in eukaryotes [22]. Single-molecule real-time (SMRT) long-read sequencing overcomes these limitations by generating ultra-long reads and offering different solutions to solve genome assembly problems, including complex regions with repeated elements or segmental duplications or regions with high GC content [23]. Long-read sequencing is also beneficial in transcriptomics by providing full-length reads that span the entire transcript isoform, thereby eliminating the need for transcript reconstruction and inference. Thus, full-length isoform sequencing (ISO-seq) can substantially improve annotations of reference genomes, characterize isoforms in important genes, capture alternative splice variants, and identify lncRNAs. Currently, only 27 ant genomes have been published, most of which are limited in their quality [21]. Therefore, high-quality genomes and full-length transcriptomes of ant species are needed to understand the molecular mechanisms involved in caste differentiation and the reproductive division of labor.

**Figure 1:**
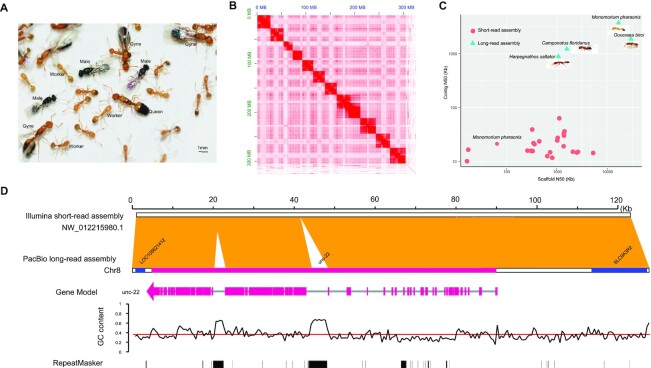
Characterization of *M. pharaonis* genome assembly. **(A)** Photo of pharaoh ant (*Monomorium pharaonis*) colony with 4 ant castes (queens, gynes, males, and workers). **(B)** Heat map of Hi-C interactions among all chromosomes of pharaoh ant. **(C)** Comparison of scaffold N50s and contig N50s of 27 short-read–assembled (pink filled circles) and 4 long-read–assembled (blue filled triangles) ant genomes. Previous short-read assembly for *M. pharaonis* is marked on the plot. **(D)** Genome collinearity of short-read and PacBio long-read assemblies shows that PacBio assembly exhibits better coverage of high GC content regions and repeat sequences. Blue indicates genes assembled by both sequencing methods; red indicates genes that are incomplete in short-read assembly but complete in PacBio assembly.

The pharaoh ant *Monomorium pharaonis* (NCBI: txid307658; Fig. 1A) is an emerging model animal for genomic and molecular studies of caste differentiation in social insects. Unlike most ant species, the pharaoh ant has a very short life span, is easy to rear, and can mate and reproduce within the colony, which makes them a perfect model organism for genetic studies. The first draft pharaoh ant genome was assembled on the basis of short reads [24], resulting in a very fragmented assembly with a scaffold N50 length of only 75.38 kb.

In this study, using Pacific Biosciences (PacBio) SMRT DNA Sequencing and ISO-seq technology combined with Illumina short reads and Hi-C (high-throughput chromosome conformation capture) data, we produced a high-quality chromosome-level reference genome and high-quality transcriptome for the pharaoh ant. Using these data, we further analyzed the protein-coding genes, AS isoforms, and lncRNAs. This study should help enhance our understanding of the genetic and epigenetic mechanisms of complex ant societies.

## Analyses

### Genome assembly, assessment, and gene prediction

Following routine 17-mer analysis [25] with short-read sequencing, the genome of *M. pharaonis* was estimated to be 342 Mb (Supplementary Fig. S1, Table S1). Using other *k*-mer sizes produced similar estimations. We generated 33 Gb (∼103×) of Illumina short-read sequencing data and >31 Gb (∼96×) of PacBio sequencing data, resulting in 4,151,307 total reads (Supplementary Table S2). Genome of *M. pharaonis* was assembled into contigs by Canu using the PacBio Sequel sequencing data [26] and was scaffolded using the SSPACE_longRead scaffolder [27]. The assembled scaffolds were gap-filled using the PBJelly program [28], and polished with the PacBio data and short sequencing reads using Quiver and Pilon, respectively (Supplementary Table S3, see Methods for details). By BLAST searching against the Bacteria and Virus databases, we identified 151,589 bp of contaminated sequences, mainly from insect endosymbionts, such as *Wolbachia, Bacillus, Acinetobacter*, and *Candidatus*. Duplicated haplotigs and artifacts were identified and removed using the purge_haplotigs pipeline [29]. After removal of the contaminated sequences, duplicated haplotigs, and artifacts, the final assembly from the PacBio reads was 313 Mb with a scaffold N50 length of 3.85 Mb (193 scaffolds) and contig N50 length of 2.77 Mb (301 contigs) (Table 1). The Phred quality value (QV) of the whole genome was calculated as QV = 50 (99.999% accuracy), which suggests that the assembly was of high quality [30, 31].

**Table 1:** Summary of *M. pharaonis* genome features

Reads	PacBio assembly	Hi-C assembly
Genome assembly size (bp)	312,903,204	313,026,204
No. of scaffolds	193	274
Scaffold N50 (bp)	3,854,274	27,237,342
Scaffold N90 (bp)	800,084	20,211,500
Maximum scaffold length (bp)	18,497,097	48,563,521
No. of contigs	301	628
Contig N50 (bp)	2,769,621	2,456,926
Contig N90 (bp)	573,845	430,526
Maximum contig length (bp)	9,733,832	9,249,838
GC content (%)	36.39	36.39
BUSCO assessment (n = 4 ,415)	C: 98.4%, D: 2.1%, F: 1.1%	

C: complete BUSCOs; D: duplicated BUSCOs, F: fragmented BUSCOs.

Hi-C uses high-throughput sequencing to map genome-wide chromatin contacts and has been widely used as a scaffolding method in genome assembly [32]. We generated 14.82 Gb of Hi-C sequencing data and mapped them to the polished pharaoh ant genome using Juicer software [33] after filtering low-quality data with Hic-Pro [34] to improve the connection integrity of the contigs. The locations and directions of contigs were determined by 3D *de novo* assembly (3d-DNA) software [35] with default parameters, after which the contigs were successfully clustered and anchored to 11 linkage groups (Fig. 1B, Supplementary Table S4), which covered 94% of the pharaoh ant assembled sequences. Last, we obtained a high-quality chromosome-level pharaoh ant genome with a contig N50 length of 2.5 Mb and scaffold N50 length of 27.2 Mb (Table 1). This final assembly produced a shorter N50 than before Hi-C linkage because some artificial links introduced by SSPACE were further removed during the Hi-C assembly process if the links were not supported by Hi-C data or violated Hi-C links.

Compared with the other 27 published ant genomes, which were mostly sequenced and assembled using short-read sequencing, the pharaoh ant genome assembly showed a significantly higher contiguity level (Fig. 1C, Supplementary Table S5). Our genome assembly with PacBio reads was also more complete than other published ant genomes, with gaps only accounting for 0.0867% of the new assembly compared to an average of 3.75% for other ant genomes with short-read assembly. Specifically, we compared genomic regions and found that a large number of regions with high GC content were missed in previous short-read assemblies of the pharaoh ant genome [24] but are covered in the new assembly (Fig. 1D). Specifically, 9.76% of genes with >70% GC content (4 of 41) and 11.30% (52 of 460) of genes with 60%–70% GC content were missing in previous short-read assemblies but were recovered in our assembly, thereby indicating that the PacBio-assembled genome had significant advantages for high GC content genes (Supplementary Table S6). Furthermore, the completeness of our PacBio assembly was assessed by BUSCO, which indicated that 98.4% of the 4,415 expected Hymenoptera conserved genes were identified as complete (Table 1).

Gene prediction was first performed by combining homology–, *de novo–*, and transcriptome-RNA-seq–based searching and identification methods. The ISO-seq data were then used to further improve the gene models predicted in the previous steps (see Methods for details), including the annotation of untranslated regions (UTRs), introduction of new coding exons, modification of incorrect gene models, and rediscovery of missing genes. Finally, a total of 15,327 non-redundant protein-coding genes were predicted in the pharaoh ant genome assembly. By searching against functional databases (i.e., TrEMBL, COG, SwissProt, GO, and KEGG) and annotating with InterProScan, we annotated 15,242 (99.45%) genes and identified 13,831 (90.24%) genes with conserved motifs (Table 2).

**Table 2:** Statistics of functional annotation of protein-coding genes in pharaoh ant

Statistic	Number	Percent (%)
Total	15,327	
InterPro	13,831	90.24
COG	4,739	30.92
GO	8,562	55.86
KEGG	12,817	83.62
SwissProt	10,659	69.54
TrEMBL	15,229	99.36
Annotated	15,242	99.45
Unannotated	85	0.55

### High-frequency chromosome recombination in ant genome

**Figure 2:**
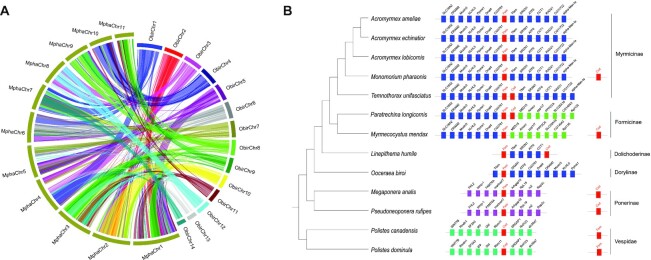
Genome collinearity and gene synteny of *M. pharaonis* (Mpha). **(A)** Genome collinearity of chromosome-level–assembled pharaoh ant and clonal raider ant (*Ooceraea biroi* [Obir]), showing marked genome rearrangements during genome evolution of the 2 species. **(B)** Synteny of flanking region of *fem* and *csd* across 11 ant species using recently produced reference genomes from the Global Ant Genomics Alliance (GAGA) and across 2 wasp species downloaded from the NCBI. Showing the rearrangements of *fem* and *csd* during ant genome evolution.*csd* and *fem* are marked in red, and other colors represent their neighbor genes in PacBio-assembled ant and ancestor wasp species.

Chromosome-level assembly can provide improved resolution to construct ancestral karyotypes and detect genome rearrangement events during speciation [36]. To demonstrate the advance in chromosome-level assemblies, we performed genome collinearity analyses between the chromosome-level–assembled pharaoh ant genome (2*n* = 22) [37, 38] and the clonal raider ant (*Ooceraea biroi*) genome (2*n* = 28) [39]. The synteny map spanned 14 *O. biroi* chromosomes and 11 *M. pharaonis* chromosomes, covering 94% of the *M. pharaonis* genome (Fig. 2A). The longest syntenic block spanned 530 genes in the pharaoh ant. On average, only 3.17 genes were maintained in the same syntenic block between the 2 species, implying a high frequency of rearrangement in the 2 genomes. Furthermore, we detected ∼150 fissions/fusions at the interchromosomal level with >500 kb block resolution between the 2 species. This represents 2.04 chromosome breakpoints per Mb per MY, a faster rate than that reported for some insect groups such as the *Drosophila* genus [40]. To detail the micro-synteny evolutionary pattern across ant lineages, we investigated the orthologs of genes upstream and downstream of *feminizer*(*fem*)*and complementary sex determiner*(*csd*) across 11 ant species using their recent PacBio genome assemblies from the Global Ant Genomics Alliance (GAGA) and across 2 wasp species downloaded from the NCBI. *Csd* is the primary sex-determining signal in most eusocial Hymenoptera and arose from the duplication of the *fem* gene, which plays a key role in sex determination [41, 42]. On the basis of synteny analysis of *fem* and *csd* and neighbor genes, *fem* was present in all investigated species; however, its synteny with neighbor genes underwent several translocation and recombination events during the diversification of ant lineages (Fig. 2B). In contrast, not all ant species possessed the *csd* homolog and genomic locations differed among ant species. These results thus indicate that *csd* and *fem* may function differently in each lineage.

### ISO-seq significantly improves gene annotation

Transcriptome data allow us to identify all expressed genes and provide important evidence for gene annotation. Currently, most published genomes have been annotated using RNA-seq data by either mapping short reads or pre-assembled transcripts with short reads onto reference genomes [43]. Single-molecule long-read sequencing produces a full-length transcript of up to 10 kb, which can be readily used for gene prediction without the need of assembly. In principle, therefore, this can significantly improve gene annotation. To provide insight into how gene annotation can be improved with long-read ISO-seq, we sequenced total RNA from the whole bodies of *M. pharaonis* workers, gynes, queens, and males using 2 sequencing platforms, i.e., PacBio SMRT for long reads and BGI-seq for short reads. We compared the performance of these 2 datasets in gene prediction and isoform annotation. In total, we obtained 62 Gb of long-read transcriptome data (Supplementary Table S7) and 236 Gb of RNA-seq data (Supplementary Table S8).

**Figure 3:**
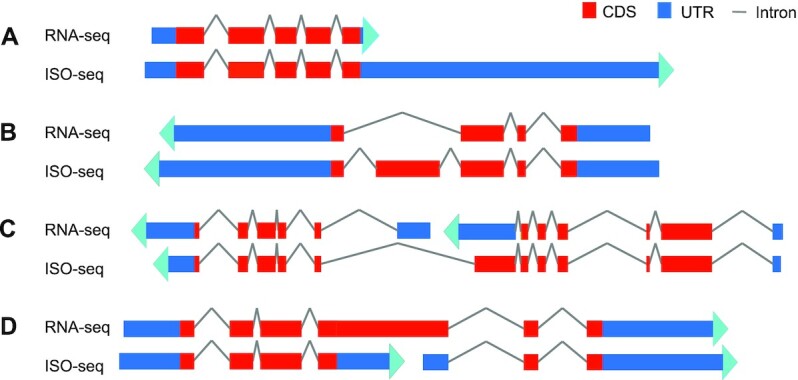
Comparison of RNA-seq and ISO-seq gene annotations. **(A)** UTRs newly annotated in ISO-seq annotation. **(B)** Genes annotated incompletely by missing exons in RNA-seq annotation. **(C)** One gene was misannotated to multiple genes in RNA-seq annotation. **(D)** Two genes were misannotated as a combined gene in RNA-seq version but were correctly annotated in ISO-seq data. CDS, coding sequences; UTR, untranslated region.

We then generated 2 annotations for *M. pharaonis* using the ISO-seq and RNA-seq data separately and compared gene model predictions, AS events, UTR annotations, and predicted gene completeness (Supplementary Table S9). The ISO-seq annotation identified 186,499 transcripts on 10,626 protein-coding gene loci, with an average of 5.37 exons per transcript. On the basis of the analysis, the ISO-seq annotation improved upon the RNA-seq annotation in several ways. First, the UTRs of 10,004 genes annotated in the ISO-seq version were missed in the RNA-seq annotation (Fig. 3A). Second, RNA-seq annotation missed ≥1 exon in 2,093 genes, which were identified in the ISO-seq annotation (Fig. 3B). Third, the ISO-seq annotation also corrected the models of 58 genes falsely annotated into multiple genes (Fig. 3C) and 99 genes mistakenly merged with neighbor genes (Fig. 3D) in the RNA-seq annotation. Although high-depth RNA-seq data should, in principle, provide single-base resolution for transcriptome profiling, we found 279 genes in the ISO-seq annotation that were missing in the RNA-seq annotation. Among them, >18% were high GC content genes and 38% had >200 bp repeat sequences, further demonstrating that PacBio is better for sequencing high GC content genes and repeat sequences. In total, the coding area of 15.86% of genes was refined with the ISO-seq data, thus highlighting the power of long-read sequencing in gene annotation. Altogether, we annotated 15,327 genes in the pharaoh ant after merging the ISO-seq and RNA-seq annotations.

### AS landscape of *M. pharaonis*

To identify the AS transcripts, we first clustered all high-quality long reads into final polished isoforms. More than 97.95% of the consensus transcripts were mapped to the reference genome using GMAP [44] (Supplementary Table S10), again indicating the high completeness of the reference genome. We next collapsed the redundant isoforms into 186,499 isoforms, covering 11,499 genes expressed in ≥1 caste. Splice junctions (SJs) were detected according to the 2 pairs of dinucleotides presented at the beginning and end of the introns encompassed by the junctions. The SJs were dominated by the canonical GT-AG form, which accounted for >94.72% of total SJs. More than 99% of the SJs with the GT-AG form identified from ISO-seq were also supported by the RNA-seq data (Supplementary Table S11). These findings suggest high accuracy of the exon-intron boundary structure based on long reads and strongly support the validity of the alternative-spliced isoform detection.

**Figure 4:**
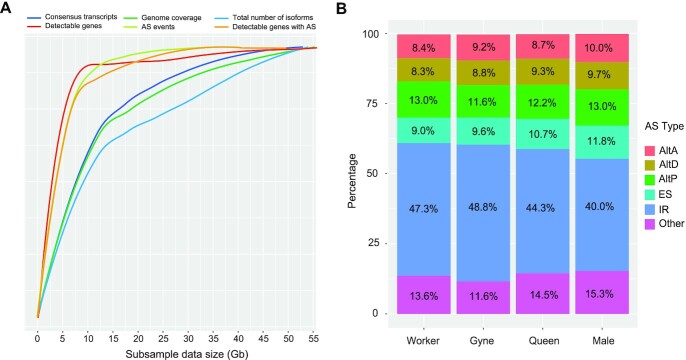
Characterization of *M. pharaonis* isoforms from PacBio ISO-seq in 4 castes. **(A)** Saturation analysis of PacBio ISO-seq data on consensus transcripts, genome coverage, total number of isoforms, detectable genes, alternative splicing (AS) events, and detectable genes with AS. Consensus transcripts were yielded from multiple full-length non-chimeric reads in a single zero-mode waveguide by transcript clustering analysis. Because many isoforms could not be mapped to the reference genome owing to either sequencing errors or artificial transcripts, the total number of isoforms, which represents isoforms finally confirmed by mapping to the reference genome, was lower than the consensus transcripts.**(B)** Distribution of AS events in 4 ant castes. AltA, alternative acceptor site; AltD, alternative donor site; AltP, alternative position; ES, exon skipping; IR, intron retention.

A practical question in transcriptome sequencing is at what sequencing depth the data can provide sufficient signals for AS event detection and comparison. The high-coverage ISO-seq data generated here allowed us to address this question by performing saturation analyses with subtractive samples. We evaluated the effects of the amount of sequencing data on the number of consensus transcripts, genome coverage, total number of isoforms, detectable genes, AS events, and detectable genes with AS (Fig. 4A). By mapping the high-depth RNA-seq reads (236 Gb) onto the *M. pharaonis* genome, we estimated that 140.22 Mb of genomic regions could be transcribed in ≥1 caste. Furthermore, from the 59 Gb of raw ISO-seq long-read transcripts produced for all samples, we detected 129 Mb of expressed regions that covered 92% of potential transcribed regions detected by RNA-seq. Indeed, we found that the number of consensus transcripts, size of expressed regions, and total number of isoforms increased with the amount of ISO-seq data and only reached saturation at 50 Gb. This indicates an overabundance of transcripts in the *M. pharaonis* transcriptome and suggests that some low-abundance or rare transcript isoforms remain to be discovered with more sequencing data. An alternative explanation is that ISO-seq may produce artificial isoforms, and thus more novel isoforms could appear with the increase in sequencing data. To confirm this, we used RNA-seq data to validate the unique AS events for each isoform and found that ∼2% of isoforms detected in ISO-seq were not supported by RNA-seq.

Nevertheless, we found that the numbers of expressed genes and genes with AS events, as well as the total number of AS events, had already reached their saturation at ∼10 Gb of ISO-seq data. With this amount of data, we detected 9,656 expressed genes, covering ≥93.54% of genes from RNA-seq transcription evidence (see example shown in Supplementary Fig. S2). These results suggest that the sequencing data obtained for each caste (∼23.0, 14.0, 15.3, and 10.4 Gb for workers, gynes, queens, and males, respectively) were sufficient for covering most expressed genes and AS events.

To obtain the overall AS pattern in *M. pharaonis*, all ISO-seq data were pooled for AS event and gene isoform detection. Results showed that >87% of expressed genes had ≥2 isoforms and, on average, each gene expressed 9 isoforms in all castes, indicating the complex nature of the ant transcriptome. Similar to that reported in humans and many other eukaryotic species [45], intron retention was the most dominant AS form, accounting for 48.77% of all AS events in the pharaoh ant. This ratio was also observed across caste samples (Fig. 4B, Supplementary Table S12). Of note, 654 genes had >50 isoforms. The most extreme case was the mitochondrial NADH-ubiquinone oxidoreductase gene, which was transcribed into 894 isoforms. These isoform-rich genes were enriched in many biological processes involved in cell signal transduction, including signal transduction (GO:0007165), cell communication (GO:0007154), signaling (GO:0023052), cation channel activity (GO:0005261), ion channel activity (GO:0005216), and potassium channel activity (GO:0005267) (Supplementary Table S13). The increasing transcription abundance of these genes through AS might enhance cellular responses to environmental stimuli.

### Characterization of caste-specific AS isoforms

AS is an important mechanism in defining tissue specificity based on tissue-specific expression of transcripts of the same gene. Previous studies have shown that AS is associated with phenotypic variation in eusocial insects, where a single genome is able to encode for numerous caste phenotypes [11, 13, 15, 16, 46]. Thus, we investigated isoform specificity and commonality among the 4 castes. Because of the high cost of ISO-seq, we used pooled samples for each caste instead of biological replications to mitigate the variation across individuals/colonies to produce high-coverage sequencing data that ensures that we discovered isoforms with low expression levels. Owing to the lack of biological replications, our results might only be representative for the colony. However, the AS isoforms produced from this in-depth investigation can also be valuable as a reference for further studies. Among all expressed genes, 5,359 transcribed ≥1 caste-specific isoform that was only presented in 1 caste. These results suggest that AS has had pervasive effects on genome-wide protein-coding genes with diverse functions that may contribute to caste differentiation. Following KEGG analysis, we identified many genes with caste-specific isoforms related to the insulin and mTOR signaling pathways, which play key roles in regulating caste differentiation on morphology and longevity [12, 47, 48] (Supplementary Tables S14–17).

To further characterize the caste-specific AS isoforms, we highlighted some functionally important genes that may play important roles in ant sex determination and caste differentiation. *Feminizer* (*fem*) functions as a binary switch gene participating in sex determination and sexual differentiation in Hymenoptera [41, 42, 49]. In the pharaoh ant, *fem* consisted of 8 coding exons. However, full-length transcripts with all coding exons were only expressed in the female castes (Supplementary Fig. S3), with the male caste just expressing the first 2 coding exons. These results suggest that they have different functions according to their differences in protein domains. The sex-based differences in the AS pattern of *fem* seem to be conserved, similar to the *transformer* gene, across different insects [49]. Moreover, we found that the female castes expressed diverse transcript isoforms of this gene, with many isoforms possibly functioning as lncRNAs.

By choosing the highest-expressed isoform for each caste, we screened out the genes with dominant AS isoforms for each caste and selected 267 genes with caste-specific dominant AS isoforms (Supplementary Table S18). We reasoned that these genes might be potential candidates involved in ant caste differentiation via AS. On the basis of GO term analysis, we revealed that these genes were significantly enriched in phosphotransferase activity (GO:0016773), carbohydrate derivative binding (GO:0097367), and neurotransmitters (GO:0005328) (Supplementary Table S19). For example, *cytokine receptor-like factor 3* (*crlf3*), which is a neuroprotective erythropoietin receptor in beetle (*Tribolium castaneum*) and locust (*Locusta migratoria*) neurons and emerged with the evolution of the eumetazoan nervous system [50, 51], had several caste-specific isoforms and different dominant expressed isoforms in each pharaoh ant caste (Supplementary Fig. S4). The worker-specific isoform of this gene showed the highest expression level in workers. Queens also mainly expressed their caste-specific isoform. Considering the key role of the nervous system in caste differentiation, these results further indicate that the caste-specific dominant AS isoform of *crlf3* may influence caste differentiation in pharaoh ants.

### Identification and comparative analysis of lncRNAs

**Figure 5:**
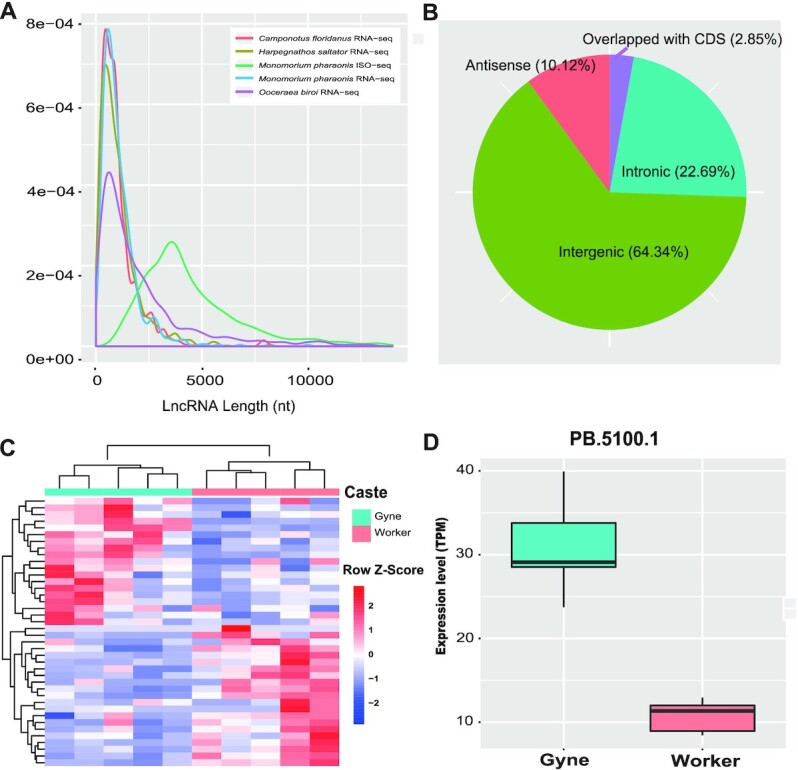
Characterization of lncRNAs. **(A)** Comparisons of lncRNA length distribution among 4 species and 2 sequencing methods. **(B)** Classification of lncRNAs in pharaoh ant. **(C)** Heat map shows differentially expressed lncRNAs between worker and gyne brains. Each row represents 1 lncRNA, and each column represents 1 replicate of the corresponding caste. Relative lncRNA expression is depicted according to color scale. **(D)** Example of a highly conserved differentially expressed lncRNA between worker and gyne brains. The black line in the box indicates the median. TPM: transcripts per million.

LncRNAs are a group of RNA molecules (>200 nt) that are not translated into proteins but that play important roles in a variety of biological processes [52]. The detection of lncRNAs has been restricted by short-read RNA sequencing technology because short-read sequencing fails to capture the full length of extremely long lncRNAs. Therefore, the number of previously detected lncRNAs is likely to be underestimated and should be improved by ISO-seq. Here, we detected 1,225 long transcripts that likely function as lncRNAs on the basis of their lack of open reading frames (see Methods). The lengths of these lncRNAs varied from 923 to 30,849 bp, which are far longer than those of lncRNAs predicted in other ant species, such as *Camponotus floridanus, Harpegnathos saltator*, and *O. biroi*, using RNA-seq (Fig. 5A) [20, 53]. Using their relative positions to the annotated genome, pharaoh ant lncRNAs could be classified into 4 categories: i.e., antisense, overlapping with coding sequences, intronic, and intergenic (Fig. 5B) [54]. Most lncRNAs were located in the intergenic region (64.33%), as observed in other organisms, and probably function as transcription regulators [55, 56].

The number of lncRNAs in *M. pharaonis* varied among castes. Gynes had the highest number of lncRNAs, whereas males had the lowest number (Table 3). Queens had the longest lncRNAs among the 4 castes (Wilcoxon test, *P* < 0.01), with an average length of 5,675 bp, whereas males had the shortest lncRNAs among the 4 castes (Wilcoxon test, *P* < 0.01), with an average of 3,456 bp (Table 3).

**Table 3:** Statistics of predicted lncRNAs in 4 castes

		Length (bp)		
Sample	No. lncRNAs	Minimum	Maximum	Average
Worker	531	942	25,018	4,438
Gyne	543	982	19,746	4,648
Queen	360	1,344	30,849	5,675
Male	149	923	12,182	3,456

Although investigating how lncRNAs work is challenging because of their relatively weak expression, cell/tissue-specificity, and variable functions, some lncRNAs exhibit high conservation in either sequence or secondary structure across species, thus providing a way to detect evolutionary signals for functional importance. By genomic comparison of the 4 ant genomes sequenced with long reads, we identified genomic regions showing extremely low mutation rates with high conservation across all detected species. We found 961 (78%) lncRNAs that contained ≥1 highly conserved genomic element across all ant species, which likely experienced strong purifying selection during ant evolution. Based on orthologous analysis of lncRNAs between ants and parasitoid wasps (*Nasonia vitripennis*), ants and bees (*Apis mellifera*), and ants and flies (*Drosophila melanogaster*), we obtained a set of insect-conserved lncRNAs. Among the ant-conserved lncRNAs, 33 were conserved between ants and bees, 12 were conserved between ants and parasitoid wasps, and 6 were conserved between ants and flies, thus demonstrating that most of these lncRNAs were ant-specific.

We further identified conserved lncRNAs showing differential expression between worker and gyne by using the brain RNA-seq data produced by Qiu et al. [57]. We detected the differently expressed lncRNAs using DESeq2 and then classified lncRNA transcripts as differentially expressed between castes when the false discovery rate (FDR)-adjusted *P*-value was ≤0.05. By doing so, we detected 32 ant conserved lncRNAs that were differentially expressed between worker and gyne in *M. pharaonis* (Fig. 5C and D). For example, the single-exon lncRNA, PB.5100.1, which is located between *ACHE1* (*Acetylcholinesterase 1*) and *RCBTB1* (*RCC1 and BTB domain-containing protein 1*), was highly expressed in gyne samples (Fig. 5D).

## Conclusions

Our study provided a high-quality chromosome-level genome assembly and full-length transcriptomes for all 4 castes of the pharaoh ant. Our newly assembly genome showed markedly improved quality compared with previous short-read sequencing assemblies [24]. Our comparison demonstrated the importance of using long-read sequencing to cover genomic assembly of both repeat and high GC content regions, particularly for the latter, which often span genomic elements with regulatory functions (e.g., promoters). By combining PacBio assembly and Hi-C data, our study presented an efficient way in which to produce a chromosome-level assembly for the ant genome. This has now been adapted as a standard genomic sequencing and assembly pipeline for the GAGA, which aims to generate high-quality assemblies for ∼200 ant species representing broad diversity [21]. Furthermore, our ISO-seq not only produced a high-quality genome annotation for *M. pharaonis* but also highlighted the complexity and diversity of the ant transcriptome, which may be associated with caste differentiation. Our study also identified many protein-coding genes with caste-specific isoforms and a core set of lncRNAs that may play conserved roles in ant caste differentiation over the long evolutionary process of ants. These datasets will be valuable for downstream functional studies to reveal the genetic mechanisms underlying caste differentiation in ants.

## Methods

### Sample collection

*M. pharaonis* were collected from a house in Mengla, Xishuangbanna district, Yunnan Province, China. The colony was brought back to the laboratory and reared under constant conditions, i.e., temperature of 27°C, relative humidity (RH) of 65%, and light: dark cycle of 12 h:12 h (light period 08:00–20:00, dark period 20:00–08:00). The queens and workers used in this study were from the starting colony (MP-MQ-018). Gyne and male samples were obtained from a newly developed colony, which was isolated from the starting colony with only eggs, larvae, and workers. For DNA and RNA sequencing, ants were collected and flash-frozen in liquid nitrogen and stored at −80°C for later extraction. The collection procedures were in accordance with protocols approved by the Animal Care and Use Committee of the Kunming Institute of Zoology, China.

### DNA and RNA extraction

Because of their small size, genomic DNA from pools of worker samples was extracted via an insect sodium dodecyl sulfate DNA extraction protocol provided by the Novogene Corporation (Nanjing, China). Total RNA was extracted from the pooled individuals of each caste (male, worker, gyne, and queen) for PacBio full-length isoform sequencing (ISO-seq) using a Trizol Extraction Kit according to the manufacturer's instructions. All samples were collected from sub-colonies developed from the same starting colony to reduce biological variation of the data. There were 3 major purposes for performing ISO-seq analyses in the present study: (i) to assist in annotation, (ii) to identify AS forms, and (iii) to identify lncRNAs. Because these analyses rely on the full coverage of expressed transcripts, especially those with low levels of expression, the sequencing depth of the ISO-seq data was more important than biological replications. Quantification analyses were performed using the RNA-seq data (with each having 5 biological replicates) produced in our previous study on the same ant castes [57]. Male brains were dissected in cold diethyl pyrocarbonate (DEPC)-treated phosphate-buffered saline (PBS). Five replicates of pooled male brains (n = 20 males/pool) were extracted using the RNA Trizol Extraction Kit (Invitrogen, USA). DNA and RNA quality were checked by Qubit (Life Technologies, USA). DNA and RNA integrity were examined by agarose gel electrophoresis.

### PacBio ISO-seq library construction and sequencing

The full-length ISO-seq libraries were constructed using total RNA. First-strand complementary DNA (cDNA) was synthesized using a ClontechSMARTer PCR cDNA Synthesis Kit (ClonTech, Takara Bio Inc., Japan) with anchored oligo(dT) as the primer. Double-stranded cDNA was generated by large-scale PCR using an optimized PCR cycle number. Separation of different cDNA fractions by length was generated using the BluePippin Size Selection System. Once double-stranded cDNA was prepared, the SMRTbell libraries were constructed using the PacBio SMRTbell Template Prep Kit 1.0 (Pacific Biosciences, USA) following the vendor's protocols. Three SMRT RNA libraries, 1–2 k, 2–3 k, and 3–6 k, were prepared for worker and gyne samples. Mixed libraries without size selection were prepared for queen and male samples as the protocol improved. The SMRTbell libraries were then sequenced on the PacBio Sequel platform (PacBio Sequel System, RRID:SCR_017989).

### RNA library construction and sequencing

In parallel, RNA sequencing of male brains was performed by constructing a Micro-Tn5 Transposon Library following the methods described by Zhu et al. [58] and sequencing on the BGISEQ-500 PE100 platform (BGISEQ-500, RRID:SCR_017979). The RNA-seq data from workers, gynes, and queens were requested from Qiu et al. [57].

### Genome sequencing

To achieve a high-quality pharaoh ant genome assembly, we adopted a combination of sequencing methods including Illumina and PacBio sequencing.

For Illumina sequencing, 3 short-insert-sized DNA libraries (250, 500, and 800 bp) were constructed using an Illumina TruSeq Nano DNA LibraryPrep Kit (Illumina, USA) following the manufacturer's instructions, and then sequenced on an Illumina Hiseq 2000 instrument using a whole-genome shotgun sequencing strategy at BGI-Shenzhen (Shenzhen, China). We obtained a total of 33 Gb of clean data with ∼103-fold sequencing depth.

For PacBio sequencing, the BluePippin Size-Selection System was used to perform size selection. In total, DNA was sheared to a ∼20-kb targeted size using ultrasonication (Covais, Woburn, MA, USA), with a final 20-kb DNA fragment retained to construct the libraries. The constructed libraries were sequenced using the PacBio Sequel system at Novogene (Tianjin, China), and a total of 12 SMRT cells were used to yield 31 Gb of sequencing subreads with an average length of 7.5 kb and N50 of 11.6 kb.

### Genome assembly

#### Genome size estimation

We estimated the size of the pharaoh ant genome using routine 17-mer frequency analysis [25]. The genome size was estimated according to the formula: genome size = No. *k*-mers/peak of depth.

#### Genome assembly by PacBio long reads

We used an in-house pipeline to perform genome assembly, which included 5 steps:

1. Contig construction
  Canu (Canu, RRID:SCR_015880) v1.5 was used for 96-fold PacBio Sequel read assembly with default parameters and the complete Canu pipeline. For the Canu assembly, contig N50 was 1.26 Mb and total assembly size was 323 Mb.
2. Linking contigs to scaffold
  Scaffolding was performed using the SSPACE long-read scaffolder. The SSPACE-LongRead uses the BLASR aligner, which aligned the long-read set to the Canu contig assembly. We improved assembly contiguity and acquired a larger scaffold N50 than that obtained via the Canu contig assembly.
3. Filling gaps within scaffolds
  After scaffolding, PBJelly (PBJelly, RRID:SCR_012091) was used to fill the gaps within the scaffold using the PacBio sequences. The running parameters were as follows: -minMatch 8 -sdpTupleSize 8 -minPctIdentity 75 -bestn 1 -nCandidates 10 -maxScore -500 -nproc 13 -noSplitSubreads. Most gaps were filled in this step. This resulted in an assembly of 325 Mb, with a scaffold N50 of 3.63 Mb, contig N50 of 2.63 Mb, and number of undetermined bases (Ns) of 284 kb (0.08% of total genome assembly). Thus, contig N50 showed marked improvement (2 times) compared with the Canu contig assembly.
4. Two rounds of genome assembly polishing
  Because the PacBio raw reads have high sequencing error rates, we performed 2 rounds of genome assembly polishing. In the first round, Arrow software was used to map the PacBio sequences to the genome assembly. Small insertions/deletions (indels) and substitutions were then corrected, and consensus sequences were obtained. We performed the second round of polishing using high-quality Illumina paired-end short reads. First, the Illumina short reads were mapped to the assembly using BWA (BWA, RRID:SCR_010910), after which Pilon (Pilon, RRID:SCR_014731) was used to correct the sequences by input BAM alignments and assembly sequences. The parameters were as follows: “–changes –vcf –diploid –fix bases –mindepth 8.” Results showed that Pilon corrected 14,680 substitutions, 46,245 small insertions, and 9 ,410 small deletions for the raw read PacBio genome assembly.
5. Removal of contaminated sequences, duplicated haplotigs, and artifacts
  By aligning the genome sequences against the Bacteria and Virus databases using BLAST (-e 1e−5), we obtained a total length of 2,424,757 bp contaminated sequences, which accounted for 0.75% of the genome sequences. The most frequently aligned bacteria were endosymbionts of insects, such as *Wolbachia, Bacillus*, and *Candidatus*. We filtered out the contaminated contigs with contaminated sequences ≥20%. We did not find virus contamination in the genome sequences. Altogether, we filtered out 151,589 bp of contaminated sequences.

Purge_haplotigs was used to resolve duplicated haplotigs and artifacts in the genome assembly. First, we remapped the PacBio long reads to the genome assembly using minimap2. Purge_haplotigs was then used to calculate sequencing depth based on BEDtools (BEDTools, RRID:SCR_006646) [59] and generate a read-depth histogram. We chose 3 cut-offs (depths of 10, 25, and 85) to capture potential duplicated regions and haplotype-fused regions. Finally, purge_haplotigs filtered out 12,469,513 bp of haplotigs and artifacts. The final PacBio read-based genome assembly of the pharaoh ant was 312,903,204 bp.

#### *In situ* Hi-C library preparation and chromosome assembly

To establish the chromosome-level reference genome, worker pharaoh ants were used to construct a Hi-C library by modifying the protocol in Rao et al. [60]. The library was sequenced on the BGISEQ-500 platform under 100 paired-end mode. We used HiC-Pro (HiC-Pro, RRID:SCR_017643) to filter invalid read pairs, such as self-ligation, non-ligation, start-nearRsite, PCR amplification, random break, largeSmallFragments, and ExtremeFragments. The valid read pairs were mapped to the polished pharaoh ant genome. The contact count between contigs was calculated and normalized by restriction sites in sequences. We successfully produced 11 chromosomes, which occupied 94% of the genome, using the 3D-DNA pipeline. The 11 chromosomes were consistent with previous karyotype analyses of the pharaoh ant [37].

#### Genome assembly evaluation

To assess base quality of the whole-genome assembly, we first aligned the high-quality Illumina short reads to the final base error-corrected assembly. The percentage of total mapped reads was 97%. We then used the variant detector FreeBayes to calculate the homozygous variant ratio by inputting the BWA alignments. We detected the homozygous variants with parameters “-C 2 -0 -O -q 20 -z 0.10 -E 0 -X -u -p 2 -F 0.6,” as per Jain et al. [31]. The homozygous variations were derived from base-calling errors as the genome is diploid. The error rate was calculated as 0.001%, indicating a base QV of 50. The QV and identity were calculated using the algorithm in Jain et al. [31]. For protein-coding gene regions, we ran BUSCO (BUSCO, RRID:SCR_015008) on the genome mode to search for conserved genes in Hymenoptera species.

### Genome annotation

#### Annotation of repeat DNA sequences

1. Identification of known transposable elements (TEs)
  We first identified known TEs in the pharaoh ant genome using RepeatMasker (RepeatMasker, RRID:SCR_012954) by searching against the Repbase (v20.04) TE library [61]. We then used RepeatProteinMask within the RepeatMasker package to search the TE protein database.
2. *De novo* repeat prediction
  A *de novo* repeat library using RepeatModeler v. open-1.0.8 (RepeatModeler, RRID:SCR_015027) [62] was first generated, after which the TEs were annotated by RepeatMasker using the *de novo* repeat library.
3. Tandem repeats
  We also predicted tandem repeats using TRF, with the parameters following: “Math = 2, Mismatch = 7, Delta = 7, PM = 80, PI = 10, Minscore = 50, and MaxPeriod = 12.”

### Protein-coding gene prediction and functional annotation

#### Combined homology-, *de novo*–, and RNA-seq–based gene predictions

Combined homology-, *de novo–*, and transcriptome-RNA-seq–based gene predictions were used to annotate the protein-coding sequences in the pharaoh ant genome, as used in our previous study on the leopard gecko [63].

For the homology-based method, reference gene sets of *D. melanogaster, A. mellifera, Linepithema humile, N. vitripennis, Solenopsis invicta*, and *M. pharaonis* from the Ensembl and NCBI databases were used. We used the same parameters and methods as for the leopard gecko [63].

For *de novo* prediction of the pharaoh ant genome, methods and parameters were the same as used for the leopard gecko [63].

The transcriptome-RNA-seq–based method was performed using the pharaoh ant RNA-seq data from the brains of different castes and other tissues downloaded from the NCBI database (NCBI accession Nos. DRR032044–DRR032266). TopHat v1.3.3 (TopHat, RRID:SCR_013035) was used to identify splice junctions (SJs) by aligning the RNA-seq reads to the pharaoh ant genome. Cufflinks v2.2.1 (Cufflinks, RRID:SCR_014597) was applied to assemble transcripts using the aligned RNA-seq reads. After that, we built non-redundant reference gene sets based on a priority order of transcriptome-based evidence > homology-based evidence > *de novo*–based evidence to combine gene evidence using the in-house script from Xiong et al. [63]. At this step, a total of 15,576 non-redundant protein-coding genes were annotated.

#### ISO-seq isoforms improve gene model prediction

The ISO-seq approach can improve gene annotations in eukaryotic genomes. We incorporated the ISO-seq data to improve the gene model predicted in the previous step. We first compared the location of PacBio isoforms with the reference gene location using gffcompare. The overlapping PacBio isoforms on the same strand as the reference gene loci were used to refine the gene models, introduce AS events, and update the annotations of UTRs. We modified incorrect gene models caused by incorrect gene prediction. To further investigate missing or incomplete protein-coding gene models, a Markov model was estimated with 1,000 high-quality genes using the trainGlimmerHMM tool included in the GlimmerHMM software package. The putative protein-coding sequence of each PacBio isoform was identified using the Markov model. Finally, by comparing the gene models to the reference genome, we generated 15,327 protein-coding genes, which was the final predicted gene set.

#### Gene function annotation

Functional annotation of protein-coding genes was performed by searching against function databases, including COG, TrEMBL, SwissProt, and KEGG, using BLASTP. InterProScan (v5.16) with 7 different models (Profilescan, blastprodom, HmmSmart, HmmPanther, HmmPfam, FPrintScan, and Pattern-Scan) was used to annotate the protein domains and motifs.

### ISO-seq analysis

#### Transcriptome analysis pipeline for ISO-seq

We ran ISO-seq analysis using SMRT Link v5.0 [64] on the command line via pbsmrtpipe [65] to obtain the high-quality PacBio isoform dataset. Analysis included the following 4 steps:

1. Circular consensus sequence (CCS) identification
  CCSs were created from the raw subreads of PacBio sequences using CCS software (v3.0.0) within the pbsmrtpipe package. The CCS software takes multiple reads of the same SMRTbell sequence and combines them using a statistical model to produce 1 high-quality consensus sequence.
2. Classification of CCSs to full-length reads
  CCSs were classified as full-length non-chimeric and non–full-length reads. This was done by identifying the 5′ and 3′ adapters used in the library preparation, as well as the poly(A) tail. A read was considered full length if both primers were detected at the ends with a poly(A) tail signal of ≥12 consecutive “A's preceding the 3” primer. This step also removed primers and polyA/T tails accordingly.
3. Clustering of sequences based on similarity
  Isoform-level clustering was performed by using the Iterative Clustering and Error correction [11] algorithm and clustering the classified transcript sequences on the basis of similarity. For each cluster, the consensus transcripts were obtained.
4. Error correction polishing of isoforms
  The error correction Arrow software in the pbsmrtpipe package was used to polish the consensus sequences generated from the transcript clustering step. Arrow mapped PacBio raw reads to obtain the consensus and variant calls. This output polished high-quality (predicted accuracy ≥99%) full-length isoform consensus sequences, as well as low-quality isoform consensus sequences.
5. Alignment of isoforms to reference genome
  We used GMAP to align the isoform consensus sequences to the genome assembly with parameters “-f samse -n 0“. A Python script from the PacBio repository [66] was then used to predict the transcript structure and remove redundant transcripts. Each isoform was compared with the reference annotation by gffcompare and the isoforms were further classified into 8 groups based on their exon structures.

#### Rarefaction analysis of ISO-seq data

To investigate whether the sequencing depth of those data was sufficient to capture most of the transcriptome of interest, we performed rarefaction analysis on all data from the 4 caste sample libraries. We first pooled all sequencing data of the caste samples to reach a total of 58.8 Gb subreads. We then randomly selected 10%, 20%, 30%, …, 100% of total subreads to perform similar ISO-seq analyses to measure the (i) number of consensus transcripts, (ii) genome coverage, (iii) number of total isoforms, (iv) number of detected expressed genes, (v) AS events, and (vi) detectable genes with AS. All saturation curves were plotted using ggplot2 in the R package.

#### Identification of AS events

To verify the PacBio transcript isoforms, we analyzed the isoforms in relation to their SJs. The SJs could be divided into canonical and non-canonical according to the 2 pairs of dinucleotides present at the beginning and end of the introns encompassed by the junctions. The canonical SJs (GT-AG) accounted for ∼95% of all introns of the pharaoh ant PacBio isoforms. We also investigated the consistency of SJs between the RNA-seq and ISO-seq data. STAR (v2.4.0) was used to map the RNA-seq data to the reference genome, and all SJs were detected.

We used a Python script (alternative_splice.py) to detect AS events following Wang et al. [67]. This method was specifically designed to determine AS events using ISO-seq data and has been used in various ISO-seq studies [67–69]. The script uniquely designates all possible splicing patterns as an AS code according to the relative position of the alternative splice sites involved in the splicing variation. Five main modes of AS (intron retention, exon skipping, alternative 3′-acceptor, alternative 5′-donor, and alternative position [both 5′-donor and 3′-acceptor]) were identified. We visualized AS types using SVG implemented in Perl. We then compared the AS type variation among the 4 castes using a custom script.

#### Discovery of caste-specific AS isoforms among 4 castes

To investigate differential AS isoforms from the PacBio isoforms among the 4 castes, we used the scripts from the Cupcake package [66] to chain the isoforms together across the caste samples with default parameters. The isoforms from different caste samples that had an exact match for every exon boundary were chained together. Caste-specific isoforms were defined if the isoforms only existed in a unique caste sample. The caste-specific isoforms were compared with the AS isoform dataset, and those containing AS events were defined as caste-specific AS isoforms.

#### LncRNA identification from PacBio sequences

We identified lncRNAs from PacBio ISO-seq datasets using a customized pipeline comprising 4 steps: (i) The PacBio isoforms were aligned to gene models in the pharaoh ant genome. Isoforms that could not be aligned were considered novel sequences. We extracted the loci of novel sequences that did not overlap with the reference annotation or overlapped with the reference annotation but on the opposite strand. (ii) To filter out the potential coding sequences, we used BLAST to screen the sequences for homology with pharaoh ant proteins, and proteins from the functional database (UniProt). (iii) The CPC, PLEK, and CPAT programs were used to discriminate non-coding sequences from protein-coding genes. Sequences predicted as non-coding by all 3 software packages were deemed candidate lncRNAs. (iv) To eliminate the possible effects of transcription or splicing noise on the identification of lncRNAs, we filtered out those lncRNAs that were supported by <2 full-length PacBio sequencing reads.

#### Identification and characterization of conserved lncRNAs

Conserved lncRNAs within ants were identified by screening the annotated lncRNAs in highly conserved non-coding elements (CNEs) between ant genomes. The identification method was as follows: (i) We performed pair-wise whole-genome alignment using Lastz between the pharaoh ant genome and 3 published PacBio genomes (*C. floridanus, H. saltator*, and *O. biroi*) downloaded from the NCBI. Multiple alignments of the 4 ant genomes were then generated using Multiz, with the pharaoh ant as the reference. (ii) We used PhaseCons to estimate the genome conservation index and then identified the highly conserved elements (HCEs). Briefly, we used phyloFit to estimate an initial neutral phylogenetic model. We then ran PhastCons twice, first for estimation of conserved and non-conserved models and then for prediction of conserved elements. Finally, we identified 408,113 ant HCEs, covering 56 Mb of the pharaoh ant genome. (iii) We filtered the HCEs located in the protein-coding regions, resulting in 323,193 CNEs covering 32 Mb of the pharaoh ant genome. (iv) Finally, the annotated lncRNAs located in the CNEs were considered to be ant-conserved lncRNAs. Our analysis revealed a total of 961 conserved ant lncRNAs.

We also performed orthologous analysis of lncRNAs between ants and parasitoid wasps (*N. vitripennis*), ants and bees (*A. mellifera*), and ants and flies (*D. melanogaster*). We first performed whole-genome alignment among these genomes. The bee, parasitoid wasp, and fly genomes were each aligned to the pharaoh ant genome using Lastz. We then used liftOver (liftOver, RRID:SCR_018160) to compare the genome coordinates of ant-conserved lncRNAs to the wasp, bee, and fly genomes according to the “chain” alignment blocks, which are “chained” based on their location in both genomes. We used liftOver with default parameters. The ant lncRNAs located within or overlapping with conserved bee/wasp/fly genome regions were considered to be conserved insect lncRNAs.

#### Identification of differentially expressed lncRNAs between worker and gyne using Illumina RNA-seq data

The Illumina RNA-seq data from brain tissues of worker and gyne in Qiu et al. [57] were generated from the same batch, with each caste comprising 5 biological replicates. These data allowed us to identify differentially expressed lncRNAs between the 2 castes. First, ISO-seq transcriptome quantifications were performed with the Salmon pipeline (version 1.3.0) using RNA-seq data of the 10 samples, respectively. In brief, RNA-seq data from the brain samples of worker and gyne were quasi-mapped to the ISO-seq transcriptome, after which bias correction options were turned on to account for GC bias and sequence-specific bias. Isoform expression level was estimated as transcripts per million. DESeq2 version 1.16.1 (DESeq2, RRID:SCR_015687) was subsequently used to determine the differentially expressed isoforms between worker and gyne samples. The lncRNA transcripts were classified as differentially expressed between castes when the FDR-adjusted *P*-value was ≤0.05. Finally, we identified 32 conserved lncRNAs from the differentially expressed isoforms, in which caste affects the lncRNA expression level significantly.

## Data Availability

SMRT sequencing data, Illumina HiSeq data, and BGI-seq data generated in this study can be accessed through the NCBI SRA under accession No. PRJNA634441. Other data generated and analyzed during this study are available on Mendeley Data [70]. All supporting data and materials are available in the *GigaScience* GigaDB database [71]. The data reported in this study are also available in the CNGB Sequence Archive (CNSA) [72] of China National GeneBank DataBase (CNGBdb) [73] with accession No. CNP0001417.

## Additional Files

**Supplementary Fig. S1:** Frequency distribution of 17-mer analysis. The 17-mers were counted from a subset of paired-end reads from 800-bp libraries. Peak depth is 18×. Total number of 17-mers present in this subset was 6,154,945,619. Genome size, estimated by dividing total number of 17-mers by peak depth, was 342 Mb.

**Supplementary Fig. S2:** Example showing full-length PacBio isoform supported by short RNA-seq reads.

**Supplementary Fig. S3:** Sex-specific splicing of *fem* in pharaoh ant. Results showed that the full-length transcript with all coding exons was only expressed in female castes.

**Supplementary Fig. S4:** Caste-specific isoforms and dominant AS isoforms of *crlf3* in pharaoh ant.

**Supplementary Table S1:** Statistics of 17-mer analysis.

**Supplementary Table S2:** Statistics of Illumina and PacBio sequencing data for *M. pharaonis*. Data were produced by short/long insert-sized libraries. Sequencing depth was calculated by assembled genome size.

**Supplementary Table S3:** PacBio assembly statistics at different stages.

**Supplementary Table S4:** Statistics of assembled pharaoh ant chromosome.

**Supplementary Table S5:** Twenty-seven ant species for which sequenced genomes are available, in alphabetical order. Modified from Boomsma et al. [21].

**Supplementary Table S6:** Status of high GC content genes in short-read assembly.

**Supplementary Table S7:** Summary of ISO-seq data from different castes of pharaoh ant.

**Supplementary Table S8:** Summary of RNA-seq data.

**Supplementary Table S9:** Statistics of genes corrected by ISO-seq data.

**Supplementary Table S10:** Statistics of consensus transcripts mapped to genome.

**Supplementary Table S11:** Summary of splice junctions among 4 castes.

**Supplementary Table S12:** Summary of alternative splicing (AS) events in 4 castes.

**Supplementary Table S13:** Gene ontology (GO) enrichment analysis for isoform-rich genes in pharaoh ant.

**Supplementary Table S14:** KEGG analysis of caste-specific isoforms in worker.

**Supplementary Table S15:** KEGG analysis of caste-specific isoforms in gyne.

**Supplementary Table S16:** KEGG analysis of caste-specific isoforms in queen.

**Supplementary Table S17:** KEGG analysis of caste-specific isoforms in male.

**Supplementary Table S18:** Summary of genes with caste-specific dominant AS isoforms in 4 castes.

**Supplementary Table S19:** Gene ontology (GO) enrichment analysis of genes with caste-specific dominant AS isoforms.

## Abbreviations

AltA: alternative acceptor site; AltD: alternative donor site; AltP: alternative position; AS: alternative splicing; BLAST: Basic Local Alignment Search Tool; bp: base pairs; BUSCO: Benchmarking Universal Single-Copy Orthologs; BWA: Burrows-Wheeler Aligner; CCS: circular consensus sequence; cDNA: complementary DNA; CDS: coding sequence; CNEs: conserved non-coding elements; COG: Clusters of Orthologous Groups; ES: exon skipping; FDR: false discovery rate; GAGA: Global Ant Genomics Alliance; Gb: gigabase pairs; GC: guanine-cytosine; GMAP: Genomic Mapping and Alignment Program; GO: Gene Ontology; HCE: highly conserved element; Hi-C: high-throughput chromosome conformation capture; IR: intron retention; ISO-seq: isoform sequencing; kb: kilobase pairs; KEGG: Kyoto Encyclopedia of Genes and Genomes; lncRNA: long non-coding RNA; Mb: megabase pairs; NCBI: National Center for Biotechnology Information; NR: Non-Redundant database; PacBio: Pacific Biosciences; PBS: phosphate-buffered saline; QV: quality value; RNA-seq: RNA sequencing; SJ: splice junction; SMRT: Single Molecule Real Time; SRA: Sequence Read Archive; TE: transposable element; TRF: Tandem Repeats Finder; UTR: untranslated region.

## Competing Interests

Z.X., L.Z., G.D., and G.Z. are employees of BGI. The authors declare that they have no other competing interests.

## Funding

This work was supported by the National Natural Science Foundation of China (31970573), Lundbeck Foundation (R190–2014-2827) to G.Z., and Postdoctoral Research Foundation of China (2017M623081) and Funding for Postdoctoral Orientation Training in Yunnan province to Q.G.

## Authors' Contributions

G.Z. conceived and designed the study. Q.G., Z.X., and J.Z. collected the samples, Q.G. extracted the DNA and RNA, Z.X. performed the overall genome assembly and transcriptome analysis, R.S.L. prepared the Hi-C library, L.Z. conducted chromosomal genome assembly, and Q.G., Z.X., and G.Z. wrote the manuscript. All authors read and wrote part of the manuscript.

## Supplementary Material

giaa143_GIGA-D-20-00148_Original_Submission

giaa143_GIGA-D-20-00148_Revision_1

giaa143_GIGA-D-20-00148_Revision_2

giaa143_Response_to_Reviewer_Comments_Original_Submission

giaa143_Response_to_Reviewer_Comments_Revision_1

giaa143_Reviewer_1_Report_Original_Submission Libbrecht -- 6/30/2020 Reviewed

giaa143_Reviewer_1_Report_Revision_1 Libbrecht -- 9/30/2020 Reviewed

giaa143_Reviewer_2_Report_Original_Submission John Wang -- 7/1/2020 Reviewed

giaa143_Supplemental_Figures_and_Tables

## References

[1] Libbrecht R, Oxley PR, Kronauer DJ, et al. Ant genomics sheds light on the molecular regulation of social organization. Genome Biol. 2013;14(7):212. 23895728 10.1186/gb-2013-14-7-212 PMC4053786

[2] Hölldobler B, Wilson EO. The Superorganism. New York, London: Morton; 2009.

[3] Thorne BL . Evolution of eusociality in termites. Annu Rev Ecol Syst. 1997;28:27–54.

[4] Schwander T, Lo N, Beekman M, et al. Nature versus nurture in social insect caste differentiation. Trends Ecol Evol. 2010;25(5):275–82. 20106547 10.1016/j.tree.2009.12.001

[5] Corona M, Libbrecht R, Wurm Y, et al. Vitellogenin underwent subfunctionalization to acquire caste and behavioral specific expression in the harvester ant *Pogonomyrmex barbatus*. PLos Genet. 2013;9(8):e1003730. 23966882 10.1371/journal.pgen.1003730 PMC3744404

[6] Ingram KK, Krummey S, LeRoux M. Expression patterns of a circadian clock gene are associated with age-related polyethism in harvester ants, *Pogonomyrmex occidentalis*. BMC Ecol. 2009;9:7. 19374755 10.1186/1472-6785-9-7 PMC2676274

[7] Ingram KK, Kleeman L, Peteru S. Differential regulation of the*foraging* gene associated with task behaviors in harvester ants. BMC Ecol. 2011;11:19. 21831307 10.1186/1472-6785-11-19 PMC3180247

[8] Morandin C, Havukainen H, Kulmuni J, et al. Not only for egg yolk—functional and evolutionary insights from expression, selection, and structural analyses of *Formica* ant vitellogenins. Mol Biol Evol. 2014;31(8):2181–93. 24895411 10.1093/molbev/msu171

[9] Harrison MC, Hammond RL, Mallon EB. Reproductive workers show queenlike gene expression in an intermediately eusocial insect, the buff-tailed bumble bee *Bombus terrestris*. Mol Ecol. 2015;24(12):3043–63. 25913260 10.1111/mec.13215

[10] Friedman DA, Gordon DM. Ant genetics: reproductive physiology, worker morphology, and behavior. Annu Rev Neurosci. 2016;39:41–56. 27050321 10.1146/annurev-neuro-070815-013927

[11] Price J, Harrison M, Hammond R, et al. Alternative splicing associated with phenotypic plasticity in the bumble bee *Bombus terrestris*. Mol Ecol. 2018;27(4):1036–43. 29377451 10.1111/mec.14495

[12] Wurm Y, Wang J, Riba-Grognuz O, et al. The genome of the fire ant *Solenopsis invicta*. Proc Natl Acad Sci U S A. 2011;108(14):5679–84. 21282665 10.1073/pnas.1009690108 PMC3078418

[13] Foret S, Kucharski R, Pellegrini M, et al. DNA methylation dynamics, metabolic fluxes, gene splicing, and alternative phenotypes in honey bees. Proc Natl Acad Sci U S A. 2012;109(13):4968–73. 22416128 10.1073/pnas.1202392109 PMC3324026

[14] Li-Byarlay H, Li Y, Stroud H, et al. RNA interference knockdown of DNA methyl-transferase 3 affects gene alternative splicing in the honey bee. Proc Natl Acad Sci U S A. 2013;110(31):12750–5. 23852726 10.1073/pnas.1310735110 PMC3732956

[15] Terrapon N, Li C, Robertson HM, et al. Molecular traces of alternative social organization in a termite genome. Nat Commun. 2014;5:3636. 24845553 10.1038/ncomms4636

[16] Bonasio R, Li Q, Lian J, et al. Genome-wide and caste-specific DNA methylomes of the ants *Camponotus floridanus* and *Harpegnathos saltator*. Curr Biol. 2012;22(19):1755–64. 22885060 10.1016/j.cub.2012.07.042 PMC3498763

[17] Yan H, Bonasio R, Simola DF, et al. DNA methylation in social insects: how epigenetics can control behavior and longevity. Annu Rev Entomol. 2015;60:435–52. 25341091 10.1146/annurev-ento-010814-020803

[18] Bonasio R, Tu S, Reinberg D. Molecular signals of epigenetic states. Science. 2010;330(6004):612–6. 21030644 10.1126/science.1191078 PMC3772643

[19] Simola DF, Wissler L, Donahue G, et al. Social insect genomes exhibit dramatic evolution in gene composition and regulation while preserving regulatory features linked to sociality. Genome Res. 2013;23(8):1235–47. 23636946 10.1101/gr.155408.113 PMC3730098

[20] Shields EJ, Sheng L, Weiner AK, et al. High-quality genome assemblies reveal long non-coding RNAs expressed in ant brains. Cell Rep. 2018;23(10):3078–90. 29874592 10.1016/j.celrep.2018.05.014 PMC6023404

[21] Boomsma JJ, Brady SG, Dunn RR, et al. The Global Ant Genomics Alliance (GAGA), Myrmecol News. 2017, 25, 61–66.

[22] Kornblihtt AR, Schor IE, Allo M, et al. Alternative splicing: a pivotal step between eukaryotic transcription and translation. Nat Rev Mol Cell Biol. 2013;14(3):153–65. 23385723 10.1038/nrm3525

[23] Madoui MA, Engelen S, Cruaud C, et al. Genome assembly using Nanopore-guided long and error-free DNA reads. BMC Genomics. 2015;16:327. 25927464 10.1186/s12864-015-1519-z PMC4460631

[24] Mikheyev AS, Linksvayer TA. Genes associated with ant social behavior show distinct transcriptional and evolutionary patterns. Elife. 2015;4:e04775. 25621766 10.7554/eLife.04775 PMC4383337

[25] Marcais G, Kingsford C. A fast, lock-free approach for efficient parallel counting of occurrences of k-mers. Bioinformatics. 2011;27(6):764–70. 21217122 10.1093/bioinformatics/btr011 PMC3051319

[26] Koren S, Walenz BP, Berlin K, et al. Canu: scalable and accurate long-read assembly via adaptive k-mer weighting and repeat separation. Genome Res. 2017;27(5):722–36. 28298431 10.1101/gr.215087.116 PMC5411767

[27] Boetzer M, Pirovano W. SSPACE-LongRead: scaffolding bacterial draft genomes using long read sequence information. BMC Bioinformatics. 2014;15:211. 24950923 10.1186/1471-2105-15-211 PMC4076250

[28] English AC, Richards S, Han Y, et al. Mind the gap: upgrading genomes with Pacific Biosciences RS long-read sequencing technology. PLoS One. 2012;7(11):e47768. 23185243 10.1371/journal.pone.0047768 PMC3504050

[29] Roach MJ, Schmidt SA, Borneman AR. Purge Haplotigs: allelic contig reassignment for third-gen diploid genome assemblies. BMC Bioinformatics. 2018;19(1):460. 30497373 10.1186/s12859-018-2485-7 PMC6267036

[30] Quality Value (QV) Scores. https://www.ucalgary.ca/dnalab/sequencing/services/QV. Accessed 13 October 2019.

[31] Jain M, Koren S, Miga KH, et al. Nanopore sequencing and assembly of a human genome with ultra-long reads. Nat Biotechnol. 2018;36(4):338–45. 29431738 10.1038/nbt.4060 PMC5889714

[32] Lieberman-Aiden E, van Berkum NL, Williams L, et al. Comprehensive mapping of long-range interactions reveals folding principles of the human genome. Science. 2009;326(5950):289–93. 19815776 10.1126/science.1181369 PMC2858594

[33] Durand N, Shamim M, Machol I, et al. Juicer provides a one-click system for analyzing loop-resolution Hi-C experiments. Cell Syst. 2016;3(1):95–8. 27467249 10.1016/j.cels.2016.07.002 PMC5846465

[34] Servant N, Varoquaux N, Lajoie BR, et al. HiC-Pro: an optimized and flexible pipeline for Hi-C data processing. Genome Biol. 2015;16(1):259. 26619908 10.1186/s13059-015-0831-x PMC4665391

[35] Dudchenko O, Batra SS, Omer AD, et al. De novo assembly of the *Aedes aegypti* genome using Hi-C yields chromosome-length scaffolds. Science. 2017;356(6333):92. 28336562 10.1126/science.aal3327 PMC5635820

[36] O'Connor RE, Farre M, Joseph S, et al. Chromosome-level assembly reveals extensive rearrangement in saker falcon and budgerigar, but not ostrich, genomes. Genome Biol. 2018;19(1):171. 30355328 10.1186/s13059-018-1550-x PMC6201548

[37] Smith IC, Peacock A. XI.—The Cytology of Pharaoh's Ant, *Monomorium pharaonis* (L.). Proc R Soc Lond B Biol Sci. 1957;66(3):235–61.

[38] Imai H, Yosida T. Chromosome observations in Japanese ants. Annu Rep Natl Inst Genet. 1964;15:64–6.

[39] Imai H, Urbani CB, Kubota M, et al. Karyological survey of Indian ants. Jpn J Genet. 1984;59(1):1–32.

[40] Ranz JM, Casals F, Ruiz A. How malleable is the eukaryotic genome? Extreme rate of chromosomal rearrangement in the genus *Drosophila*. Genome Res. 2001;11(2):230–9. 11157786 10.1101/gr.162901 PMC311025

[41] Schmieder S, Colinet D, Poirie M. Tracing back the nascence of a new sex-determination pathway to the ancestor of bees and ants. Nat Commun. 2012;3:895. 22692538 10.1038/ncomms1898 PMC3621418

[42] Nygaard S, Zhang GJ, Schiott M, et al. The genome of the leaf-cutting ant *Acromyrmex echinatior* suggests key adaptations to advanced social life and fungus farming. Genome Res. 2011;21(8):1339–48. 21719571 10.1101/gr.121392.111 PMC3149500

[43] Zhao S, Zhang B, Kulski J. Impact of gene annotation on RNA-seq data analysis. In: Kulski J, ed. Next Generation Sequencing: Advances, Applications and Challenges. Rijeka: InTech; 2016, doi:10.5772/61197.

[44] Wu TD, Watanabe CK. GMAP: a genomic mapping and alignment program for mRNA and EST sequences. Bioinformatics. 2005;21(9):1859–75. 15728110 10.1093/bioinformatics/bti310

[45] Braunschweig U, Barbosa-Morais NL, Pan Q, et al. Widespread intron retention in mammals functionally tunes transcriptomes. Genome Res. 2014;24(11):1774–86. 25258385 10.1101/gr.177790.114 PMC4216919

[46] Weiner SA, Toth AL. Epigenetics in social insects: a new direction for understanding the evolution of castes. Genet Res Int. 2012;2012, doi:10.1155/2012/609810. PMC3335566 22567395

[47] Chandra V, Fetter-Pruneda I, Oxley PR, et al. Social regulation of insulin signaling and the evolution of eusociality in ants. Science. 2018;361(6400):398–402. 30049879 10.1126/science.aar5723 PMC6178808

[48] Chen X, Hu Y, Zheng H, et al. Transcriptome comparison between honey bee queen-and worker-destined larvae. Insect Biochem Mol Biol. 2012;42(9):665–73. 22659440 10.1016/j.ibmb.2012.05.004

[49] Verhulst EC, van de Zande L, Beukeboom LW. Insect sex determination: it all evolves around transformer. Curr Opin Genet Dev. 2010;20(4):376–83. 20570131 10.1016/j.gde.2010.05.001

[50] Hahn N, Knorr DY, Liebig J, et al. The insect ortholog of the human orphan cytokine receptor *CRLF3* is a neuroprotective erythropoietin receptor. Front Mol Neurosci. 2017;10:223. 28769759 10.3389/fnmol.2017.00223 PMC5509957

[51] Hahn N, Buschgens L, Schwedhelm-Domeyer N, et al. The orphan cytokine receptor *CRLF3* emerged with the origin of the nervous system and is a neuroprotective erythropoietin receptor in locusts. Front Mol Neurosci. 2019;12:251. 31680856 10.3389/fnmol.2019.00251 PMC6797617

[52] Losko M, Kotlinowski J, Jura J. Long noncoding RNAs in metabolic syndrome related disorders. Mediators Inflamm. 2016;2016, doi:10.1155/2016/5365209. PMC5110871 27881904

[53] McKenzie SK, Kronauer DJ. The genomic architecture and molecular evolution of ant odorant receptors. Genome Res. 2018;28(11):1757–65. 30249741 10.1101/gr.237123.118 PMC6211649

[54] Derrien T, Johnson R, Bussotti G, et al. The GENCODE v7 catalog of human long noncoding RNAs: analysis of their gene structure, evolution, and expression. Genome Res. 2012;22(9):1775–89. 22955988 10.1101/gr.132159.111 PMC3431493

[55] Marques AC, Ponting CP. Intergenic lncRNAs and the evolution of gene expression. Curr Opin Genet Dev. 2014;27:48–53. 24852186 10.1016/j.gde.2014.03.009

[56] Vance KW, Ponting CP. Transcriptional regulatory functions of nuclear long noncoding RNAs. Trends Genet. 2014;30(8):348–55. 24974018 10.1016/j.tig.2014.06.001 PMC4115187

[57] Qiu B, Larsen R, Chang N, et al. Towards reconstructing the ancestral brain gene-network regulating caste differentiation in ants. Nat Ecol Evol. 2018;2(11):1782–91. 30349091 10.1038/s41559-018-0689-x PMC6217981

[58] Zhu F, Chen M, Ye N, et al. Comparative performance of the BGISEQ-500 and Illumina HiSeq4000 sequencing platforms for transcriptome analysis in plants. Plant Methods. 2018;14(1):69. 30123314 10.1186/s13007-018-0337-0 PMC6088413

[59] Quinlan AR, Hall IM. BEDTools: a flexible suite of utilities for comparing genomic features. Bioinformatics. 2010;26(6):841–2. 20110278 10.1093/bioinformatics/btq033 PMC2832824

[60] Rao SS, Huntley MH, Durand NC, et al. A 3D map of the human genome at kilobase resolution reveals principles of chromatin looping. Cell. 2014;159(7):1665–80. 25497547 10.1016/j.cell.2014.11.021 PMC5635824

[61] RepeatMasker. http://repeatmasker.org/. Accessed 13 November 2018.

[62] RepeatModeler-1.0.8. http://www.repeatmasker.org/RepeatModeler/. Accessed 13 November 2018.

[63] Xiong Z, Li F, Li Q, et al. Draft genome of the leopard gecko, *Eublepharis macularius*. GigaSci. 2016;5(1):47. 10.1186/s13742-016-0151-4 PMC5080775 27784328

[64] Pacific Biosciences. https://www.pacb.com/support/software-downloads/. Accessed 15 November 2017.

[65] Pacific Biosciences/pbsmrtpipe. https://github.com/PacificBiosciences/pbsmrtpipe. Accessed 13 November 2018.

[66] Magdoll/cDNA_Cupcake. https://github.com/Magdoll/cDNA_Cupcake/blob/master/cupcake/tofu/collapse_isoforms_by_sam.py. Accessed 13 November 2018.

[67] Wang M, Wang P, Liang F, et al. A global survey of alternative splicing in allopolyploid cotton: landscape, complexity and regulation. New Phytol. 2018;217(1):163–78. 28892169 10.1111/nph.14762

[68] Ren L, Yan X, Gao X, et al. Maternal effects shape the alternative splicing of parental alleles in reciprocal cross hybrids of *Megalobrama amblycephala* x *Culter alburnus*. BMC Genomics. 2020;21(1):457. 32616060 10.1186/s12864-020-06866-7 PMC7330940

[69] Zhang Y, Dong W, Zhao X, et al. Transcriptomic analysis of differentially expressed genes and alternative splicing events associated with crassulacean acid metabolism in orchids. Hortic Plant J. 2019;5(6):268–80.

[70] Gao Q, Xiong Z, Larsen RS, et al. High-quality chromosome-level genome assembly and full-length transcriptome analysis of the pharaoh ant *Monomorium pharaonis*. Mendeley Data 2020, doi:10.17632/pgxhnytds4.1. PMC7736795 33319913

[71] Gao Q, Xiong Z, Larsen RS, et al. Supporting data for “High-quality chromosome-level genome assembly and full-length transcriptome analysis of the pharaoh ant *Monomorium pharaonis*.”. GigaScience Database. 2020; 10.5524/100827. PMC7736795 33319913

[72] Guo X, Chen F, Gao F, et al. CNSA: a data repository for archiving omics data. Database (Oxford). 2020;2020, doi:10.1093/database/baaa055. PMC7377928 32705130

[73] Chen F, You L, Yang F, et al. CNGBdb: China National GeneBank DataBase. Hereditas. 2020;42(8):799–809. 32952115 10.16288/j.yczz.20-080

