# Supplementary material for: High-quality chromosome-level genome assembly and full-length transcriptome analysis of the pharaoh ant Monomorium pharaonis
Source: Gigascience. 2020 Dec 15;9(12):giaa143. doi: 10.1093/gigascience/giaa143 (PMC7736795; doi:10.1093/gigascience/giaa143)
Supplement: giaa143_GIGA-D-20-00148_Original_Submission [file giaa143_giga-d-20-00148_original_submission.pdf]

GigaScience

A high-quality chromosome-level pharaoh ant genome assembly and full-length transcriptome provide insights on ant caste differentiation

--Manuscript Draft--

Manuscript Number:	GIGA-D-20-00148
Full Title:	A high-quality chromosome-level pharaoh ant genome assembly and full-length transcriptome provide insights on ant caste differentiation
Article Type:	Research
Funding Information:	National Natural Science Foundation of China (31970573) — Dr. Guojie Zhang<br>Lundbeck Foundation (R190-2014-2827) — Dr. Guojie Zhang<br>Postdoctoral Research Foundation of China (2017M623081) — Dr. Qionghua Gao<br>Funding for Postdoctoral Orientation Training in Yunnan province — Dr. Qionghua Gao
Abstract:	Background<br>Caste differentiation associated with reproductive division of labor in social insects has fascinated scientists for centuries. Comparative genomic analyses across ant species and castes have revealed important insights onto the genomic mechanisms underlying caste differentiation. However, most of the current ant genomes are highly fragmented and incomplete, producing an impediment for understanding of genomic evolutionary changes particular on the regulatory regions and non-coding regions.<br>Findings<br>By hybridizing Illumina, PacBio, and Hi-C sequencing technologies, we de novo assembled a chromosome level genome for Monomorium pharaonis , with a scaffold N50 of 29.6 Mb and contig N50 of 18.6 Mb. Full genome comparison between ant species revealed high frequency of genomic rearrangements during ant evolution. Analyses with full-length isoform sequencing (ISO-seq) data suggested with ca. 15Gb ISO-seq data is sufficient to cover the majority of expressed genes but the number of transcript isoforms is steadily increased with the coverage of the sequencing data. The comparative transcriptome analysis across castes based on ISO-seq data revealed unprecedented large number of transcript isoforms including many caste-specific isoforms. We also identified a number of conserved lncRNAs across ant species and some of them are showing caste-specific expression, which might be crucial to caste development.<br>Conclusions<br>We produced a high-quality chromosome-level genome for M. pharaonis which significantly improved previous assembly with short reads. And together with full-length transcriptome for all castes, we generated a highly accurate annotation for this ant species. Our comparison across castes and ant species produced valuable resources on caste-specific transcription for future functional study.
Corresponding Author:	Guojie Zhang<br><br>DENMARK
Corresponding Author Secondary Information:	
Corresponding Author's Institution:	
Corresponding Author's Secondary Institution:	
First Author:	Qionghua Gao, Ph.D
First Author Secondary Information:	
Order of Authors:	Qionghua Gao, Ph.D

Powered by Editorial Manager® and ProduXion Manager® from Aries Systems Corporation

	Zijun Xiong
	Rasmus Stenbak Larsen
	Long Zhou
	Jie Zhao
	Guo Ding
	Ruoping Zhao
	Guojie Zhang
**Order of Authors Secondary Information:**	
**Additional Information:**	
**Question**	**Response**
Are you submitting this manuscript to a special series or article collection?	No
**Experimental design and statistics**<br><br>Full details of the experimental design and statistical methods used should be given in the Methods section, as detailed in our Minimum Standards Reporting Checklist. Information essential to interpreting the data presented should be made available in the figure legends.<br><br>Have you included all the information requested in your manuscript?	Yes
**Resources**<br><br>A description of all resources used, including antibodies, cell lines, animals and software tools, with enough information to allow them to be uniquely identified, should be included in the Methods section. Authors are strongly encouraged to cite Research Resource Identifiers (RRIDs) for antibodies, model organisms and tools, where possible.<br><br>Have you included the information requested as detailed in our Minimum Standards Reporting Checklist?	Yes
**Availability of data and materials**	Yes

All datasets and code on which the conclusions of the paper rely must be either included in your submission or deposited in publicly available repositories (where available and ethically appropriate), referencing such data using a unique identifier in the references and in the "Availability of Data and Materials" section of your manuscript.<br><br>Have you have met the above requirement as detailed in our Minimum Standards Reporting Checklist?	

Powered by Editorial Manager® and ProduXion Manager® from Aries Systems Corporation

Title: ***A high-quality chromosome-level pharaoh ant genome assembly and full-length transcriptome provide insights on ant caste differentiation***

Qionghua Gao[1, †], Zijun Xiong[1, 2, †], Rasmus Stenbak Larsen[3], Long Zhou[2], Jie Zhao[1], Guo Ding[1, 2, 3], Ruoping Zhao[1], Guojie Zhang[1, 2, 3, *]

[1] State Key Laboratory of Genetic Resources and Evolution, Kunming Institute of Zoology, Chinese Academy of Sciences, Kunming, Yunnan, 650223, China

[2] China National GeneBank, BGI-Shenzhen, Shenzhen, 518120, China

[3] Section for Ecology and Evolution, Department of Biology, University of Copenhagen, Copenhagen, DK-2100, Denmark

† These authors contributed equally

[*] **Corresponding author:** guojie.zhang@bio.ku.dk

E-mails:

Qionghua Gao: gaoqionghua123@163.com, Zijun Xiong: xiongzijun@genomics.cn, Rasmus Stenbak Larsen: rslarsen@bio.ku.dk, Long Zhou: zhoulong@genomics.cn, Jie Zhao: zhaojie@mail.kiz.ac.cn, Guo Ding: dzdingo@gmail.com, Ruoping Zhao: zhaorp@mail.kiz.ac.cn, Guojie Zhang: guojie.zhang@bio.ku.dk

Abstract

Background

Caste differentiation associated with reproductive division of labor in social insects has fascinated scientists for centuries. Comparative genomic analyses across ant species and castes have revealed important insights onto the genomic mechanisms underlying caste differentiation. However, most of the current ant genomes are highly fragmented and incomplete, producing an impediment for understanding of genomic evolutionary changes particular on the regulatory regions and non-coding regions.

Findings

By hybridizing Illumina, PacBio, and Hi-C sequencing technologies, we *de novo* assembled a chromosome level genome for *Monomorium pharaonis*, with a scaffold N50 of 29.6 Mb and contig N50 of 18.6 Mb. Full genome comparison between ant species revealed high frequency of genomic rearrangements during ant evolution. Analyses with full-length isoform sequencing (ISO-seq) data suggested with ca. 15Gb ISO-seq data is sufficient to cover the majority of expressed genes but the number of transcript isoforms is steadily increased with the coverage of the sequencing data. The comparative transcriptome analysis across castes based on ISO-seq data revealed unprecedented large number of transcript isoforms including many caste-specific isoforms. We also identified a number of conserved lncRNAs across ant species and some of them are showing caste-specific expression, which might be crucial to caste development.

Conclusions

We produced a high-quality chromosome-level genome for *M. pharaonis* which significantly

improved previous assembly with short reads. And together with full-length transcriptome for all castes, we generated a highly accurate annotation for this ant species. Our comparison across castes and ant species produced valuable resources on caste-specific transcription for future functional study.

Keywords: Social insects, *Monomorium pharaonis*, caste differentiation, long-read sequencing, alternative splicing, long non-coding RNA

Background

Ants are ecological diverse animal group and extraordinarily successful that occupy almost all terrestrial ecological niches [1]. As social insects, ants live in colonies composed of up to millions of individuals which develop into different social castes with remarkable reproductive division of labor and dramatic variations on morphology, physiology, and behaviors [2]. The sexual castes, including reproductively active queens, gynes (virgin queens), and males, are specialized for sexual reproduction, whereas the worker caste, in some species can be divided into distinct sub-castes, are specialized for non-reproductive roles to support the reproduction via constructing, maintaining and defending the nest, collecting food and rearing the brood [3].

Understanding the genetic mechanisms underlying the caste-development and differentiation process has been the major focus of recent studies on social insects. Previous studies showed that the caste differentiation involve the regulation with both genetic and epigenetic factors [4]. Comparative genome and transcriptome studies have identified several key genes show

different expression patterns between castes that might contribute to the caste-specific phenotype, such as *vitellogenin* [5], *foraging*, *arrestin*, and *insulin/insulin-like growth factor signaling* [1, 6-13]. Recent studies also suggested that alternative splicing as a source to increase genetic regulatory complexity that contributes to the phenotypic plasticity in eusocial insects [12, 14-17]. Additionally, epigenetic mechanisms such as long non-coding RNAs (lncRNAs), also participate on the gene expression regulation during the caste differentiation [18, 19]. Particularly, comparative genomic studies cross multiple ant lineages have identified many conserved lncRNAs that might play important roles in the evolution of caste system in ants [20, 21].

However, most previous genome relied on the short reads sequencing technology which often results in fragmented assembly with many sequencing gaps, which mainly due to high GC content or repeat sequences that are often fail to sequence with short reads technology. Additionally, the short reads-based RNA-seq is also fail to resolve complex isoforms with alternative splicing that ubiquitously present in eukaryotes. Single-molecule real-time (SMRT) long-read sequencing overcomes these limitations by generating ultra-long reads and offering different solutions to solve genome assembly problems such as complex regions with repeated elements or segmental duplications, or regions with high GC-contents [22]. Long-read sequencing is also beneficial in transcriptomics, which provides full length reads that spanning the entire transcript isoform, eliminating the need for transcript reconstruction and inference. Thus, ISO-seq can largely improve annotations of reference genomes, characterize gene isoforms in important genes, capture alternative splice variants and identify lncRNAs. Currently, only 27 ant genomes were published and notably much of them are in low quality

[23]. Therefore, high quality genome and full-length transcriptome are urgently needed to understand the molecular mechanisms under caste differentiation and reproductive division of labor.

The pharaoh ant *Monomorium pharaonis* (Fig. 1A) is an emerging model animal for genomic and molecular biology studies of social insects on caste differentiation. Unlike most of the ants, pharaoh ant has very short life span, easy to rear and can mate and reproduce within the colony, which makes it a perfect model organism for the genetic studies. The first draft genome of pharaoh ant was assembled with short reads [24], however it is very fragmented with scaffold N50 size at 75.38kb.

In this study, by using the PacBio SMRT DNA Sequencing and ISO-seq technology combined with the Illumina short-reads and Hi-C (High-through chromosome conformation capture) data, we produced a high quality chromosomal-level reference genome for pharaoh ant. And we also obtained a high-quality transcriptome across different ant castes, i.e. worker, gyne, queen and male castes. Based on these high-quality data, we further analyzed the protein coding genes, alternative spliced isoforms, and lncRNAs in different castes, which will enhance our understanding of the genetic and epigenetic mechanisms of the caste differentiation and reproductive division of labor of the eusocial insects.

Analyses

Genome assembly, assessment, and gene prediction

Based on the estimation of 17-mer analysis with short reads sequencing, the genome of *M. pharaonis* was estimated to be 342 Mb (Supplementary Fig. S1, Table S1). We generated 33

Gb (~103x) Illumina short reads sequencing data and over 31 Gb (~96x) of the PacBio sequencing data with total reads number of 4,151,307 (Supplementary Table S2). Genome of *M. pharaonis* was assembled to contigs by Canu using the PacBio Sequel sequencing data [25], and were Scaffolded by SSPACE_longRead scaffolder [26]. The assembled scaffolds were gap-filled with PBJelly program [27], and polished with the PacBio data and short sequencing reads using Quiver and Pilon, respectively (Supplementary Table S3, see Methods for the details). The polished assembly was 325 Mb with a scaffold N50 of 3.6 Mb (538 scaffolds), and contig N50 of 2.7 Mb (663 contigs) (Table 1). The phred quality value of the whole genome was calculated as QV50 (represents 99.999% accuracy), which suggests the assembly is of high quality [28].

**Reads**	**PacBio assembly**	**Hi-C assembly**
Genome assembly size (bp)	325,372,717	325,533,717
Number of scaffolds	538	726
Scaffold N50 (bp)	3,631,423	29,663,535
Scaffold N90 (bp)	683,524	16,858,934
Max scaffolds length (bp)	18,497,097	41,458,767
Number of contigs	663	1173
Contig N50 (bp)	2,718,975	1,861,574
Contig N90 (bp)	436,137	251,272
Max contig length (bp)	9,733,832	6,466,356
GC content (%)	36.34	36.34
BUSCO (n = 4415)	C: 98.4%, F: 1.1%	

C: complete BUSCOs; F: fragmented BUSCOs.

Table 1. Summary of the genome features of M. pharaonis

Hi-C uses high-throughput sequencing to map the genome-wide chromatin contacts thus has been widely used as scaffolding method in genome assembly [29]. We generated 14.82 Gb Hi-C sequencing data and mapped them to the polished pharaoh ant genome using Juicer

software [30] after filtering low quality data with Hic-Pro [31] to improve the connection integrity of the contigs. The locations and directions of contigs were determined by 3D *de novo* assembly (3d-DNA) software [32] with default parameters, then contigs were successfully clustered and anchored on 11 linkage groups (Fig. 1B, Supplementary Table S4), which covers 94% of the pharaoh ant assembled sequences. At last, we obtained a chromosomal-level high quality pharaoh ant genome with a contig N50 of 18.6 Mb and scaffold N50 of 29.6 Mb (Table 1).

Comparing with other twenty-seven published ant genomes, which were mostly sequenced and assembled with short reads sequencing, our pharaoh ant genome assembly shows significant higher contiguity level (Fig. 1C, Supplementary Table S5). Our new genome assembly with PacBio reads is also more complete than other published ant genomes with only 0.0867% of the new assembly are gap while on average 3.75% of other ant genomes have not been sequenced. Specifically, we compared the genomic regions with high GC-content and found that a large number of genomic regions with high GC-content had been missed in previous short reads assembly of pharaoh ant genome [24], but now have been covered with new assembly (Fig. 1D). 9.76% genes with over 70% GC-content (n = 4), and 11.30% (52 out of 460) of the genes with GC-content of 60~70% were completely missing in previous short-reads assembly but have been recovered by our assembly, indicating that PacBio assembly has significant advantages for the high GC-content genes (Supplementary Table S6). Furthermore, the completeness of our PacBio assembly was assessed by BUSCO, which showed that 98.4% of the total 4,415 expected Hymenoptera conserved genes were identified as complete (Table 1).

Gene prediction was done by combining homology-based, *de novo*, and RNA-seq transcriptome-based searches and identification method (see Methods for the details). A total of 15,945 non-redundant protein-coding genes were predicted in the pharaoh ant genome assembly. By searching against the functional databases (i.e., TrEMBL, COG, SwissProt, GO, and KEGG, and annotating using InterProScan), we annotated 15,858 (99.45%) genes and identified 14,399 (90.30%) genes with conserved motifs (Table 2).

	Number	Percent(%)
Total	15,945	
InterPro	14,399	90.30
COG	4,933	30.94
GO	8,920	55.94
KEGG	13,338	83.65
Swissprot	11,078	69.48
TrEMBL	15,846	99.38
Annotated	15,858	99.45
Unanotated	87	0.55

Table 2. Statistics of functional annotation of protein-coding genes in pharaoh ant

Ant genome experienced high frequency of chromosome recombination

Previous studies have suggested ant genomes have experienced fast genomic rearrangement rate [20]. To provide a chromosome level landscape of genomic rearrangement across ant species, we performed genome collinearity analyses between the chromosomal level assembled pharaoh ant genome (2n = 22) [33, 34] and the genome of clonal raider ant *Ooceraea biroi* (2n = 28) [5]. The synteny map spans 14 *O. biroi* (Obir) chromosomes and 11 *M. pharaonis* (Mpha) chromosomes, which covering 94% of Mpha genome (Fig. 2A). We detected about 150 fissions/fusions occurred at the chromosome level with >500kb block

resolution between two species, suggesting that the drastic genome rearrangements occurred during the genome evolution.

To detail the micro-synteny evolution pattern across ant lineages, we investigated the orthologs of genes in upstream and downstream of *fem* and *csd* across 11 ant species by accessing their reference genomes recently produced by Global Ant Genomic Consortium and 2 wasps from NCBI. *Complementary sex determiner* (*csd*) is the primary sex-determining signal in most of the eusocial Hymenoptera, and is arisen from the duplication of the *feminizer* (*fem*) gene, which plays a key role in sex determination [35, 36]. By performing the synteny analysis of *fem* and *csd* and their neighbor genes, we found that *fem* presented in all investigated species, however its synteny with neighbor genes experienced several times of translocation and recombination during the diversification of ant lineages (Fig. 2B). Whereas, not all the ants have the *csd* homolog and they are placed in different genomic locations in many ant species, which indicates that *csd* and *fem* might function differently in each lineage.

ISO-seq data significantly improves the gene annotation

Transcriptome data allows us to identify all expressed genes and provide important evidence for gene annotation. Currently, most of the published genomes were annotated based on RNA-seq data by either mapping the short reads or the pre-assembled transcripts with short reads onto the reference genomes [37]. The single molecular long reads sequencing produces full-length transcript of up to 10 kb can readily be used for gene prediction without the need of assembly, therefore, in principle, it can significantly improve the gene annotation. To provide an insight on how the gene annotation can be improved with the long-reads ISO-seq,

we sequenced the total RNAs from the whole body of worker, gyne, queen, and male of *M. pharaonis* in two sequencing platforms, PacBio SMRT for long reads and BGI-seq for short reads. And we compared the performance of these two datasets in gene prediction and isoform annotation. In total, we obtained 62 Gb long reads transcriptome data (Supplementary Table S7) and 236 Gb RNA-seq data (Supplementary Table S8).

We then generated two versions of annotation for *M. pharaonis* using ISO-seq data and RNA-seq data separately, and compared the gene model prediction, alternative splicing events, the annotations of UTRs, and completeness of predicted genes (Supplementary Table S9). The ISO-seq annotation version identified 186,499 transcripts on 10,626 protein-coding gene loci, with an average of 5.37 exons per transcript. The ISO-seq annotation has improved the gene prediction in many ways. First, the untranslated regions of 10,004 genes were missed in RNA-seq annotation but are annotated in ISO-seq version (Fig. 3A). Second, RNA-seq annotation has missed at least one exon in 2,093 genes but can be re-found in ISO-seq annotation (Fig. 3B). Third, the ISO-seq annotation has also corrected the models of 58 genes that have been falsely annotated into multiple genes (Fig. 3C), while 99 genes were mistakenly merged with neighbor genes (Fig. 3D). Besides, although the high depth RNA-seq data should in principle provide single-base resolution of transcriptome profiling, we found 279 genes in ISO-seq annotation version has been completely missing in the annotation with RNA-seq data. Among them, more than 18% are high GC-content genes and 38% have longer than 200bp repeated sequences, which further demonstrated that the PacBio sequencing is good for the sequencing of high GC-content genes and repeated sequences. In total, 15.86% of genes have been re-fined the coding area with the ISO-seq data, demonstrating the power of long reads sequencing in the aid of gene annotation.

Alternative splicing (AS) landscape of *M. pharaonis*

To identify the AS transcripts, we first cluster all high-quality long reads into final polished isoforms. Over 97.95% of the consensus transcripts can be mapped to the reference genome using GMAP [38] (Supplementary Table S10), indicating again the high completeness of the reference genome. We next collapsed redundant isoforms into 186,499 isoforms, which covering 11,499 genes expressed at least in one caste. The splice junctions (SJs) were detected according to the two pairs of dinucleotides presented at the beginning and end of the introns encompassed by the junctions. The SJs are dominated by canonical GT-AG form, which accounts for more than 94.72% of the total SJs. Over 99% of the SJs with GT-AG form that identified from the ISO-seq were also supported by the RNA-seq data (Supplementary Table S11). These evidences suggest a high accuracy rate of exon-intron boundary structure in our detection with the long-read data and strongly support the validity of the alternative spliced isoforms detection.

A practical question in transcriptome sequencing is that at what sequencing depth the data can provide sufficient enough signals for alternative splicing event detection and comparison. The high coverage ISO-seq data that we generated here allows us to address this question by performing saturation analyses with subtractive samples. We evaluate the impacts of sequencing data amount to the number of consensus transcript, the size of detectable expressed regions, total number of detected expressed genes, genes with AS events, isoforms, and AS events (Fig. 4A). The saturation analysis revealed that the number of consensus transcript, the size of expressed regions and total number of isoforms are constantly increased

with the amount of the raw sequencing data. By mapping all RNA-seq reads onto *M. pharaonis* genome, we estimated that 140.22 Mb genomic regions can be transcribed at least in one caste. With 59 Gb of raw ISO-seq long transcript reads, the total size of detected expressed region still does not reach the saturation phase and spans about 129 Mb covering 92% of potential transcribed regions detected by RNA-seq. This indicates that a large proportion of genomic regions that might actively transcript but has been largely ignored in previous RNA-seq studies. However, we found that the number of detected transcribed genes and the AS events started reaching saturation phase with 10~15 Gb sequencing data, which can detect 9,656 expressed genes, covering at least 93.54% of genes with RNA-seq transcription evidence (an example was shown in Supplementary Fig. S2). These results suggest that our sequencing data from each caste (about 23.0 Gb, 14.0 Gb, 15.3 Gb, 10.4Gb for worker, gyne, queen, and male samples, respectively) is sufficient for covering majority of the expressed genes and the AS events in each caste.

To obtain an overall pattern of AS in *M. pharaonis*, all ISO-seq data were pooled together for AS event and gene isoform detection. Our results show that over 87% of expressed genes have at least two isoforms and on average each gene expresses 9 isoforms in all castes, indicating the complexity nature of the ant transcriptome. Of note, 654 genes have more than 50 isoforms. The most extreme case is mitochondrial NADH-ubiquinone oxidoreductase gene, which has been transcribed into 894 isoforms. Of note, these high isoform richness genes are enriched in transduction (GO:0007165), cell communication (GO:0007154), and regulation of biological process (GO:0050789). Similar with what has been reported in human and many other eukaryotic species, the most dominant AS form is intron retention which consists of

48.77% of all AS events. This ratio is also consistent across caste samples (Fig. 4B, Supplementary Table S12).

Characterization of caste-specific alternative splicing isoforms

Alternative splicing (AS) is an important mechanism in defining tissue specificity through expressing the tissue-specific transcript of the same gene. Previous studies showed that AS was associated with phenotypic variation in eusocial insects, where a single genome is able to encode for numerous caste phenotypes [12, 14, 16, 17, 39]. Thus, we investigated the isoform specificity and commonality among four castes. Among all expressed genes, we found 5,359 genes transcribe at least one caste-specific isoform that only presents in one caste, which suggests that the AS has pervasive impacts on genome-wide protein-coding genes with diverse functions that might contribute to the caste differentiation. By performing the KEGG analysis, we found many genes with caste-specific isoforms related to the insulin pathway and mTOR signaling pathway which play key roles in regulating the caste differentiation on morphologies and longevity [13, 40, 41] (Supplementary Table S13-16).

To further characterize the caste-specific alternative splicing isoforms, we highlighted some functionally important genes that may play important roles in ant sex determination and caste differentiation. *Feminizer* (*fem*), functions as a binary switch gene participating in the sex determination and sexual differentiation in Hymenoptera [35, 36, 42]. In pharaoh ant, *fem* includes eight coding exons and the full length transcripts with all coding exons were only expressed in the female castes (Supplementary Fig. S3), while the male caste only expresses the first two coding exons, which indicates that they have different function according to their

difference on the protein domains. The sex-different expression AS pattern of *fem* seems to be conserved as *transformer* gene across different insects [42]. Moreover, we found that the female castes express diverse transcript isoforms of this gene with many isoforms which might function as lncRNAs.

By choosing the highest expressed isoform for each castes of pharaoh ant, we screened out the genes with dominant AS isoforms for each caste, and selected 267 genes with caste-specific dominant AS isoforms (supplementary Table S17). We reason that these genes might be potential candidate genes involved in ant caste differentiation via alternative splicing. The *cytokine receptor-like factor 3* (*crlf3*) was identified as a neuroprotective erythropoietin receptor in beetle *Tribolium castaneum* and *Locusta migratoria* neurons, and emerged with the evolution of the eumetazoan nervous system [43, 44]. It mediates the neuroprotective effects of erythropoietin without erythropoiesis, thus playing a crucial role in nerve cell survival [44]. In pharaoh ant, *crlf3* have several caste-specific isoforms and also different dominant expressed isoform in each caste (supplementary Fig. S4). The worker-specific isoform of this gene shows the highest expression level in workers. And queens also mainly expressed its caste-specific isoform. Considering the key role of nerve system in caste differentiation, it further indicates that the caste-specific dominant AS isoform in *crlf3* may influence the caste differentiation in pharaoh ant.

Identification and comparative analysis of long noncoding RNAs

Long noncoding RNAs (lncRNAs) are a group of RNA molecules (>200 nt) that are not translated into proteins, but play very important roles in a variety of biological processes [45].

The detection power of lncRNA has been restricted in using short-read RNA sequencing technology, since the short reads sequencing is failed to completely capture the full length of extremely long ncRNAs. Therefore, the number of previous detected lncRNA might be underestimated, which can be improved by the full-length isoform sequencing. Taking advance of the full length transcripts from ISO-seq, we detected 1,225 long transcripts with each has at least 2 full length transcripts supported that do not have protein coding frame and likely function as lncRNAs (See Methods). The length of these lncRNA varies from 923 to 30,849 bp, which is far longer than that of the lncRNAs predicts in other ant species, *C. floridanus*, *H. saltator*, and *O. biroi* by the RNA-seq (Fig. 5A) [21, 46]. Based on the relative position to the annotated genome, pharaoh ant lncRNAs can be classified into four categories: antisense, overlapping with coding sequences, intronic, and intergenic (Fig. 5B) [47]. Most of the lncRNAs are located in the intergenic region (64.33%) as observed in other organisms, and they probably function as transcription regulators [48, 49].

The number of lncRNAs in *M. pharaonis* varied among castes. Gyne has the highest number of lncRNAs while male has the smallest number (Table 4). Queen has the longest lncRNA among the four castes, with the average length 5,675 bp, while male has the shortest among four castes, with the average 3,456 bp (Table 4). Worker expresses the highest level of lncRNAs, while queen has the lowest expression (Supplementary Fig. S4).

**Sample**	**# LncRNAs**	**length(bp)**			**# of Caste-specific expressed lncRNAs**
		**Min**	**Max**	**Average**	
Worker	531	942	25,018	4,438	30

Gyne	543	982	19,746	4,648	20
Queen	360	1,344	30,849	5,675	4
Male	149	923	12,182	3,456	27

Table 4. Statistics of the predicted lncRNAs in four castes

Although investigating how lncRNA works is challenging because of their weak expression nature, cell/tissue-specificity, and capricious functions, some lncRNAs preserve high conservation level on either sequence or secondary structure cross species, which provides a way to detect evolutionary signals for functional importance. By genomic comparison across 4 ant genomes sequenced with long reads, we identified genomic regions that are under extremely low mutation rate and are highly conserved across all detected species. We found 961 (78%) lncRNAs contained at least one genomic element that is highly conserved across all ant species and might have under strong purifying selection during the ant evolution.

We further quantify the expression level of these conserved lncRNAs in ant brains using the brain RNA-seq data and to identify caste-specifically expressed conserved lncRNAs when expression level (TPM, transcripts per million) of that lncRNA is higher than 5 and have more than 1.5 fold differences between castes. By doing so, we found 81 caste-specifically expressed lncRNAs in *M. pharaonis* (Table 4, Fig. 5C). Among them, we found some of the lncRNAs expressed among all castes of *M. pharaonis*. For example, the single exon lncRNA MPWPB.4772.1, which is located between *retn* (*resistin*) and *Obp69a* (*Odorant-binding protein 69a*), was expressed in the four pharaoh ant castes and is highly expressed in worker (Fig. 5D). Since *retn* is highly expressed in worker, while *Obp69a* is highly expressed in male, and *Obp69a* was demonstrated to play important roles in the fly social interaction [50], therefore this further indicated that this lncRNA might be involved in the worker caste

differentiation and the social behavior in ants.

Discussion

Our study provided a high-quality chromosome level ant genome assembly and the full-length transcriptome data for all the four castes of an ant species. Our newly assembly for pharaoh ant genome has greatly improved the quality in many ways than the previously assembly by the short sequencing reads [24]. Our comparison demonstrated the importance of using long sequencing reads to cover the genomic assembly for both repeat regions and high GC-content genomic regions, particularly for the later which often span genomic elements with regulatory functions such as promoters. By combination of PacBio assembly and the Hi-C data, our study presented an efficient way to produce a chromosome level assembly for ant genome and has been adapted now as a standard genomic sequencing and assembly pipeline for the Global Ant Genomic Alliance, which aims to generate high quality assembly for ca. 200 ant species representing broad diversity of ant species [23]. Further our full-length isoform sequencing has produced high quality genome annotation for *M. pharaonis* and also highlighted the complexity and diversity of ant transcriptome that associated with caste differentiation. Our study has identified many protein coding genes with caste-specific isoforms and a core set of lncRNAs that might have a conserved role in ant caste differentiation along the long evolutionary process of ants. These dataset will be valuable for the further downstream functional study to reveal the genetic mechanisms underlying caste differentiation in ants.

Methods

Sample collection

Monomorium pharaonis were collected from a house of a resident in Mengla, Xishuangbanla district, Yunnan province, China. The nest was brought back to the lab and reared at 27℃, RH 65%, L: D = 12: 12 (with light period 08:00~20:00, dark period 20:00~08:00) for the following studies. Queens and workers used in this study were from the original nest. Gyne and male samples were obtained from a newly developed nest which was isolated from the original nest with only eggs, larvae and workers. For DNA and RNA sequencing, ants were collected and flash frozen in liquid nitrogen, and stored at -80℃ for the later extraction. The collection procedures were in accordance with protocols approved by the Animal Care and Use Committee of Kunming Institute of Zoology.

DNA and RNA extraction

Genomic DNA from pools of worker samples was extracted via an insect SDS DNA extraction protocol provided by Novogene Corporation (Nanjing, China). Total RNAs of different castes (male, worker, gyne and queen) for PacBio full-length ISO-seq were extracted via the Trizol extraction kit according to the manufacturer's instruction. Brains of the males were dissected in a cold DEPC-treated PBS. Five replicates of pooled male brains (n = 20 males/pool) were extracted by the RNA Trizol extraction kit. DNA and RNA quality were checked by Qubit (Life Technologies). DNA and RNA integrity were examined by agarose gel electrophoresis.

PacBio ISO-Seq library construction and sequencing

The full-length ISO-seq libraries were constructed using the total RNAs. First-strand cDNA was synthesized using a ClontechSMARTer PCR cDNA Synthesis Kit with anchored oligo $(dT)_{30}$ as the primer. Double-strand cDNA was generated by large-scale PCR using an optimized PCR cycle number. Separation of different cDNA fractions by length were generated with a BluePippin Size Selection System. Once double-stranded cDNA is prepared, the SMRTbell^{TM} libraries were constructed with Pacific Biosciences SMRTbell Template Prep Kit 1.0 following the vendor's protocol. Three SMRT RNA libraries, 1-2k, 2-3k, and 3-6k, were prepared for worker and gyne samples. Mixed library without doing size-selection were prepared for queen and male samples, as the protocol improved. The SMRTbell libraries were then sequenced on the PacBio Sequel platform.

RNA library construction and sequencing

In parallel, RNA sequencing (RNA-seq) of the male brains was done by constructing a Micro-Tn5 Transposon Library followed the methods described in Zhu et al (2018)[51] and sequenced on an BGISEQ-500 PE100 platform. RNA-seq data from worker, gyne and queen were requested from Qiu et al (2018)[52].

Genome sequencing

To achieve a high-quality pharaoh ant genome assembly, we adopted a combination of sequencing methods including Illumina and PacBio sequencing.

For Illumina sequencing, three short-insert size DNA libraries (250, 500, and 800bp) were constructed using Illumina TruSeq Nano DNA LibraryPrep Kit following the manufacturer’s instructions, and then sequenced on an Illumina Hiseq 2000 instrument using a whole-genome shotgun sequencing (WGS) strategy at BGI-Shenzhen (Shenzhen, China) and obtained a total

of 33 Gb clean data with approximately 103-fold sequence depth.

For PacBio sequencing, the BluePippin Size-selection System was used to perform size selection. In total, DNA was sheared to ~20kb targeted size using ultrasonication (Covais, Woburn, Massachusetts, USA), and finally DNA fragment with 20kb size was retained to construct the libraries. The constructed libraries were sequenced by PacBio Sequel system in Novogene (Tianjing, China), and a total of 12 SMRT cells were used to yield 31Gb sequencing subreads with an average length of 7.5kb and an N50 of 11.6kb.

Genome assembly

Genome size estimation

We estimated the genome size of pharaoh ant using kmer frequency analysis with a kmer size of 17. The genome size was estimated according to the formula: Genome size = # Kmers / Peak of depth.

Genome assembly methods by PacBio long reads

We used an in-house pipeline to perform genome assembly, which included five steps:

(1) Contig construction

Canu (version 1.5) was used to perform the 96-fold PacBio Sequel reads assembly with the default parameters and the complete Canu pipeline. The Contig N50 of the Canu assembly is 1.26Mb and the total assembly size is 323Mb.

(2) Linking contigs to Scaffold

Scaffolding was performed utilizing the SSPACE long read scaffolder. SSPACE-LongRead employs the BLASR aligner, which is used to align the long read set to the Canu contig

assembly. We improved assembly contiguity and acquired larger scaffold N50 than Canu contig assembly.

(3) Filling gaps within scaffolds

After scaffolding, PBJelly was used to fill the gaps within scaffold using the PacBio sequences. The running parameter we used is: -minMatch 8 -sdpTupleSize 8 -minPctIdentity 75 -bestn 1 -nCandidates 10 -maxScore -500 -nproc 13 -noSplitSubreads. Most gaps were filled in this step. This results in the final assembly of 325Mb, with scaffold N50 of 3.63Mb, contig N50 of 2.63 Mb and number of undetermined bases (Ns) of 284Kb (0.08% of the total genome assembly). The contig N50 improved about two times than that of Canu contig assembly.

(4) Two rounds polish of genome assembly

Because of the PacBio raw reads contain the high sequencing error. We performed two rounds genome assembly polishing. In the first round, Arrow software was used to map the PacBio sequences to the genome assembly. Then the small indels and substitutions were corrected and consensus sequences were obtained in this step. We performed the second round of polishing using high quality Illumina Paired-end short reads. Firstly, Illumina short reads were mapped to the assembly using BWA, and then Pilon was used to correct the sequences by input BAM alignments and the assembly sequences. The parameters we used is " --changes --vcf --diploid --fix bases --mindepth 8". The results showed that Pilon corrected 14,680 substitutions, 46,245 small insertions and 9,410 small deletions for raw PacBio reads genome assembly. In this step, the corrected sequences result is the final genome assembly.

In situ Hi-C (chromosome conformation capture) library preparation and chromosome

assembly using Hi-C data

To construct the reference genome at the chromosome level, larvae tissue of pharaoh ant was used to construct a Hi-C library. The library was sequenced on BGISEQ-500 platform with 100 paired-end mode. We used HiC-Pro to filter out invalid reads pairs, such as self-ligation, nonligation, start-nearRsite, PCR amplification, random break, largeSmallFragments and ExtremeFragments. The valid read pairs were mapped to the polished pharaoh ant genome assembly. The contact count between contigs was calculated and normalized by restriction sites in sequences. We successfully produced 11 chromosomes that occupied 94% of the genome using 3D-DNA pipeline. The 11 chromosomes were consistent with the previous karyotype analyses of pharaoh ant.

Genome assembly evaluation

To assess the whole genome assembly base quality, we firstly aligned the high-quality Illumina short reads to the final base error corrected assembly. The percentage of total mapped reads was 97%. Then we used the variant detector FreeBayes to calculate the homozygous variants ratio by inputting the BWA alignments. We detect the Homozygous variants with parameters " -C 2 -0 -O -q 20 -z 0.10 -E 0 -X -u -p 2 -F 0.6 ". The homozygous variations were derived from base calling errors as the genome is diploid. The error rate was calculated as 0.001%, indicates a base quality value at QV50. Quality value and identity are calculated by the algorithm -10 $\log_{10}$(length of variants/#bases>=3Xcoverage) and 100*(1 - length of variants/#bases >= 3Xcoverage). For protein-coding genes regions, we ran BUSCO on genome mode to search for conserved genes in hymenoptera species.

Genome annotation

Annotation of repeat DNA sequences

(1) Identification of known transposable elements (TEs)

We firstly identified know TEs in the pharaoh ant genome using RepeatMasker (http://www.repeatmasker.org/) by searching against the Repbase (version 20.04) TE library. Then, we used RepeatProteinMask (http://www.repeatmasker.org/) within the RepeatMasker package to search the TE protein database.

(2) *De novo* repeat prediction

A *de novo* repeat library using RepeatModeler (version open-1.0.8) was generated first, and then transposable elements were annotated by RepeatMasker using the *de novo* repeat library.

(3) Tandem repeats

We also predicted tandem repeats using TRF, with parameter "Math=2, Mismatch=7, Delta=7, PM=80, PI=10, Minscore=50, and MaxPeriod=12".

Protein-coding gene prediction and functional annotation

A combined Homology-based gene prediction, *de novo* gene prediction, and transcriptome-RNA-seq-based gene prediction were used to annotate the protein-coding sequences in the pharaoh ant genome as we used in the leopard gecko [53]. The reference gene sets of *Drosophila melanogaster*, *Apis mellifera*, *Linepithema humile*, *Nasonia vitripennis*, *Solenopsis invicta*, and *Monomorium pharaonis* from the Ensembl and NCBI databases were used in the homolog-based method. RNA-seq data from the brains of different castes and other tissues downloaded from the NCBI database (NCBI accession number DRR032044 ~ DRR032266) were used in the transcriptome-RNA-seq-based method.

After that, we built a non-redundant (nr) reference gene sets, based on a priority order of

homology-based evidence > transcriptome-based evidence > de novo-based evidence to combine gene evidences. Finally, a total of 15,945 non-redundant protein-coding genes were annotated in pharaoh ant genome.

ISO-seq isoforms improve gene model prediction

Full-length isoform sequencing (ISO-seq) has been demonstrated to improve gene annotations in eukayotic genomes. We incorporated the ISO-seq data to improve the gene model in pharaoh ant genome. Firstly, we compared the location of PacBio isoforms with the reference gene location using gffcompare. The overlapped PacBio isoforms at the same strand with the reference gene loci were used to refine the gene models, introduce alternative splicing events, and update the annotations of UTRs. Besides, we could modify incorrect gene models which caused by incorrect gene prediction. To further investigate the missing and incomplete protein-coding gene model, a Markov model was estimated with 1000 high-quality genes, using trainGlimmerHMM tool included in the GlimmerHMM software package. The putative protein-coding sequence of each PacBio isoform was identified using the Markov model. By comparing the gene model to the reference genome, we generated 15,945 protein-coding genes.

Gene function annotation

Functional annotation of protein-coding genes was done by searching against the function databases COG, TrEMBL, SwissProt, and KEGG using BLASTP. InterProScan (v5.16) with seven different models (Profilescan, blastprodom, HmmSmart, HmmPanther, HmmPfam, FPrintScan and Pattern-Scan) were used to annotate the protein domains and motifs.

Full Length Isoform Sequencing (ISO-seq) analysis

Transcriptome analysis pipeline for ISO-seq

We run ISO-seq analysis using SMRT LinkV5.0 (https://www.pacb.com/training/smrt-link-overview/) on the command line via pbsmrtpipe (https://github.com/PacificBiosciences/pbsmrtpipe) to obtain high-quality PacBio isoform dataset. The analysis includes the following four steps:

(1) CCS (Circular Consensus Sequence, CCS) identification

CCS were created from the raw subreads of PacBio sequences by CCS software (version 3.0.0) within pbsmrtpipe package. CCS software takes multiple reads of the same SMRTbell sequence and combines them, employing a statistical model, to produce one high quality consensus sequence.

(2) Classify CCS to full-length reads

CCSs were classified as full-length non-chimeric and non-full length reads. This is done by identifying the 5' and 3' adapters used in the library preparation as well as the poly(A) tail. A read is considered full-length if both primers are detected at the ends with a poly(A) tail signal of at least 12 consecutive 'A's preceding the 3' primer. This step also removed primers and polyA/T tails accordingly.

(3) Cluster sequences by similarity

Isoform-level clustering was performed by employing the Iterative Clustering and Error correction (ICE) algorithm and clustering the classified transcripts sequences using similarity. For each cluster, the consensus transcripts were obtained.

(4) Polish isoforms with error-correction

The error-correction software arrow packaged in the pbsmrtpipe was used to polish the consensus sequences generated from the transcripts cluster step. Arrow mapped PacBio raw reads to get the consensus and variant calls. This outputed polished, high-quality (predicted accuracy >=99%), full-length isoform consensus sequences, and polished low-quality isoform consensus sequences.

(5) Aligned isoforms to reference genomes

We used Genome Mapping and Alignment Program (GMAP) to align the isoform consensus sequences to the genome assembly with parameters " -f samse -n 0 ", then a Python script from the PacBio repository (https://github.com/Magdoll/cDNA_Cupcake/blob/master/cupcake/tofu/collapse_isoforms_by_sam.py) was used to predict transcript structure and to remove redundant transcripts. Each isoform was compared with reference annotation by gffcompare and the isoforms were further classified into eight groups based on their exon structures.

Rarefaction analysis of ISO-seq data

To investigate whether the sequencing depth of those data is sufficient to capture the majority of the transcriptome of interest. We performed rarefaction analysis on all data from four castes sample libraries. We firstly pooled all sequencing data of four caste samples to reach a total of 58.8Gb subreads. Then, we randomly selected 10%, 20%, 30%, …, 100% of the total subreads to perform similar ISO-seq analysis to measure (1) number of consensus transcripts; (2) genome coverage; (3) number of total isoforms; (4) number of detected expressed genes; (5) alternative splicing (AS) events; and (6) detectable genes with AS. All saturation curves were plotted using ggplot2 in R package.

Identification of alternative splicing (AS) events

To verify the PacBio transcript isoforms, we analysed the isoforms in relation to their splice junctions. Splice junctions could be divided into canonical and non-canonical according to the two pairs of dinucleotides present at the beginning and end of the introns encompassed by the junctions. The canonical splice junction (GT-AG) account for ~95 % of all introns of pharaoh ant PacBio isoforms. Besides, we used the RNA-seq data to investigate the consistency of splice junctions between RNA-seq and ISO-seq data. STAR (version 2.4.0) was used to map the RNA-seq data to the reference genome and all splicing junctions were detected.

We used a Python script to detect AS events from PacBio long reads following the method described in Wang et al (2018)[54]. The script uniquely designate all possible splicing patterns as an AS code according to the relative position of the alternative splice sites that are involved in the splicing variation. Five main modes of alternative splicing (intron retention, exon skipping, alternative 3'-acceptor, alternative 5'-donor and alternative position (both 5'-donor and 3' acceptor) were identified. We generated visualization of AS types using SVG implemented in Perl. We then compared the AS type variation among four castes using a custom script.

Discovery of caste-specific alternative splicing (AS) isoform among four caste samples

To investigate differential alternative splicing isoforms from PacBio isoforms among four caste samples, we used the scripts from Cupcake package (https://github.com/Magdoll/cDNA_Cupcake) to chain the isoforms together across four caste samples with default parameters. The isoforms from different caste samples that had the exact match for every exon boundaries were chained together. Caste-specific isoforms were defined

if the isoforms only exist in unique caste sample. The caste-specific isoforms were compared with the alternative splicing isoform dataset and those contained alternative splicing events were defined as the caste-specific AS isoforms.

LncRNA (Long non-coding RNA) identification from PacBio sequences

We identified lncRNAs from PacBio ISO-seq data sets using a customised pipeline comprised of four steps: (1) The PacBio isoforms were aligned to gene models in the pharaoh ant genome. The isoforms that could not be aligned were considered as novel sequences. We extracted the loci of novel sequences which were not overlapped with reference annotation, or overlapped with reference annotation but on the opposite strand. (2) To filter out the coding potential sequences, we used BLAST to screen the sequences for homology with pharaoh ant proteins, and proteins from functional database (Uniprot). (3) The CPC, PLEK and CPAT software were used to discriminate non-coding sequences from protein-coding genes. The sequences were predicted as non-coding by all three software were deemed as candidate lncRNAs. (4) To eliminate possible effects of transcription or splicing noises on the identification of lncRNAs, we filtered out the lncRNAs that supported by less than two full-length PacBio sequencing reads.

Identification and characterization of ant conserved lncRNAs

The conserved lncRNAs within ants were identified by screening the annotated lncRNAs in the highly conserved non-coding elements (CNEs) between ant genomes. The identification method were as follows: (1) We performed pair-wise whole genome alignment using Lastz between the pharaoh ant genome and three published PacBio genomes (*Camponotus floridanus*, *Harpegnathos saltator* and *Ooceraea biroi*) downloaded from NCBI. Then

multiple alignments of four ant genomes were generated using Multiz with pharaoh ant as the reference. (2)We used PhaseCons to estimate the genome conservation index and further identified the highly conserved elements (HCEs). Briefly, we first used phyloFit to estimate an initial neutral phylogenetic model. Then we ran PhastCons twice, with the first time for estimation of conserved and non-conserved models and second time for prediction of conserved elements. Finally, we identified 408,113 ant HCEs, covering 56Mb of the pharaoh ant genome. (3)We filtered out the HCEs that located in the protein-coding regions, which result to 323,193 conserved non-coding elements (CNEs), covering 32Mb of the pharaoh ant genome. (4) Finally, the annotated lncRNAs located in CNEs were considered as ant conserved lncRNAs. Our analysis revealed a total of 961 conserved ant lncRNAs.

Identification of caste-specific lncRNAs among four caste samples by Illumina data

The use of brain tissue Illumina RNA-seq data for four castes samples allowed the identification of caste-specific expressed lncRNAs. Firstly, the ISO-seq transcriptome quantifications were done with the Salmon pipeline using RNA-seq data from four caste brain tissues, respectively. In brief, RNA-Seq data for the four caste brain samples were quasi-mapped to the ISO-seq transcriptome, after which bias-correction options were turned on to account for guanine-cytosine bias and sequence-specific bias. Then we used the expression values as a present/absent classifier. LncRNAs isoforms were classified as caste-specific lncRNAs if the expression value was present in the unique caste sample.

Availability of supporting data

SMRT sequencing data, Illumina HiSeq data, and BGI-seq data generated in this study can be accessed through the Sequence Read Archive (SRA) of the National Center for Biotechnology Information (NCBI) under accession numbers PRJNA634441

Additional files

Supplementary Fig. S1: Frequency distribution of 17-mer analysis. 17-mers are counted from a subset of paired-end reads from 800 bp libraries. The peak depth is 18X. The total number of 17-mers present in this subset is 6,154,945,619. The genome size, estimated by dividing the total number of 17-mer by the peak depth, is 342Mb.

Supplementary Fig. S2: An example show that the full-length PacBio isoform were supported by the short RNA-seq reads.

Supplementary Fig. S3: Sex-specific splicing of *fem* in pharaoh ant. It indicates that the full-length transcript with all coding exons only expressed in the female castes.

Supplementary Fig. S4: Caste-specific isoforms and dominant AS isoforms of *crlf3* in pharaoh ant.

Supplementary Fig. S5: Heat map comparing the overall lncRNA expression.

Supplementary Table S1: Statistics of 17-mer analysis

Supplementary Table S2: The statistics of Illumina and PacBio sequencing data for *M. pharaonis*. Data are produced by short/long insert-size libraries. The sequencing depth was calculated by the assembled genome size.

Supplementary Table S3: PacBio assembly statistics at different stages

Supplementary Table S4: Statistics of the assembled pharaoh ant chromosome

Supplementary Table S5: The 27 ant species for which sequenced genomes are available, in alphabetical order. Modified from Boomsma et al. 2017.

Supplementary Table S6: The status of high GC-content genes in short-reads assembly

Supplementary Table S7: Summary of ISO-seq data from different castes of pharaoh ant

Supplementary Table S8: Summary of RNA-seq data

Supplementary Table S9: Statistic of genes that corrected by the ISO-seq data

Supplementary Table S10: The statistics of consensus transcripts mapping to genome

Supplementary Table S11: Summary of splice junctions among four caste samples

Supplementary Table S12: Summary of Alternative splicing (AS) events in four caste samples

Supplementary Table S13: KEGG analysis of caste-specific isoforms in worker

Supplementary Table S14: KEGG analysis of caste-specific isoforms in gyne

Supplementary Table S15: KEGG analysis of caste-specific isoforms in queen

Supplementary Table S16: KEGG analysis of caste-specific isoforms in male

Supplementary Table S17: Summary of the genes with caste-specific dominant isoform in four caste samples

Abbreviations

AS: Alternative splicing; BLAST: Basic Local Alignment Search Tool; bp: base pairs; BUSCO: Benchmarking Universal Single-Copy Orthologs; BWA: Burrows-Wheeler Aligner; CCS: Circular Consensus Sequence; CDS: coding domain sequence; CNEs: Conserved

Non-coding Elements; COG: Clusters of Orthologous Groups; Gb: gigabase pairs; GC: guanine-cytosine; GMAP: Genomic Mapping and Alignment Program; GO: Gene Ontology; HCEs: Highly Conserved Elements; Hi-C: High-through Chromosome Conformation Capture; ICE: Iterative Clustering and Error correction; ISO-seq: Isoform equencing; kb: kilobase pairs; KEGG: Kyoto Encyclopedia of Genes and Genomes; lncRNA: long non-coding RNA; Mb: megabase pairs; NCBI: National Center for Biotechnology Information; NR: Non-Redundant database; PacBio: Pacific Biosciences; QV: quality value; RNA-seq: RNA sequencing; SJ: splice junction; SMRT: Single Molecule Real Time; TE: transposable element; TPM, transcripts per million; TRF: Tandem Repeats Finder; UTR: untranslated region; WGS: whole-genome shotgun sequencing

Competing Interests

All authors declare that there are no competing interests.

Funding

This work was supported by Lundbeck Foundation (R190-2014-2827), National Natural Science Foundation of China (31970573) to GZ, and Postdoctoral Research Foundation of China (2017M623081), Funding for Postdoctoral Orientation Training in Yunnan province to QG.

Authors' contributions

QG and GZ conceived and designed the study. QG, ZX, and JZ collected the samples, QG extracted the DNA and RNA, ZX performed the overall genome assembly and transcriptome

analysis, RSL prepared the Hi-C library, LZ conducted chromosomal genome assembly, QG and ZX wrote the manuscript. All authors read and wrote part of the manuscript.

Acknowledgments

Thanks to the lab members of Zhang lab for their feedback of our manuscript.

Figure and Table legends

Figure 1 Characterization of *M. pharaonis* genome assembly. **(A)** A photo of pharaoh ant, *Monomorium pharaonis*, colony with four ant castes, queens, gynes, males and workers. **(B)** Heat map of Hi-C interactions among all chromosomes of pharaoh ant. **(C)** Comparison of Scaffold N50s and Contig N50s of 27 Illumina assembled ants and 4 PacBio assembled ants. The blue-filled triangle represents the genomes with PacBio assembly, while the pink-filled circle represents the short-reads assembly. The previous illumina assembly for *Monomorium pharaonis* was marker on the plot. **(D)** Genome collinearity of the short reads assembly and PacBio long-reads assembly shows that the PacBio assembly is of great advantage in covering the high GC-content region and repeat sequences. The blue marked genes are assembled by both sequencing methods, while the red marked genes are the incomplete genes in the short-reads assembly but assembled complete in the PacBio assembly.

Figure 2 Genome collinearity and gene synteny of *M. pharaonis*. **(A)** The genome collinearity of the chromosomal level assembled pharaoh ant and the clonal raider ant *Ooceraea biroi*, showing drastic genome rearrangements occurred during the genome

evolution of the two species. **(B)** The synteny of the flanking region of *fem* and *csd* across 11 ant species by accessing their reference genomes recently produced by Global Ant Genomic Consortium and 2 wasps from NCBI. Indicating that genome rearrangements of the *fem* have occurred at least three times owing to ant genome evolution from the most recent common ancestor. *csd* and *fem* is marked by Red, while other colors represent the neighbor genes around them in our PacBio assembled ants and the ancestor wasp species.

Figure 3 Comparison of RNA-seq and ISO-seq version of gene annotations. **(A)** UTRs were newly annotated in the ISO-seq annotation. **(B)** Genes were annotated incompletely by missing some exons in the RNA-seq annotation. **(C)** One gene was miss-annotated to multiple genes in RNA-seq annotation. **(D)** Two genes were miss-annotated to be a combined gene in the RNA-seq version and is corrected by ISO-seq data. Blue: UTR; Red: CDS; Black line: Intron.

Figure 4 Characterization of *M. pharaonis* isoforms from PacBio ISO-seq in four castes. **(A)** Saturation analysis of PacBio ISO-seq data on consensus transcripts, genome coverage, total number of isoforms, detectable genes, alternative splicing events, and detectable genes with AS. **(B)** Distribution of AS events in four ant castes. AS, alternative splicing.

Figure 5 Characterization of lncRNAs. **(A)** Comparisons of lncRNA length distribution among four species and two sequencing methods. **(B)** Classification of lncRNAs in pharaoh ant. **(C)** Heat map shows the expression profile for the caste specifically expressed lncRNAs in four ant castes. Each row represents one lncRNA, and each column represents one ant caste. The relative lncRNA expression is depicted according to the color scale. Red and purple indicates upregulation and downregulation, respectively. The values of 1.5, 0 and −1.5 are the fold changes depicted in the color spectrum. **(D)** Example of a highly conserved caste-specific

lncRNA expression among four castes.

Tables

Table 1. Summary of the genome features of *M. pharaonis*

Table 2. Statistics of functional annotation of protein-coding genes in pharaoh ant

Table 3. Summary of expressed features in the four caste samples and caste-specific isoforms

Table 4. Statistics of the predicted lncRNAs in four castes

References

1. Libbrecht R, Oxley PR, Kronauer DJ and Keller L. Ant genomics sheds light on the molecular regulation of social organization. Genome Biol. 2013;14 7:212.

2. Hölldobler B and Wilson EO. The superorganism. 2009.

3. Thorne BL. Evolution of eusociality in termites. Annu Rev Ecol Syst. 1997;28:27-54.

4. Schwander T, Lo N, Beekman M, Oldroyd BP and Keller L. Nature versus nurture in social insect caste differentiation. Trends Ecol Evol. 2010;25 5:275-82.

5. Imai H, Urbani CB, Kubota M, Sharma G, Narasimhanna M, Das B, et al. Karyological survey of Indian ants. The Japanese J. Genet. 1984;59 1:1-32.

6. Corona M, Libbrecht R, Wurm Y, Riba-Grognuz O, Studer RA and Keller L. Vitellogenin underwent subfunctionalization to acquire caste and behavioral specific expression in the harvester ant *Pogonomyrmex barbatus*. PLoS Genet. 2013;9 8:e1003730.

7. Ingram KK, Krummey S and LeRoux M. Expression patterns of a circadian clock gene

are associated with age-related polyethism in harvester ants, *Pogonomyrmex occidentalis*. BMC Ecol. 2009;9:7.

8. Ingram KK, Kleeman L and Peteru S. Differential regulation of the foraging gene associated with task behaviors in harvester ants. BMC Ecol. 2011;11:19.

9. Morandin C, Havukainen H, Kulmuni J, Dhaygude K, Trontti K and Helantera H. Not only for egg yolk--functional and evolutionary insights from expression, selection, and structural analyses of *Formica* ant vitellogenins. Mol Biol Evol. 2014;31 8:2181-93.

10. Harrison MC, Hammond RL and Mallon EB. Reproductive workers show queenlike gene expression in an intermediately eusocial insect, the buff-tailed bumble bee Bombus terrestris. Mol Ecol. 2015;24 12:3043-63.

11. Friedman DA and Gordon DM. Ant Genetics: Reproductive physiology, worker morphology, and behavior. Annu Rev Neurosci. 2016;39:41-56.

12. Price J, Harrison M, Hammond R, Adams S, Gutierrez-Marcos J and Mallon E. Alternative splicing associated with phenotypic plasticity in the bumble bee *Bombus terrestris*. Mol Ecol. 2018;27 4:1036-43.

13. Wurm Y, Wang J, Riba-Grognuz O, Corona M, Nygaard S, Hunt BG, et al. The genome of the fire ant *Solenopsis invicta*. Proc Natl Acad Sci U S A. 2011;108 14:5679-84.

14. Foret S, Kucharski R, Pellegrini M, Feng S, Jacobsen SE, Robinson GE, et al. DNA methylation dynamics, metabolic fluxes, gene splicing, and alternative phenotypes in honey bees. Proc Natl Acad Sci U S A. 2012;109 13:4968-73.

15. Li-Byarlay H, Li Y, Stroud H, Feng S, Newman TC, Kaneda M, et al. RNA interference

knockdown of DNA methyl-transferase 3 affects gene alternative splicing in the honey bee. Proc Natl Acad Sci U S A. 2013;110 31:12750-5..

16. Terrapon N, Li C, Robertson HM, Ji L, Meng X, Booth W, et al. Molecular traces of alternative social organization in a termite genome. Nature communications. 2014;5:3636.

17. Bonasio R, Li Q, Lian J, Mutti NS, Jin L, Zhao H, et al. Genome-wide and caste-specific DNA methylomes of the ants *Camponotus floridanus* and *Harpegnathos saltator*. Curr Biol. 2012;22 19:1755-64.

18. Yan H, Bonasio R, Simola DF, Liebig J, Berger SL and Reinberg D. DNA methylation in social insects: how epigenetics can control behavior and longevity. Annu Rev Entomol. 2015;60:435-52.

19. Bonasio R, Tu S and Reinberg D. Molecular signals of epigenetic states. Science. 2010;330 6004:612-6.

20. Simola DF, Wissler L, Donahue G, Waterhouse RM, Helmkampf M, Roux J, et al. Social insect genomes exhibit dramatic evolution in gene composition and regulation while preserving regulatory features linked to sociality. Genome Res. 2013;23 8:1235-47.

21. Shields EJ, Sheng L, Weiner AK, Garcia BA and Bonasio R. High-Quality Genome assemblies reveal long non-coding RNAs expressed in ant brains. Cell Rep. 2018;23 10:3078-90.

22. Madoui MA, Engelen S, Cruaud C, Belser C, Bertrand L, Alberti A, et al. Genome assembly using Nanopore-guided long and error-free DNA reads. BMC Genomics.

2015;16:327.

23. Boomsma JJ, Brady SG, Dunn RR, Gadau J, Heinze J, Keller L, et al. The Global Ant Genomics Alliance (GAGA). 2017.

24. Mikheyev AS and Linksvayer TA. Genes associated with ant social behavior show distinct transcriptional and evolutionary patterns. Elife. 2015;4:e04775. doi:10.7554/eLife.04775.

25. Koren S, Walenz BP, Berlin K, Miller JR, Bergman NH and Phillippy AM. Canu: scalable and accurate long-read assembly via adaptive k-mer weighting and repeat separation. Genome Res. 2017;27 5:722-36.

26. Boetzer M and Pirovano W. SSPACE-LongRead: scaffolding bacterial draft genomes using long read sequence information. BMC Bioinformatics. 2014;15:211.

27. English AC, Richards S, Han Y, Wang M, Vee V, Qu J, et al. Mind the gap: upgrading genomes with Pacific Biosciences RS long-read sequencing technology. PLoS One. 2012;7 11:e47768.

28. Quality Value (QV) Scores. https://www.ucalgary.ca/dnalab/sequencing/services/QV.

29. Lieberman-Aiden E, van Berkum NL, Williams L, Imakaev M, Ragoczy T, Telling A, et al. Comprehensive mapping of long-range interactions reveals folding principles of the human genome. Science. 2009;326 5950:289-93.

30. Durand N, Shamim M, Machol I, Rao SP, Huntley M, Lander E, et al. Juicer Provides a one-click system for analyzing loop-resolution Hi-C experiments. Cell Systems. 2016;3 1:95-8.

31. Servant N, Varoquaux N, Lajoie BR, Viara E, Chen CJ, Vert JP, et al. HiC-Pro: an

optimized and flexible pipeline for Hi-C data processing. Genome Biol. 2015;16 1:259.

32. Dudchenko O, Batra SS, Omer AD, Nyquist SK, Hoeger M, Durand NC, et al. De novo assembly of the *Aedes aegypti* genome using Hi-C yields chromosome-length scaffolds. Science. 2017;356 6333:92.

33. Smith IC and Peacock A. XI.—The cytology of pharaoh's ant, *Monomorium pharaonis* (L.). Proceedings of the Royal Society of Edinburgh, Section B: Biological Sciences. 1957;66 3:235-61.

34. Imai H and Yosida T. Chromosome observations in Japanese ants. 1964.

35. Schmieder S, Colinet D and Poirie M. Tracing back the nascence of a new sex-determination pathway to the ancestor of bees and ants. Nature Communications. 2012;3..

36. Nygaard S, Zhang GJ, Schiott M, Li C, Wurm Y, Hu HF, et al. The genome of the leaf-cutting ant *Acromyrmex echinatior* suggests key adaptations to advanced social life and fungus farming. Genome Res. 2011;21 8:1339-48.

37. Zhao S, Zhang B and Kulski J. Impact of gene annotation on RNA-seq data analysis. Next generation sequencing-advances, applications and challenges. 2015.

38. Wu TD and Watanabe CK. GMAP: a genomic mapping and alignment program for mRNA and EST sequences. Bioinformatics. 2005;21 9:1859-75.

39. Weiner SA and Toth AL. Epigenetics in social insects: a new direction for understanding the evolution of castes. Genetics research international. 2012;2012.

40. Chandra V, Fetter-Pruneda I, Oxley PR, Ritger AL, McKenzie SK, Libbrecht R, et al. Social regulation of insulin signaling and the evolution of eusociality in ants. Science.

2018;361 6400:398-402.

41. Chen X, Hu Y, Zheng H, Cao L, Niu D, Yu D, et al. Transcriptome comparison between honey bee queen-and worker-destined larvae. Insect Biochem Mol Biol. 2012;42 9:665-73.

42. Verhulst EC, van de Zande L and Beukeboom LW. Insect sex determination: it all evolves around transformer. Curr Opin Genet Dev. 2010;20 4:376-83.

43. Hahn N, Knorr DY, Liebig J, Wüstefeld L, Peters K, Büscher M, et al. The insect ortholog of the human orphan cytokine receptor CRLF3 is a neuroprotective erythropoietin receptor. Front Mol Neurosci. 2017;10:223.

44. Hahn N, Buschgens L, Schwedhelm-Domeyer N, Bank S, Geurten BRH, Neugebauer P, et al. The orphan cytokine receptor CRLF3 emerged with the origin of the nervous system and is a neuroprotective erythropoietin receptor in Locusts. Front Mol Neurosci. 2019;12:251.

45. Losko M, Kotlinowski J and Jura J. Long noncoding RNAs in metabolic syndrome related disorders. Mediators Inflamm. 2016;2016:5365209.

46. McKenzie SK and Kronauer DJ. The genomic architecture and molecular evolution of ant odorant receptors. Genome Res. 2018;28 11:1757-65.

47. Derrien T, Johnson R, Bussotti G, Tanzer A, Djebali S, Tilgner H, et al. The GENCODE v7 catalog of human long noncoding RNAs: analysis of their gene structure, evolution, and expression. Genome Res. 2012;22 9:1775-89.

48. Marques AC and Ponting CP. Intergenic lncRNAs and the evolution of gene expression. Curr Opin Genet Dev. 2014;27:48-53.

49. Vance KW and Ponting CP. Transcriptional regulatory functions of nuclear long noncoding RNAs. Trends Genet. 2014;30 8:348-55.

50. Bentzur A, Shmueli A, Omesi L, Ryvkin J, Knapp JM, Parnas M, et al. Odorant binding protein 69a connects social interaction to modulation of social responsiveness in *Drosophila*. Plos Genet. 2018;14 4.

51. Zhu FY, Chen MX, Ye NH, Qiao WM, Bei G, Wai-Ki L, et al. Comparative performance of the BGISEQ-500 and Illumina HiSeq4000 sequencing platforms for transcriptome analysis in plants. Plant Methods. 2018;14 1:69-.

52. Qiu BT, Larsen RS, Chang NC, Wang J, Boomsma JJ and Zhang GJ. Towards reconstructing the ancestral brain gene-network regulating caste differentiation in ants. Nat Ecol Evol. 2018;2 11:1782-91.

53. Xiong Z, Li F, Li Q, Zhou L, Gamble T, Zheng J, et al. Draft genome of the leopard gecko, *Eublepharis macularius*. GigaScience. 2016;5 1:s13742-016-0151-4.

54. Wang MJ, Wang PC, Liang F, Ye ZX, Li JY, Shen C, et al. A global survey of alternative splicing in allopolyploid cotton: landscape, complexity and regulation. New Phytol. 2018;217 1:163-78.

 Click here to download Table Table 3.xlsx

Table 3. Summary of expressed features in the four caste samples and caste-spe[illegible]

Sample	Total isoforms	Total expressed Genes	Genes with multiple isoforms	Average number of isoforms per gene	Isoforms with AS		Caste-[illegible] isof[illegible]
					Number	Percent (%)	Number
**Worker**	75,945	9,202	7,434	5.54	12,691	16.71	31,403
**Gyne**	61,998	8,812	6,685	4.59	9,367	15.11	22,973
**Queen**	72,591	8,766	6,963	5.98	10,734	14.79	30,412
**Male**	45,631	8,614	6,365	3.84	8,397	18.40	17,214

cific isoforms

Specific [illegible]	Caste-Specific isoforms with AS		
Percent (%)	Number	Percent among total isoforms (%)	Percent among caste specific isoforms (%)
41.35	9,121	12.01	29.04
37.05	6,161	9.94	26.82
41.90	7,543	10.39	24.80
37.72	5,245	11.49	30.47

Figure 1

Click here to download Figure Figure 1.pdf

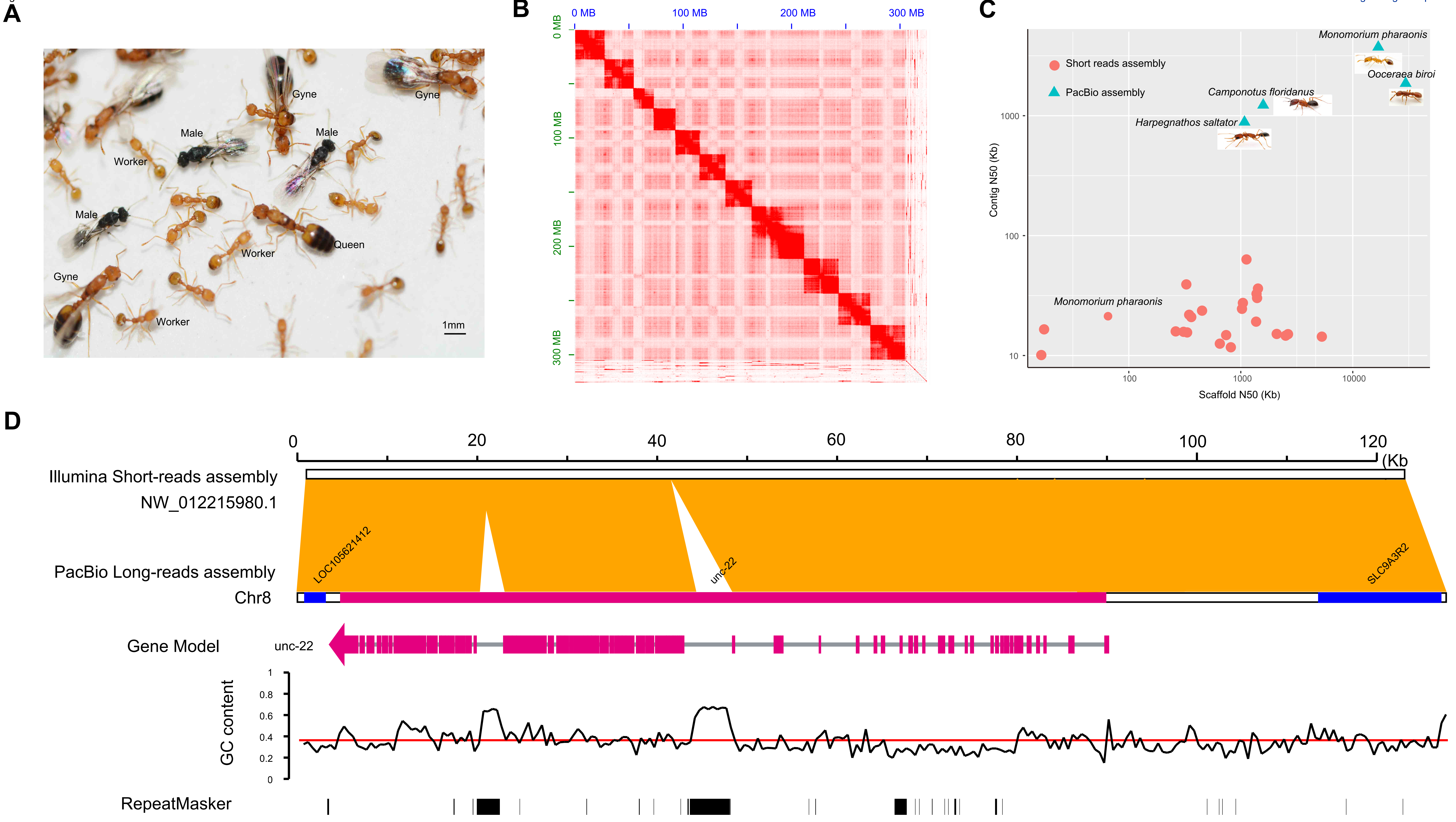

Figure 2

Click here to download Figure Figure 2.pdf

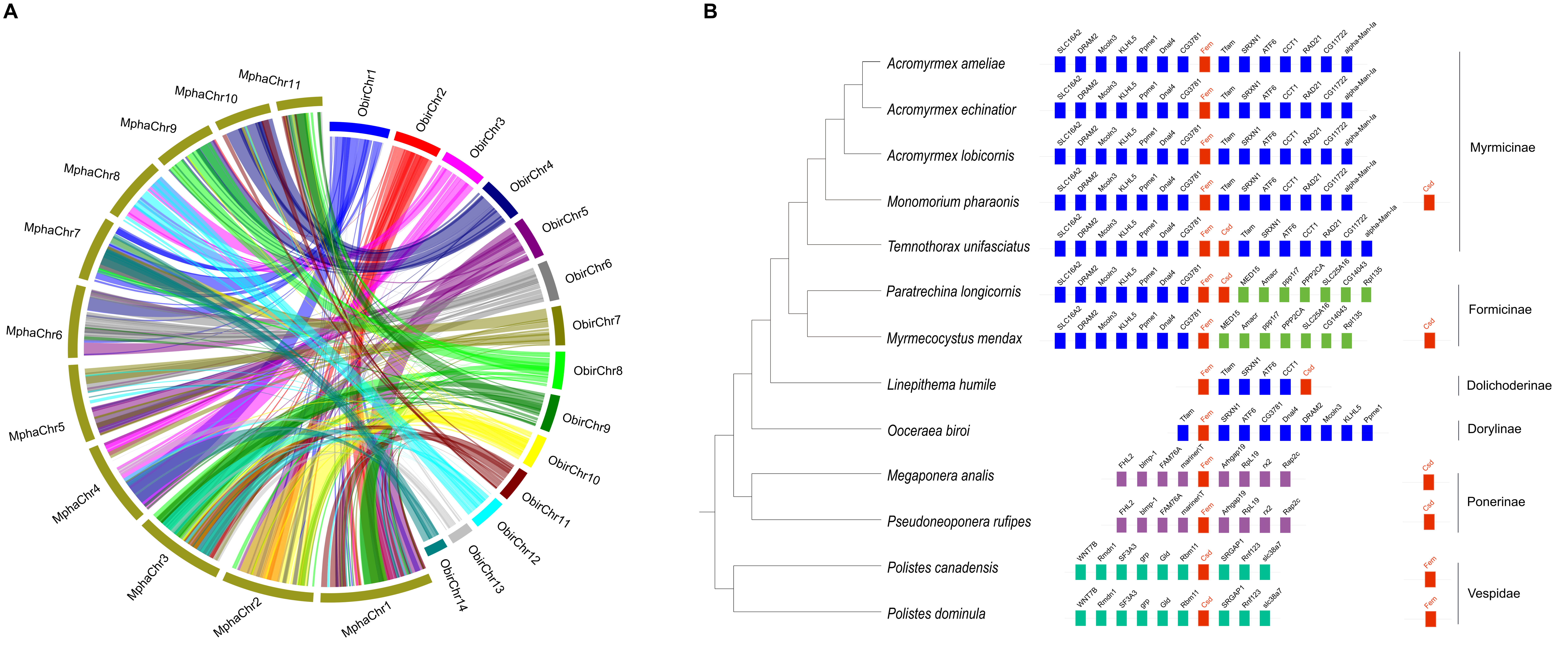

Figure 3

Click here to download Figure Figure 3.pdf

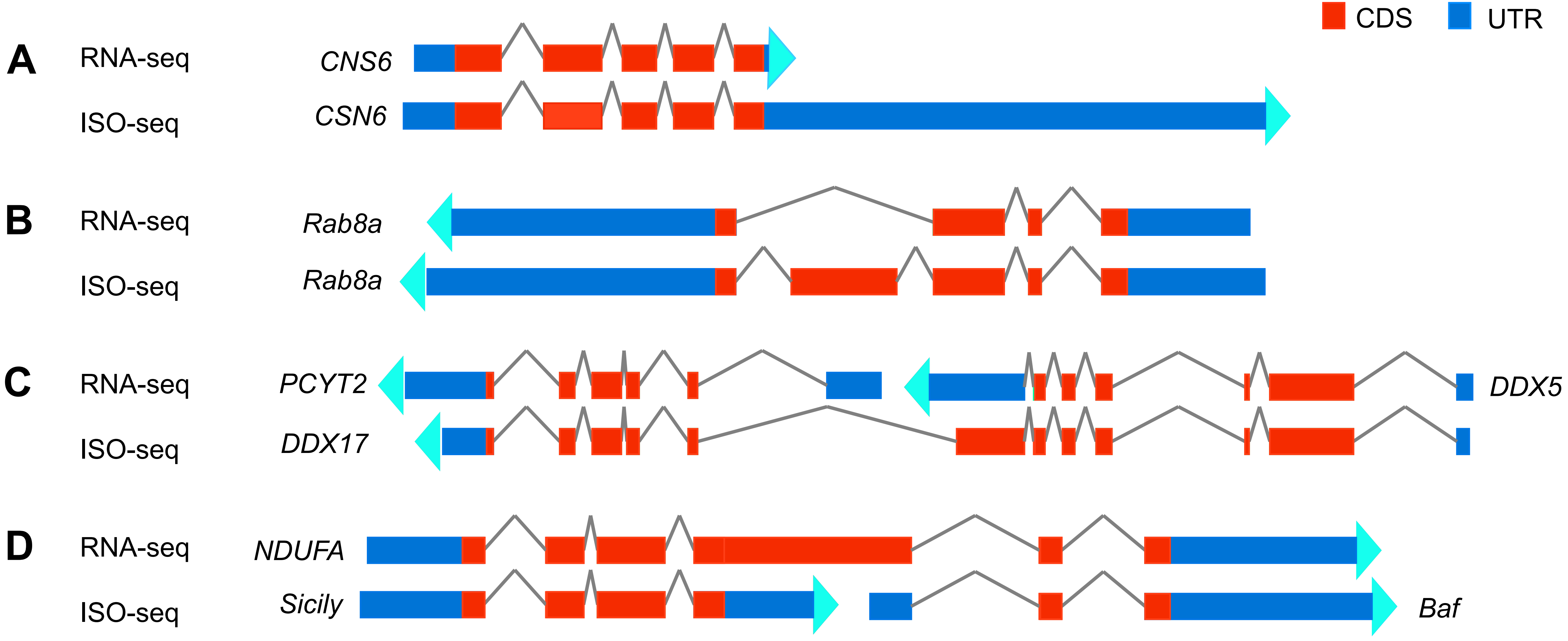

Figure 4

Click here to download Figure Figure 4.pdf

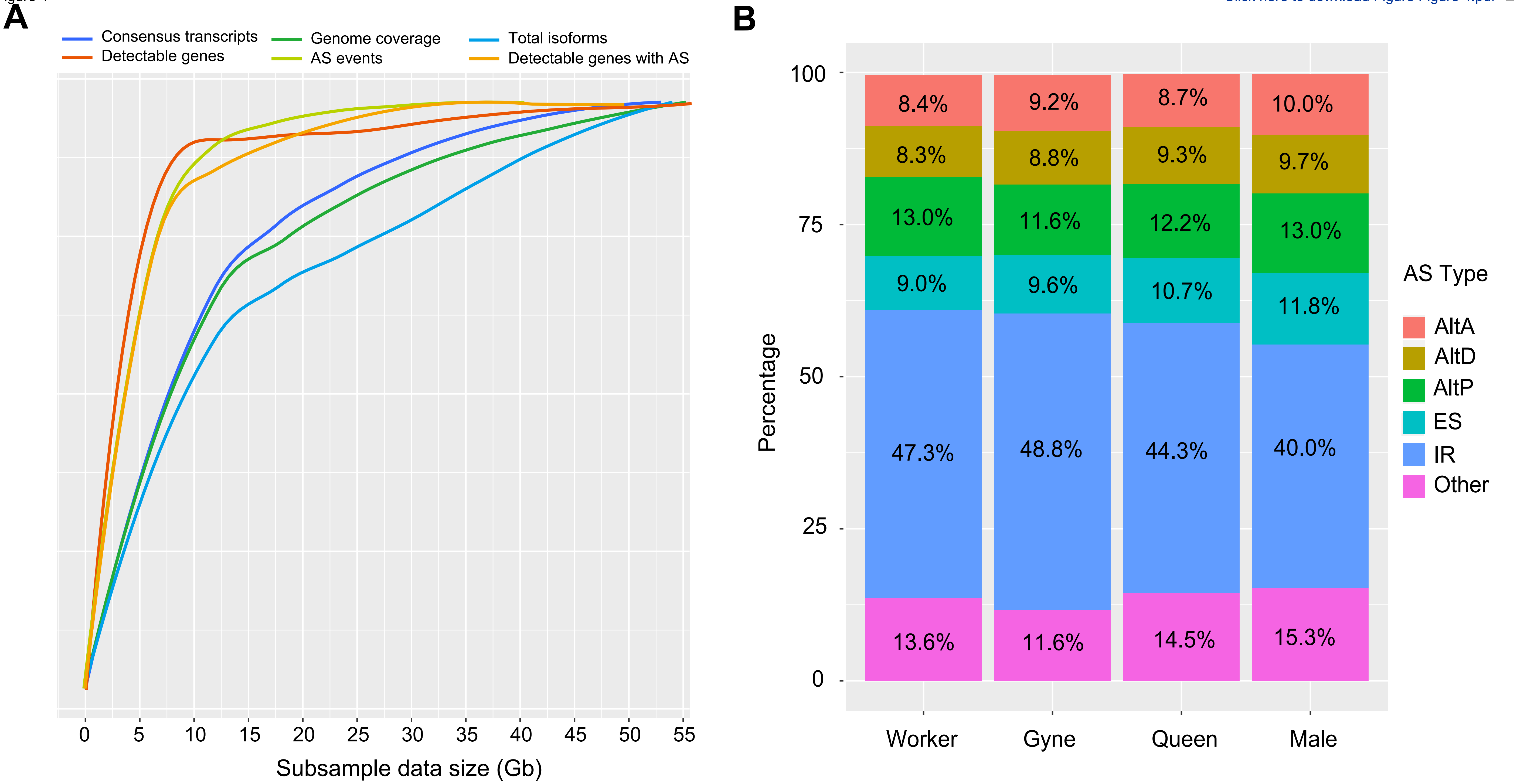

Figure 5

Click here to download Figure Figure 5.pdf

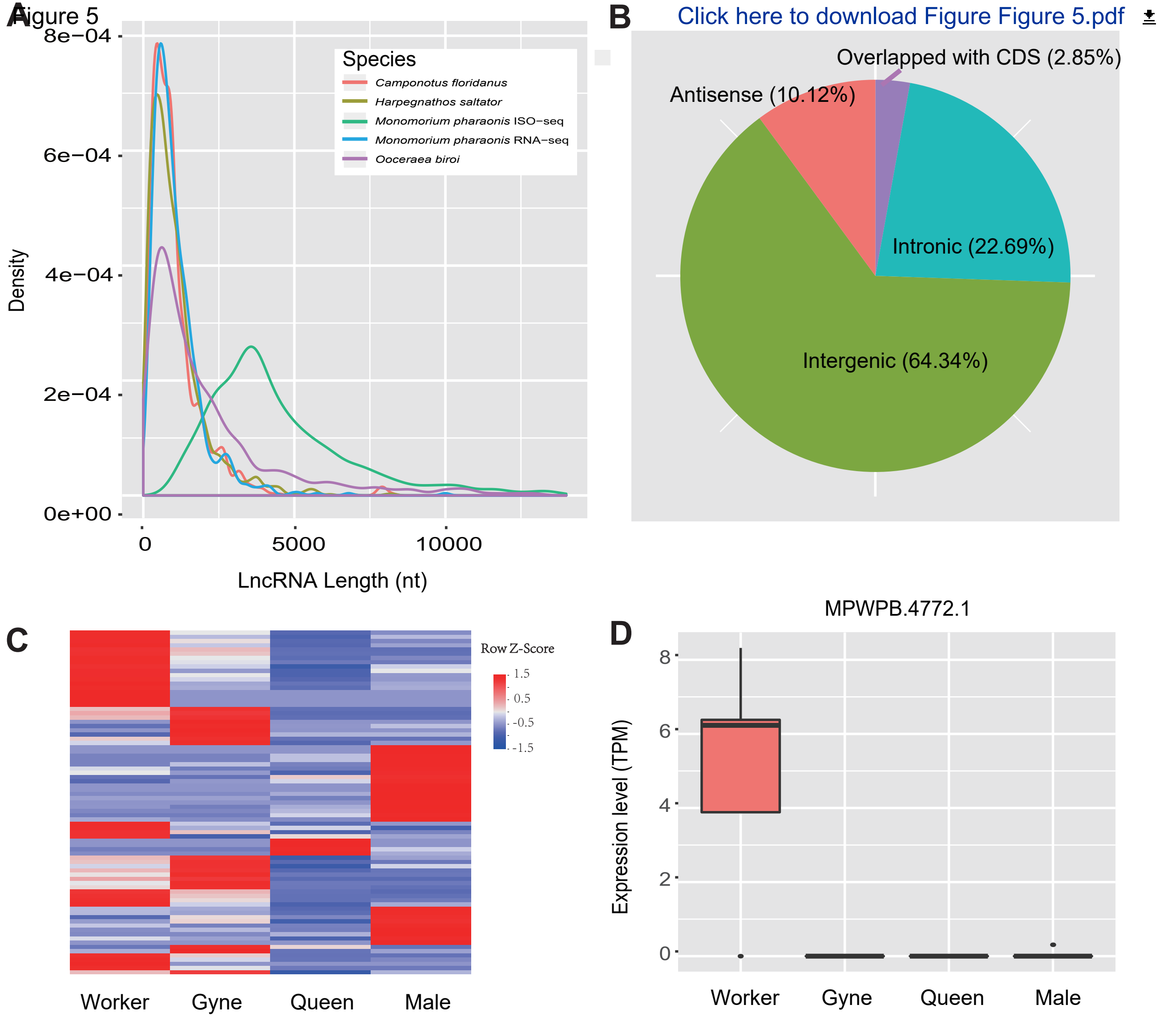

Supplemental Fig.S1

Click here to access/download
Supplementary Material
Figure S1.pdf

Supplemental Fig.S2

Click here to access/download
Supplementary Material
Figure S2.pdf

Supplemental Fig.S3

Click here to access/download
Supplementary Material
Figure S3.pdf

Supplemental Fig.S4

Click here to access/download
Supplementary Material
Figure S4 .pdf

Supplemental Fig.S5

Click here to access/download
Supplementary Material
Figure S5.pdf

Click here to access/download
Supplementary Material
Supplemental table S1-17.xlsx
