# Supplementary figures and images for: High-quality chromosome-level genome assembly and full-length transcriptome analysis of the pharaoh ant Monomorium pharaonis
Source: Gigascience. 2020 Dec 15;9(12):giaa143. doi: 10.1093/gigascience/giaa143 (PMC7736795; doi:10.1093/gigascience/giaa143)

# 17-mer depth distribution

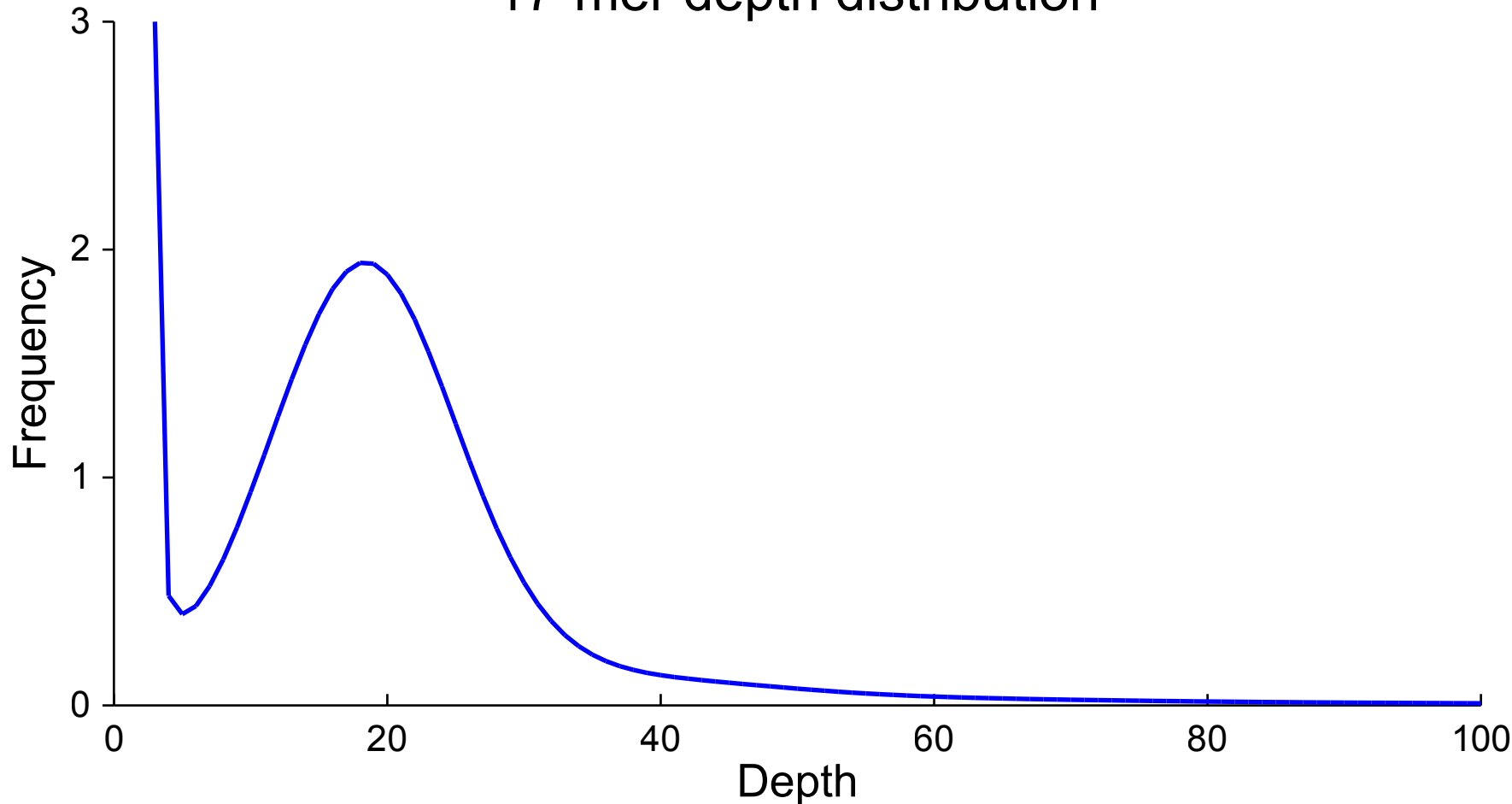

Supplement: giaa143_Supplemental_Figures_and_Tables [file giaa143_supplemental_figures_and_tables.zip › Figure S1.pdf]

5 kb

*Pof*

PacBio isoform

RNA-seq reads

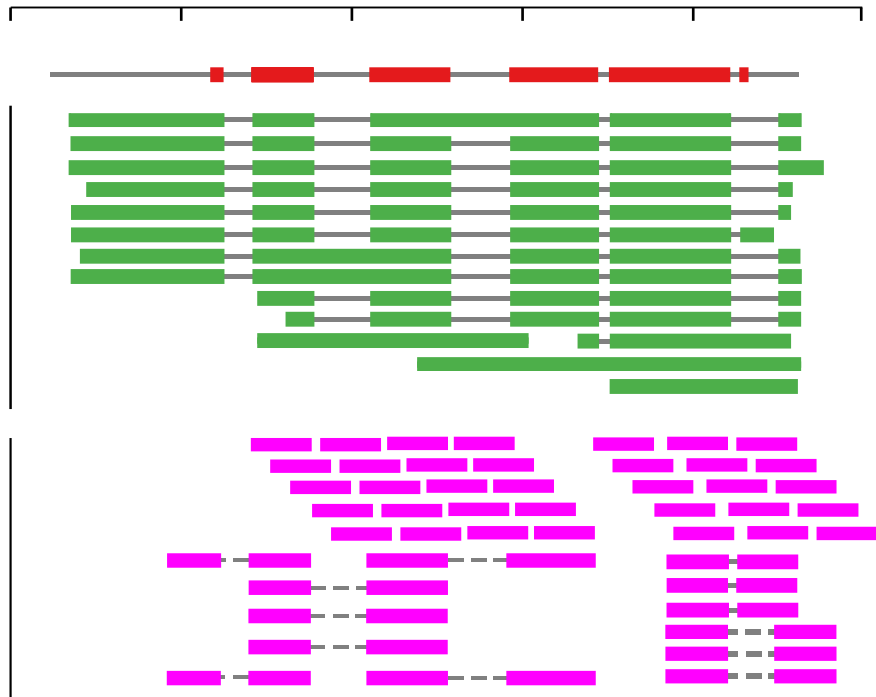

Supplement: giaa143_Supplemental_Figures_and_Tables [file giaa143_supplemental_figures_and_tables.zip › Figure S2.pdf]

Reference gene (fem)

Female

Male

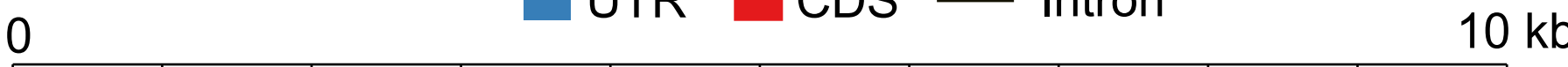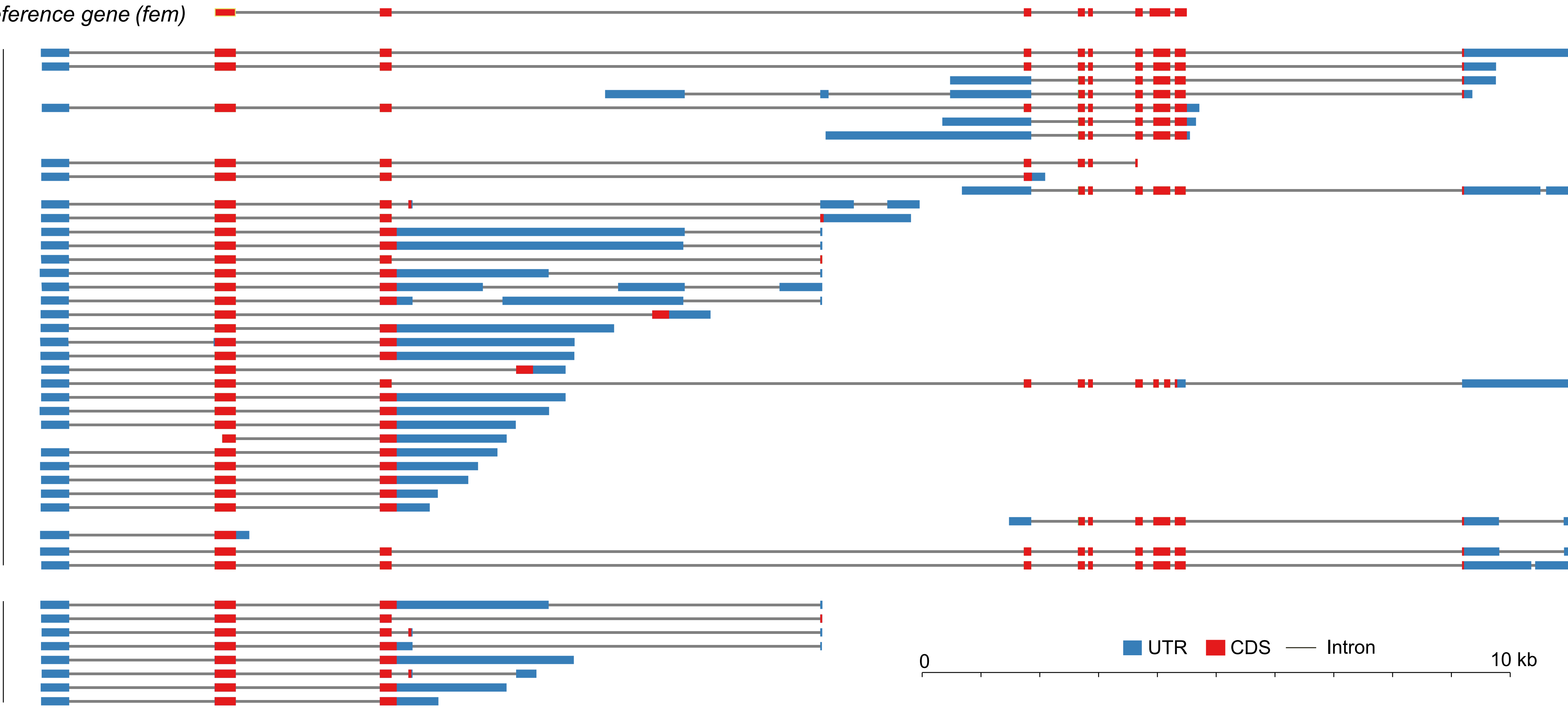

Supplement: giaa143_Supplemental_Figures_and_Tables [file giaa143_supplemental_figures_and_tables.zip › Figure S3.pdf]

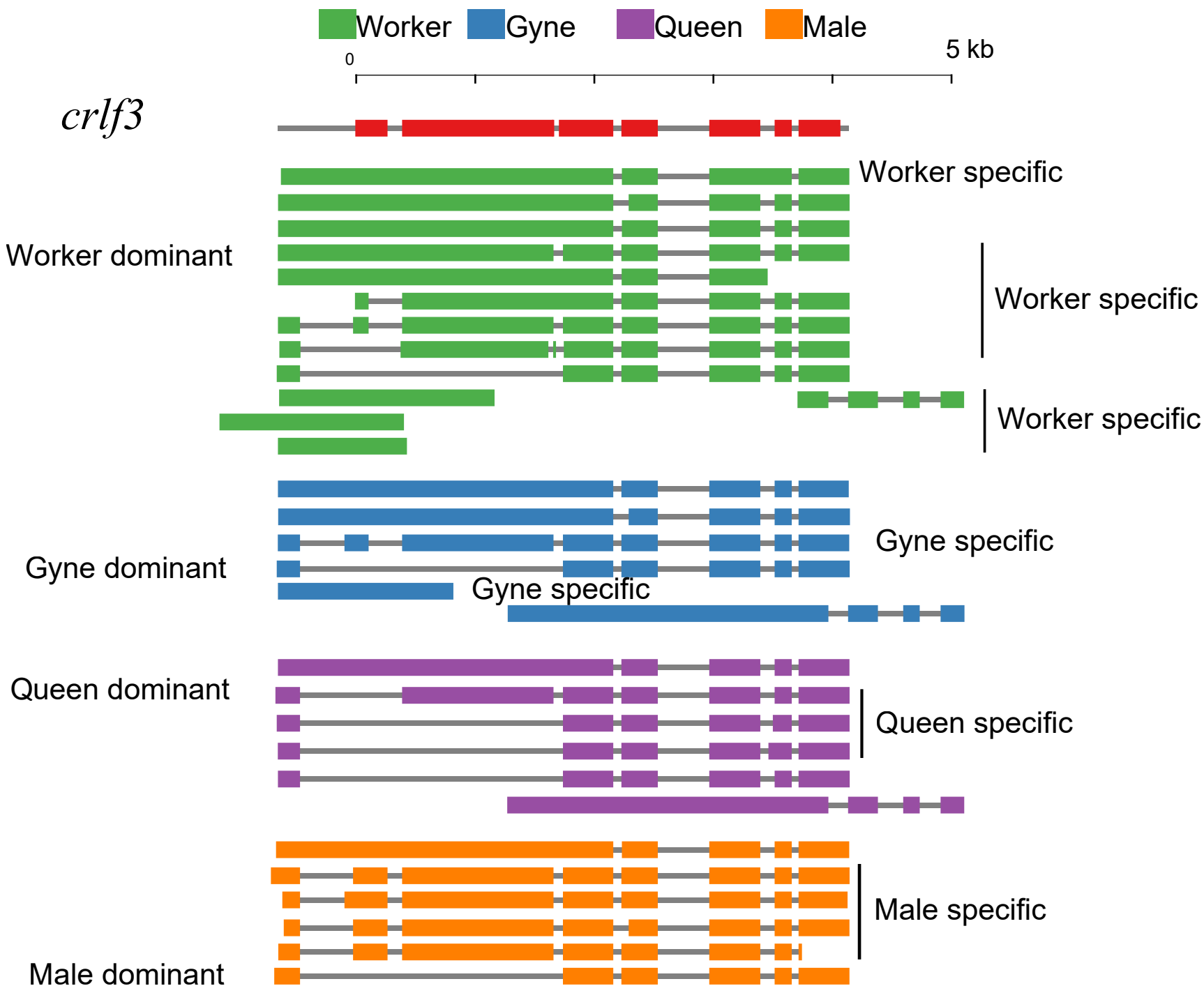

Supplement: giaa143_Supplemental_Figures_and_Tables [file giaa143_supplemental_figures_and_tables.zip › Figure S4 .pdf]
