# Supplementary material for: High-quality chromosome-level genome assembly and full-length transcriptome analysis of the pharaoh ant Monomorium pharaonis
Source: Gigascience. 2020 Dec 15;9(12):giaa143. doi: 10.1093/gigascience/giaa143 (PMC7736795; doi:10.1093/gigascience/giaa143)
Supplement: giaa143_Response_to_Reviewer_Comments_Original_Submission [file giaa143_response_to_reviewer_comments_original_submission.pdf]

Author's Response To Reviewer Comments

Close

Response to Editor and Reviewers' Comments

Editor's comments:

Dear Prof. Zhang,
Your manuscript "A high-quality chromosome-level pharaoh ant genome assembly and full-length transcriptome provide insights on ant caste differentiation" (GIGA-D-20-00148) has been assessed by our reviewers. Although it is of interest, we are unable to consider it for publication in its current form. The reviewers have raised a number of points which we believe would improve the manuscript and would allow a revised version to be published in GigaScience.
Their reports, together with any other comments, are below. Please also take a moment to check our website at https://www.editorialmanager.com/giga/ for any additional comments that were saved as attachments. In particular the reviewers request that the paper gets a more thorough copy-edit, and that some of the biological insights are maybe toned down slightly to focus the paper more on the methodological improvements here. As some of the authors are BGI employees and BGI-tech is being showcased can this also be highlighted in the competing interests section.
Response: Thank you for your suggestions. As requested, we have toned down the statements regarding biological insights in the text. Besides, we have added the following sentence in the competing interests section: "The authors declare that Zijun Xiong, Long Zhou, Guo Ding, and Guojie Zhang are employees of BGI".

Once you are able to fully address these points, we would ask you to submit a revised manuscript to GigaScience. Once you have made the necessary corrections, please submit online at:
https://www.editorialmanager.com/giga/
If you have forgotten your username or password please use the "Send Login Details" link to get your login information. For security reasons, your password will be reset.
Please include a point-by-point within the 'Response to Reviewers' box in the submission system. Please ensure you describe additional experiments that were carried out and include a detailed rebuttal of any criticisms or requested revisions that you disagreed with. Please also ensure that your revised manuscript conforms to the journal style, which can be found in the Instructions for Authors on the journal homepage. If the data and code has been modified in the revision process please be sure to update the public versions of this too.
The due date for submitting the revised version of your article is 30 Sep 2020.
I look forward to receiving your revised manuscript soon.
Best wishes,
Hongling Zhou
GigaScience
www.gigasciencejournal.com

[Reviewer #1's comments]:
Reviewer #1: The manuscript "A high-quality chromosome-level pharaoh ant genome assembly and full-length transcriptome provide insights on ant caste differentiation" contributes to the field of sociogenomics by providing and reporting on an improved version of the genome of the pharaoh ant Monomorium pharaonis. The amount of data provided alongside this manuscript as well as the innovative methods used, fit the aims of this journal well.
In general, this manuscript adds to the growing number of ant genomes published. To assemble genomes at the chromosome level at high quality is essential for follow-up studies to produce reliable results. Thus, this work is of great interest for studies on M. pharaonis as well as for comparative studies across ant or Hymenopteran species. The authors implemented several steps in their pipeline to ensure a sufficient quality of the genome, still some details are missing and should be reported (see Additional comments).
This study clearly demonstrates the importance of long-read sequencing to improve genome quality and gene annotation, and more generally to conduct genomic and transcriptomic studies. While the technical

sections of the study are very strong, the biological aspects are relatively weak. The absence of biological replication in the experimental design and the presence of confounding factors (samples collected in different colonies or comparison across studies with one treatment group in one study, and another treatment group in another study) make it impossible to assess whether the caste-specific patterns reported in this manuscript reflect a biological reality or merely stem from sample-specific noise (that could come from technical and/or biological random variation).
Response: Thank you for your comments and suggestions. While we appreciate the value of adding biological replication for ISO-seq, we have not done so for several reasons. First, there were three major purposes of performing ISO-seq analyses in our current study: 1) to assist the annotation, 2) to identify the alternative splicing forms, and 3) to identify lncRNAs. The main results produced from these analyses were the presence or absence of transcript isoforms, rather than the quantification of transcript expression. The former relies more on ISO-seq depth than biological replication. Thus, we used pooled samples for ISO-seq to produce high-coverage sequencing data to ensure that we discovered lowly expressed isoforms and to mitigate the variation across individuals/colonies. Quantification analyses were performed using RNA-seq data produced in our previous study (Qiu et al., 2018) with biological replications. Second, all samples were collected from sub-colonies developed from the same starting colony to reduce biological variation in our data. We have added this detail in the Methods section at Line 384-396.
We do agree that ideally, biological replication should be conducted. However, this will significantly increase the cost of the project as ISO-seq is still very expensive to produce. We have toned down some of the discussion on the biological findings in the revision.
Qiu B, Larsen RS, Chang NC, Wang J, Boomsma JJ, Zhang G. Towards reconstructing the ancestral brain gene-network regulating caste differentiation in ants. Nature ecology & evolution. 2018;2(11):1782-91.

In our opinion, this study would benefit (and its scope would better fit GigaScience) from toning down (or even removing) the attempt at biological interpretation to focus on its very important finding that long-read sequencing may be a game changer in the field of sociogenomics.
Response: Many thanks. We have toned done the biological interpretations in the text according to your suggestions.

Additional comments

- The manuscript is riddled with typos and grammar errors, and would definitely benefit from being corrected by an English native speaker.
Response: Thank you. The current revision has been polished by a native English speaker.

- Line 162: The results supporting genomic rearrangements between M. pharaonis and O. biroi are interesting, but it is only very superficially discussed from a biological point of view. This is not necessarily problematic, if the point here is to show that long-read sequencing is required to perform such analyses, but then it should be clearly stated and acknowledged.
Response: The reviewer is correct that this is just a showcase to demonstrate that chromosome-level genomic rearrangement can be assessed with chromosome-level genome assemblies. A more detailed finding on the chromosome-level genome assembly requires comparison with more genomes. We thus modified the sentence and toned done the biological interpretation (See current line 163-174).

- Line 165: Is Global Ant Genomic Consortium GAGA? Should it be Alliance instead of Consortium? Are these reference genomes accessible by everyone? Which versions of these genomes were used? Are they published? If not, they should be made accessible together with the paper according to GigaScience guidelines.
Response: We have corrected the misspelling of GAGA. The full genomes of other species have not yet been published. We have uploaded the sequences of this locus for these species in Mendeley Data (DOI: 10.17632/pgxhnytds4.1).

Results:
- Lines 227-229: The number of isoforms is steadily increased with the coverage. What are the implications? Is there the possibility of false positives?
Response: This result implies that many lowly expressed isoforms are hard to capture using ISO-seq. This is because these sequences are lowly represented in total RNAs and have a lower chance of being amplified before sequencing. With higher sequencing, there is an increased chance of discovering these lowly expressed isoforms. The reviewer has raised a good point that some novel isoforms discovered by higher depth sequencing might be artificial. To test this, we used the RNA-seq data to validate the unique AS events presented in each isoform and found that about 2% of isoforms were not supported by

RNA-seq in regard to special AS events. These may have been either produced artificially or their expression was too low to be covered by RNA-seq. We have mentioned this in the text (Line 240-248).

- Lines 243-245: The transcripts with the most isoforms are enriched for very broad GO terms, is there a biological meaning?
Response: The reviewer is correct that the transcripts with the most isoforms participated in very broad biological processes. We highlighted some GO terms of potential biological interest in the revision. These include many GO terms associated with cell signal transduction, including signal transduction (GO:0007165), cell communication (GO:0007154), signaling (GO:0023052), cation channel activity (GO:0005261), ion channel activity (GO:0005216), and potassium channel activity (GO:0005267). Increasing the abundance of transcripts for these genes might enhance cellular responses to environmental stimuli. Detailed GO terms have been added in Supplementary Table S13. We also changed the sentence accordingly (Line 264-269).

- Lines 250: The authors use the acronym AS in the passages before, they should move the explanation of the acronym to the first occurrence of alternative splicing in the text.
Response: Thank you. This has been corrected.

- Lines 272-274: Why was not an alternative splicing analysis performed, e.g. using DEXSeq?
Response: The DEXSeq package is designed to detect differential exon usage for short-read RNA-seq data. The ISO-seq data have a completely different output as the RNA-seq data. The isoforms produced by ISO-seq already provided the direct usage information of the exon usage. The identification of consensus isoforms and collapsing all isoforms to produce unique isoforms are two key steps in handling the ISO-seq data. The method (alternative_splice.py, https://github.com/Nextomics/pipeline-for-isoseq) used in this analysis was specifically designed to determine alternative splicing using ISO-seq data. It is robust and has been used in many ISO-seq studies, such as Wang et al, 2018; Zhang et al, 2019; Ren et al, 2020. This has been explained in the Methods section (Line 607-612).
Wang M, Wang P, Liang F, Ye Z, Li J, Shen C, et al. A global survey of alternative splicing in allopolyploid cotton: landscape, complexity and regulation. New phytologist. 2018; 217:163–78.
Zhang Y, Dong W, Zhao X, Song A, Guo K, Liu Z, Zhang L. Transcriptomic Analysis of differentially expressed genes and alternative splicing events associated with crassulacean acid metabolism in orchids. Horticultural Plant Journal. 2019; 5(6):268-80.
Ren L, Yan X, Gao X, Cui J, Yan P, Wu C, Li W, Liu S. Maternal effects shape the alternative splicing of parental alleles in reciprocal cross hybrids of Megalobrama amblycephala × Culter alburnus. BMC genomics. 2020; 21(1):457.

- Lines 276-285: Only one of 267 candidate genes is discussed. Maybe a GO enrichment analysis at this point could shed light on the functionality of these candidate genes.
Response: Thank you for your suggestions. We performed the analysis and highlighted some potentially interesting GO terms enriched in this dataset (Line 296-299). Details are provided in Supplementary Table S19.

- Lines 292-295: This sentence is hard to read and to understand. Please try to rephrase it in an understandable manner.
Response: We apologize for this error. We changed the sentence to “Here, we detected 1 225 long transcripts that likely function as lncRNAs based on their lack of open reading frames (See Methods)”.

- Lines 325-328: Elaborate on odorant-binding proteins and their role in social interactions.
Response: We have revised this part. Because both the lncRNA and upstream gene showed significant worker-biased expression, we highlighted its upstream gene retn instead of Obp69a (Line 348-352).

- Lines 329 and following: This is more a conclusion than a discussion. It would be useful if the text would have a clearer structure.
Response: Thank you. We changed the subheading to ‘Conclusions’.

Analyses & Methods:
- Lines 106-107: Why were not multiple kmer sizes evaluated?
Response: For most eukaryotic genomes, 17-mer is the routine kmer frequency distribution analysis (Marçais & Kingsford, 2011). To confirm the validation of genome size, we also selected multiple k-mer sizes, i.e., 17, 19, 21, and 23, to estimate the genome size. Results of different k-mer sizes were similar, and the genome size was estimated to be ~350 Mb. As the pharaoh ant genome was small, 17-mer analysis was sufficient to cover all fragments of the genome. Thus, we selected the 17-mer analysis

results in the manuscript. We also changed the wording as follows:
Following routine 17-mer analysis [26] with short-read sequencing, the genome of M. pharaonis was estimated to be 342 Mb (Supplementary Fig. S1, Table S1). Using other K-mer sizes produced similar estimations.
Marçais, G. & Kingsford, C. A fast, lock-free approach for efficient parallel counting of occurrences of k-mers. Bioinformatics. 2011; 27, 764–770.

- Lines: 146-148: How was this number of genes predicted?
Response: Firstly, we combined homology-, de novo-, and transcriptome-RNA-seq-based gene prediction methods to predict the protein-coding sequences in the pharaoh ant genome. We then used the ISO-seq isoforms to improve the gene models predicted from the previous step. Briefly, we compared the ISO-seq transcripts with the RNA-seq-based predictions, modified the UTRs, added additional coding exons, modified incorrect gene models, and added new gene models. Finally, a total of 15 327 non-redundant protein-coding genes were predicted in the pharaoh ant genome assembly. This has been modified for clarify in the main text (Line 152-158) and method (Line 517-548).

- Lines 174-203: The authors describe in the text that they annotated the genome both using short- and long-reads but separately. For the final annotation, were these annotations merged or was just the long-read annotation taken?
Response: We apologize for the confusion. As stated in the previous question, the final annotations used were the merged ISO-seq and RNA-seq annotations. We have clarified this in the text (Line 216-217).

- Line 352: Whole-body samples of adult ants were used for sequencing. Did the authors check for contamination of the samples for example by gut bacteria?
Response: Thank you for the suggestion. We investigated potential genome assembly contamination by aligning the genome sequences against the Bacteria and Virus databases using BLAST (-e 1e-5), respectively. Total length of contaminated sequences was 2 424 757 bp, which accounted for 0.75% of the genome sequences. The most frequently aligned bacteria were endosymbionts of insects, such as Wolbachia, Bacillus, Candidatus, and Acinetobacter. We filtered the contaminated contigs with contaminated sequences >=20%, which was 151 589 bp. We did not find virus contamination in the genome sequences.

- Lines 421-431: As the authors used female samples for DNA-Seq, the samples were diploid. Did they try to resolve haplotypes using specialized software?
Response: Thank you for your suggestion. Because the PacBio long-read technology requires a large amount (> 5 μg) of high molecular weight DNA to build the library, we pooled many individual ants (> 100) to satisfy this requirement. As many individual genomes were pooled for sequencing, the final assembly produced was a mixed genome from many individuals. Therefore, we did not perform haplotype phasing in the assembly.

- Lines 447-448: Could the authors please elaborate on the choice of non-default parameters and their function here?
Response: The parameters used in the analysis were: “freebayes -C 2 -0 -O -z 0.10 -E 0 - X -u -F 0.6”, as referenced from Jain et al. (2018). We selected non-default parameters to exclude alignment errors. More stringent values were used than the default parameters. We have cited this reference in the revised Methods section. To clarify, the functions of the non-default parameters were as follows:
-C 2 (--min-alternate-count). At least two counts of observations supporting an alternate allele within a single individual are required to evaluate the position. The default value was 1. We changed the value to acquire the confident position.
-0 --no-filters. No input base and mapping quality filters were used.
-O --left-align-indels. Left-realign and merge gaps embedded in reads
-z --read-max-mismatch-fraction. This excluded reads with more than N [0,1] fraction of mismatches where each mismatch had a base quality >= mismatch-base-quality-threshold. We used a stringent value to exclude mismatches
-E --max-complex-gap. This allows complex alleles with contiguous embedded matches of up to this length. We set the value to 0 to exclude complex alleles.
-X --no-mnps. This ignores multi-nucleotide polymorphisms, MNPs.
-u --no-complex. This ignores complex events (composites of other classes).
-F --min-alternate-fraction. This requires at least 60% of observations to support an alternate allele within a single individual in order to evaluate the position.
Jain M, Koren S, Miga KH, Quick J, Rand AC, Sasani TA, Tyson JR, Beggs AD, Dilthey AT, Fiddes IT, Malla S. Nanopore sequencing and assembly of a human genome with ultra-long reads. Nature biotechnology.

2018; 36(4):338-45.

Tables:
- 1: How many of the BUSCOs were duplicated?
Response: Duplicated BUSCOs accounted for 2.1%. The number has been added to Table 1.

- 4: Maybe add some statistics, for example to support the claim that the queens have the longest lncRNA, is there a way to test this?
Response: We performed a Wilcox test for the lncRNAs between queens and other castes. Results showed significant differences between queens and other castes (Wilcoxon test, $p < 0.01$). We added this in the text.

Marah Stoldt and Romain Libbrecht

[Reviewer #2’s comments]:
Reviewer #2: Comments and suggestions:
1) [Lines 153-162] The authors observed 150 large chromosomal rearrangements relative to another ant with a high quality genome and indicate that this value is high. I think it would be helpful to provide some context. For example, such as with other species pairs and/or regarding divergence (by time or by generation). Perhaps the differences observed is just a normal rate of chromosomal mutations?
Response: Thank you for your suggestion. We calculated the rate of chromosomal rearrangement. The estimated rearrangement rate was about 2.04 chromosome breakages per Mb per MY between the two ant species, which is a much higher evolutionary rate than that in the Drosophila genus, which is about 0.05253-0.08485 chromosome breakages per Mb per MY (Ranz et al., 2001). We have added these analyses in the text accordingly (Line 173-174).
Ranz JM, Casals F, Ruiz A. How malleable is the eukaryotic genome? Extreme rate of chromosomal rearrangement in the genus Drosophila. Genome Research. 2001;11(2):230-9.

2) [Lines 312-316] Regarding the analysis of ant conserved lncRNAs.
As it reads, it feels like the message is that the analysis identified ant specific lncRNAs, but this might not be the case. It would be interesting to determine if these were also conserved outside of ants, so that it can be partitioned to conserved (ancient?) insect lncRNAs versus putative ant specific ones. Perhaps a cursory analysis against the standard insect models may be sufficient.
Response: The reviewer has raised a good point. We further extended this analysis to other insect model species, i.e., parasitoid wasp (Nasonia vitripennis), honeybee (Apis mellifera), and fruit-fly (Drosophila melanogaster). Our analysis discovered 33 lncRNAs were conserved between ants and bee, 12 were conserved between ants and wasp, and only six were conserved between ants and fly. Therefore, the reviewer is correct that most lncRNAs were ant specific. We have included this analysis in the revision (Line 337-343).

Minor:
1) Did the authors examine and exclude potential contamination from other organisms (e.g., Kraken or other) in the dataset? Related, sometimes contigs will have very low read coverage (even 1 in my experience), presumably because it was contamination, but are still part of the canu output. Did the authors examine this possibility and remove very spurious contigs?
Response: Thank you for the suggestions. Kraken is a system for assigning taxonomic labels to short DNA sequences and is not efficient for the PacBio long-reads. Here, we aligned our assembly to the Bacteria and Virus databases to remove potential contaminated sequences. By doing so, we filtered out 151 589 bp of contaminated sequences, which were mostly from endosymbionts in insects, such as Wolbachia, Bacillus, Candidatus, and Acinetobacter. We have mentioned this in the revision (Line 114-117, Line 467-473).

2) Related to #1, did the authors consider using Purge Haplotigs (or similar) to remove potential false duplications, which also occurs at scaffold/contig ends?
Response: Thanks for your suggestion. The potential false duplications were removed using purge_haplotigs. Thus, we filtered out 12 469 513 bp of haplotigs and artefacts. The final pharaoh ant genome assembly from the PacBio reads was 312 903 204 bp. We have clarified this in both the Results (Line 117-118) and Methods (Line 474-480) sections.

3) [Line 128] "…contig N50 of 18.6 Mb…" does not match Table 1 value. By extension, contig N90 in Table 1 probably needs double checking.

Response: Thank you very much for pointing out this error. The contig N50 should be 2.5 Mb in the final assembly. We have corrected this in the text.

3B) Related, it might be worth a quick mention as to why Max contig length decreased with the Hi-C assembly. Is it because SSPACE (or possibly) Canu was over aggressive in scaffolding?
Response: The reviewer is correct that some of the mis-link between the scaffolds might be introduced by SSPACE Canu assembly. These links were split if they were not supported by the Hi-C data or conflicted with Hi-C links, thus the final contig size was a little smaller in the Hi-C assembly.

4) Since the authors used the 3d-dna pipeline, did the authors do any manual curating (assembly review), for example with JuiceBox Assembly Tools? And if not, perhaps the authors could provide a quick explanation of why it was not necessary.
Response: We did not perform manual curation with the JuiceBox tool. However, we manually checked the Hi-C heatmap and did not find any obvious assembly errors, such as translocation or inversion in the Hi-C assembly. Overall, the number of the linkage groups we produced was consistent with the number of karyotypes reported for this species.

5) Given that M. pharaonis can be inbred and the starting strain/colony could become an important reference strain for this species, it might be a good idea to provide the strain/colony name.
Response: Thank you for your suggestion. We have added the starting colony information in the Methods section.

Close
