# Supplementary material for: High-quality chromosome-level genome assembly and full-length transcriptome analysis of the pharaoh ant Monomorium pharaonis
Source: Gigascience. 2020 Dec 15;9(12):giaa143. doi: 10.1093/gigascience/giaa143 (PMC7736795; doi:10.1093/gigascience/giaa143)
Supplement: giaa143_Response_to_Reviewer_Comments_Revision_1 [file giaa143_response_to_reviewer_comments_revision_1.pdf]

Author's Response To Reviewer Comments

Close

Response to the comments of Reviewer #1

This revised version of the manuscript has improved in writing and clarity. In response to our suggestion, the authors have toned down the biological insights of the study. We think that it has benefited the paper, as the methodological aspects remain in our opinion the main strength of this study. The authors took most, if not all our minor technical comments into account in the revision of the manuscript, and we are happy with how the issues were addressed.

Our main concern remains the issue of biological replication. We agree with the authors that ideally biological replication should have been conducted for ISO-seq to produce robust caste-specific analyses, but also appreciate that the additional cost of performing such replication prohibited the authors from doing so. We are happy that the authors toned down the caste-specific results, but we think they should address the issue of replication more directly in the manuscript. This mostly concerns the claims of caste-specific AS patterns based on the ISO-seq data (lines 270-307). We propose two ways to do so.

Option 1. The authors clearly acknowledge alternative explanations in the manuscript. The absence of biological replication for ISO-seq means that caste-specific patterns could be specific to the one source colony they used - and not be representative of the population or the species - and/or to the samples they compared (irrespective of caste). Thus, their findings could be colony-specific and/or sample specific, instead of generalized, caste-specific patterns.

Option 2. The authors could use the available, replicated RNA-seq data to confirm patterns that would be expected if the ISO-seq findings indeed stemmed from caste-specific patterns. They could map and count RNA-seq reads for all isoforms of all genes that showed caste-specific patterns with ISO-seq, and perform statistical tests to confirm it is also the case with the RNA-seq data. For example, the number of RNA-seq reads for the worker-specific isoform of crlf3 (lines 299-307) should be significantly higher in workers compared to other castes. This is merely an example, and in theory, this verification could be done for all cases of caste-specific AS (5359 genes, line 275). We are aware that such analysis may not be possible (because of coverage or mapping issues), but in that case, we would recommend that the authors choose Option 1.
Response：We appreciate the reviewers' suggestions on this. As acknowledged by the reviewer, there is a limitation of using RNA-seq to confirm the different splicing forms. The coverage and mapping issues for the short read sequences inhibit us to use RNA-seq data to confirm the caste-specific patterns for all splicing forms. We agree with the reviewers that other factors might also explain the presence and absence patterns of some of the alternative splicing forms. We have added sentences to discuss this in the revision (see line 277-282).
For lncRNAs, the authors did use the RNA-seq data to quantify and compare expression levels among castes, but it is surprising that they did not use any statistical tests to do so. They simply defined differentially expressed lncRNAs as those showing a >1.5-fold difference between castes. This is not conservative, and it is prone to false positives, as such a difference could be explained by chance and this criteria does not take into account within-caste variation. The authors should run statistical analyses to first identify the lncRNAs for which caste affects the expression level, and then to identify which caste differs from which in post-hoc comparisons. This is critical to determine whether their results could have been expected by chance.

Finally, although replicated (n = 5), the quantification analyses of caste-specific differences combined published data (for queens, gynes and workers) and newly generated data (for males). Thus in these analyses, caste is confounded with many other factors (source colony, time of collection, processing batch, etc), which could be alternative explanations to the caste-specific patterns detected in the analyses (at least when males differ from the rest). As recommended in our review of the first submission, this should be clearly acknowledged in the main text.
Response: We have now updated the different expression level analyses for lncRNA with the worker and gyne brain RNA-seq data, which were produced from the same study (Qiu et al. 2018). We detected the

differently-expressed lncRNAs using DESeq2. Then classified lncRNA transcripts as differentially expressed between castes when false discovery rate (FDR) adjusted P-value was ≤ 0.05. By doing so, we identified 32 conserved lncRNAs showing significantly different expression between worker and gyne brains. We have modified the paragraph accordingly (Line 351-356,670-684).

Although we are critical on the issue of replication and statistical analyses, we want to reiterate that this manuscript is very interesting, timely, and that the amount of data provided alongside this manuscript, as well as the innovative methods used, fit the aims of this journal well. This study clearly demonstrates the importance of long-read sequencing to improve genome quality and gene annotation, and more generally to conduct genomic and transcriptomic studies. Should the authors address our concerns by acknowledging alternative explanations in the manuscript or confirming the ISO-seq findings of caste-specific AS with RNA-seq data, as well as adding appropriate statistical analyses, we would recommend the publication of this manuscript in GigaScience.

Marah Stoldt and Romain Libbrecht

Response: We very appreciate both reviewers' great comments which are helpful to improve our manuscript. We have adopted the reviewers' comments and revised the manuscript accordingly.

Close
