# Supplementary material for: High-quality chromosome-level genome assembly and full-length transcriptome analysis of the pharaoh ant Monomorium pharaonis
Source: Gigascience. 2020 Dec 15;9(12):giaa143. doi: 10.1093/gigascience/giaa143 (PMC7736795; doi:10.1093/gigascience/giaa143)
Supplement: giaa143_Reviewer_1_Report_Original_Submission — Libbrecht -- 6/30/2020 Reviewed [file giaa143_reviewer_1_report_original_submission.pdf]

Reviewer Report

Title: High-quality chromosome-level genome assembly and full-length transcriptome analysis of the pharaoh ant Monomorium pharaonis

Version: Original Submission **Date:** 6/30/2020

Reviewer name: Romain Libbrecht

Reviewer Comments to Author:

The manuscript "A high-quality chromosome-level pharaoh ant genome assembly and full-length transcriptome provide insights on ant caste differentiation" contributes to the field of sociogenomics by providing and reporting on an improved version of the genome of the pharaoh ant Monomorium pharaonis. The amount of data provided alongside this manuscript as well as the innovative methods used, fit the aims of this journal well.

In general, this manuscript adds to the growing number of ant genomes published. To assemble genomes at the chromosome level at high quality is essential for follow-up studies to produce reliable results. Thus, this work is of great interest for studies on M. pharaonis as well as for comparative studies across ant or Hymenopteran species. The authors implemented several steps in their pipeline to ensure a sufficient quality of the genome, still some details are missing and should be reported (see Additional comments).

This study clearly demonstrates the importance of long-read sequencing to improve genome quality and gene annotation, and more generally to conduct genomic and transcriptomic studies. While the technical sections of the study are very strong, the biological aspects are relatively weak. The absence of biological replication in the experimental design and the presence of confounding factors (samples collected in different colonies or comparison across studies with one treatment group in one study, and another treatment group in another study) make it impossible to assess whether the caste-specific patterns reported in this manuscript reflect a biological reality or merely stem from sample-specific noise (that could come from technical and/or biological random variation).

In our opinion, this study would benefit (and its scope would better fit GigaScience) from toning down (or even removing) the attempt at biological interpretation to focus on its very important finding that long-read sequencing may be a game changer in the field of sociogenomics.

Additional comments

- The manuscript is riddled with typos and grammar errors, and would definitely benefit from being corrected by an English native speaker.
- Line 162: The results supporting genomic rearrangements between M. pharaonis and O. biroi are interesting, but it is only very superficially discussed from a biological point of view. This is not necessarily problematic, if the point here is to show that long-read sequencing is required to perform such analyses, but then it should be clearly stated and acknowledged.
- Line 165: Is Global Ant Genomic Consortium GAGA? Should it be Alliance instead of Consortium? Are these reference genomes accessible by everyone? Which versions of these genomes were used? Are they published? If not, they should be made accessible together with the paper according to GigaScience

guidelines.
Results:
- Lines 227-229: The number of isoforms is steadily increased with the coverage. What are the implications? Is there the possibility of false positives?
- Lines 243-245: The transcripts with the most isoforms are enriched for very broad GO terms, is there a biological meaning?
- Lines 250: The authors use the acronym AS in the passages before, they should move the explanation of the acronym to the first occurrence of alternative splicing in the text.
- Lines 272-274: Why was not an alternative splicing analysis performed, e.g. using DEXSeq?
- Lines 276-285: Only one of 276 candidate genes is discussed. Maybe a GO enrichment analysis at this point could shed light on the functionality of these candidate genes.
- Lines 292-295: This sentence is hard to read and to understand. Please try to rephrase it in an understandable manner.
- Lines 325-328: Elaborate on odorant-binding proteins and their role in social interactions.
- Lines 329 and following: This is more a conclusion than a discussion. It would be useful if the text would have a clearer structure.

Analyses & Methods:
- Lines 106-107: Why were not multiple kmer sizes evaluated?
- Lines: 146-148: How was this number of genes predicted?
- Lines 174-203: The authors describe in the text that they annotated the genome both using short- and long-reads but separately. For the final annotation, were these annotations merged or was just the long-read annotation taken?
- Line 352: Whole-body samples of adult ants were used for sequencing. Did the authors check for contamination of the samples for example by gut bacteria?
- Lines 421-431: As the authors used female samples for DNA-Seq, the samples were diploid. Did they try to resolve haplotypes using specialized software?
- Lines 447-448: Could the authors please elaborate on the choice of non-default parameters and their function here?

Tables:
- 1: How many of the BUSCOs were duplicated?
- 4: Maybe add some statistics, for example to support the claim that the queens have the longest lncRNA, is there a way to test this?

Marah Stoldt and Romain Libbrecht

Methods

Are the methods appropriate to the aims of the study, are they well described, and are necessary controls included? Choose an item.

Conclusions

Are the conclusions adequately supported by the data shown? Choose an item.

Reporting Standards

Does the manuscript adhere to the journal's guidelines on minimum standards of reporting? Choose an item.

Choose an item.

Statistics

Are you able to assess all statistics in the manuscript, including the appropriateness of statistical tests used? Choose an item.

Quality of Written English

Please indicate the quality of language in the manuscript: Choose an item.

Declaration of Competing Interests

Please complete a declaration of competing interests, considering the following questions:

- Have you in the past five years received reimbursements, fees, funding, or salary from an organisation that may in any way gain or lose financially from the publication of this manuscript, either now or in the future?
- Do you hold any stocks or shares in an organisation that may in any way gain or lose financially from the publication of this manuscript, either now or in the future?
- Do you hold or are you currently applying for any patents relating to the content of the manuscript?
- Have you received reimbursements, fees, funding, or salary from an organization that holds or has applied for patents relating to the content of the manuscript?
- Do you have any other financial competing interests?
- Do you have any non-financial competing interests in relation to this paper?

If you can answer no to all of the above, write 'I declare that I have no competing interests' below. If your reply is yes to any, please give details below.

We declare that we have no competing interests

I agree to the open peer review policy of the journal. I understand that my name will be included on my report to the authors and, if the manuscript is accepted for publication, my named report including any attachments I upload will be posted on the website along with the authors' responses. I agree for my report to be made available under an Open Access Creative Commons CC-BY license (http://creativecommons.org/licenses/by/4.0/). I understand that any comments which I do not wish to be included in my named report can be included as confidential comments to the editors, which will not be published.

Choose an item.

To further support our reviewers, we have joined with Publons, where you can gain additional credit to further highlight your hard work (see: https://publons.com/journal/530/gigascience). On publication of this paper, your review will be automatically added to Publons, you can then choose whether or not to claim your Publons credit. I understand this statement.

Yes Choose an item.
