# Supplementary material for: High-quality chromosome-level genome assembly and full-length transcriptome analysis of the pharaoh ant Monomorium pharaonis
Source: Gigascience. 2020 Dec 15;9(12):giaa143. doi: 10.1093/gigascience/giaa143 (PMC7736795; doi:10.1093/gigascience/giaa143)
Supplement: giaa143_Reviewer_1_Report_Revision_1 — Libbrecht -- 9/30/2020 Reviewed [file giaa143_reviewer_1_report_revision_1.pdf]

Reviewer Report

Title: High-quality chromosome-level genome assembly and full-length transcriptome analysis of the pharaoh ant Monomorium pharaonis

Version: Revision 1 **Date:** 9/30/2020

Reviewer name: Romain Libbrecht

Reviewer Comments to Author:

This revised version of the manuscript has improved in writing and clarity. In response to our suggestion, the authors have toned down the biological insights of the study. We think that it has benefited the paper, as the methodological aspects remain in our opinion the main strength of this study. The authors took most, if not all our minor technical comments into account in the revision of the manuscript, and we are happy with how the issues were addressed.

Our main concern remains the issue of biological replication. We agree with the authors that ideally biological replication should have been conducted for ISO-seq to produce robust caste-specific analyses, but also appreciate that the additional cost of performing such replication prohibited the authors from doing so. We are happy that the authors toned down the caste-specific results, but we think they should address the issue of replication more directly in the manuscript. This mostly concerns the claims of caste-specific AS patterns based on the ISO-seq data (lines 270-307). We propose two ways to do so.

Option 1. The authors clearly acknowledge alternative explanations in the manuscript. The absence of biological replication for ISO-seq means that caste-specific patterns could be specific to the one source colony they used - and not be representative of the population or the species - and/or to the samples they compared (irrespective of caste). Thus, their findings could be colony-specific and/or sample specific, instead of generalized, caste-specific patterns.

Option 2. The authors could use the available, replicated RNA-seq data to confirm patterns that would be expected if the ISO-seq findings indeed stemmed from caste-specific patterns. They could map and count RNA-seq reads for all isoforms of all genes that showed caste-specific patterns with ISO-seq, and perform statistical tests to confirm it is also the case with the RNA-seq data. For example, the number of RNA-seq reads for the worker-specific isoform of crlf3 (lines 299-307) should be significantly higher in workers compared to other castes. This is merely an example, and in theory, this verification could be done for all cases of caste-specific AS (5359 genes, line 275). We are aware that such analysis may not be possible (because of coverage or mapping issues), but in that case, we would recommend that the authors choose Option 1.

For lncRNAs, the authors did use the RNA-seq data to quantify and compare expression levels among castes, but it is surprising that they did not use any statistical tests to do so. They simply defined differentially expressed lncRNAs as those showing a >1.5-fold difference between castes. This is not conservative, and it is prone to false positives, as such a difference could be explained by chance and this criteria does not take into account within-caste variation. The authors should run statistical analyses to first identify the lncRNAs for which caste affects the expression level, and then to identify which caste differs from which in post-hoc comparisons. This is critical to determine whether their results could have

been expected by chance.
Finally, although replicated (n = 5), the quantification analyses of caste-specific differences combined published data (for queens, gynes and workers) and newly generated data (for males). Thus in these analyses, caste is confounded with many other factors (source colony, time of collection, processing batch, etc), which could be alternative explanations to the caste-specific patterns detected in the analyses (at least when males differ from the rest). As recommended in our review of the first submission, this should be clearly acknowledged in the main text.
Although we are critical on the issue of replication and statistical analyses, we want to reiterate that this manuscript is very interesting, timely, and that the amount of data provided alongside this manuscript, as well as the innovative methods used, fit the aims of this journal well. This study clearly demonstrates the importance of long-read sequencing to improve genome quality and gene annotation, and more generally to conduct genomic and transcriptomic studies. Should the authors address our concerns by acknowledging alternative explanations in the manuscript or confirming the ISO-seq findings of caste-specific AS with RNA-seq data, as well as adding appropriate statistical analyses, we would recommend the publication of this manuscript in GigaScience.
Marah Stoldt and Romain Libbrecht

Methods

Are the methods appropriate to the aims of the study, are they well described, and are necessary controls included? Choose an item.

Conclusions

Are the conclusions adequately supported by the data shown? Choose an item.

Reporting Standards

Does the manuscript adhere to the journal's guidelines on [minimum standards of reporting?] Choose an item.

Choose an item.

Statistics

Are you able to assess all statistics in the manuscript, including the appropriateness of statistical tests used? Choose an item.

Quality of Written English

Please indicate the quality of language in the manuscript: Choose an item.

Declaration of Competing Interests

Please complete a declaration of competing interests, considering the following questions:

- Have you in the past five years received reimbursements, fees, funding, or salary from an organisation that may in any way gain or lose financially from the publication of this manuscript, either now or in the future?
- Do you hold any stocks or shares in an organisation that may in any way gain or lose financially from the publication of this manuscript, either now or in the future?
- Do you hold or are you currently applying for any patents relating to the content of the manuscript?
- Have you received reimbursements, fees, funding, or salary from an organization that holds or has applied for patents relating to the content of the manuscript?
- Do you have any other financial competing interests?
- Do you have any non-financial competing interests in relation to this paper?

If you can answer no to all of the above, write 'I declare that I have no competing interests' below. If your reply is yes to any, please give details below.

I declare that I have no competing interests

I agree to the open peer review policy of the journal. I understand that my name will be included on my report to the authors and, if the manuscript is accepted for publication, my named report including any attachments I upload will be posted on the website along with the authors' responses. I agree for my report to be made available under an Open Access Creative Commons CC-BY license (http://creativecommons.org/licenses/by/4.0/). I understand that any comments which I do not wish to be included in my named report can be included as confidential comments to the editors, which will not be published.

Choose an item.

To further support our reviewers, we have joined with Publons, where you can gain additional credit to further highlight your hard work (see: https://publons.com/journal/530/gigascience). On publication of this paper, your review will be automatically added to Publons, you can then choose whether or not to claim your Publons credit. I understand this statement.

Yes Choose an item.
