# Supplementary material for: High-quality chromosome-level genome assembly and full-length transcriptome analysis of the pharaoh ant Monomorium pharaonis
Source: Gigascience. 2020 Dec 15;9(12):giaa143. doi: 10.1093/gigascience/giaa143 (PMC7736795; doi:10.1093/gigascience/giaa143)
Supplement: giaa143_Reviewer_2_Report_Original_Submission — John Wang -- 7/1/2020 Reviewed [file giaa143_reviewer_2_report_original_submission.pdf]

**Reviewer Report**

**Title: High-quality chromosome-level genome assembly and full-length transcriptome analysis of the pharaoh ant Monomorium pharaonis**

**Version: Original Submission** **Date:** 7/1/2020

**Reviewer name: John Wang**

**Reviewer Comments to Author:**

The authors have generated a chromosome level assembly reference genome for the pharaoh ant, Monomorium pharaonis, which is much better than a previously published version using short read technology. They did this using a combination of Illumina, PacBio and Hi-C technologies. They have also improved annotation significantly by including new technologies, notably PacBio RNA sequencing (ISO-seq).
Notable points in this study are:
Much better annotation: partly as a benefit of a better assembly and more importantly from PacBio ISO-seq annotation.
Using the ISO-seq data allowed the authors to conduct an extensive analysis of alternative splicing, including by caste (and sex).
Identification and comparative analysis of long noncoding RNAs (lncRNAs)
The authors also provide some interesting analysis vignettes regarding social insect specific biology, such as the sex determination genes (fem and csd) and finding caste specific lncRNAs.
Overall this study provides an important foundation for ant and insect genomics.
Comments and suggestions:
1) [Lines 153-162] The authors observed 150 large chromosomal rearrangements relative to another ant with a high quality genome and indicate that this value is high. I think it would be helpful to provide some context. For example, such as with other species pairs and/or regarding divergence (by time or by generation). Perhaps the differences observed is just a normal rate of chromosomal mutations?
2) [Lines 312-316] Regarding the analysis of ant conserved lncRNAs.
As it reads, it feels like the message is that the analysis identified ant specific lncRNAs, but this might not be the case. It would be interesting to determine if these were also conserved outside of ants, so that it can be partitioned to conserved (ancient?) insect lncRNAs versus putative ant specific ones. Perhaps a cursory analysis against the standard insect models may be sufficient.
Minor:
1) Did the authors examine and exclude potential contamination from other organisms (e.g., Kraken or other) in the dataset? Related, sometimes contigs will have very low read coverage (even 1 in my experience), presumably because it was contamination, but are still part of the canu output. Did the authors examine this possibility and remove very spurious contigs?
2) Related to #1, did the authors consider using Purge Haplotigs (or similar) to remove potential false duplications, which also occurs at scaffold/contig ends?
3) [Line 128] "…contig N50 of 18.6 Mb…" does not match Table 1 value. By extension, contig N90 in

Table 1 probably needs double checking.
3B) Related, it might be worth a quick mention as to why Max contig length decreased with the HiC assembly. Is it because SSPACE (or possibly) Canu was over aggressive in scaffolding?
4) Since the authors used the 3d-dna pipeline, did the authors do any manual curating (assembly review), for example with JuiceBox Assembly Tools? And if not, perhaps the authors could provide a quick explanation of why it was not necessary.
5) Given that M. pharaonis can be inbred and the starting strain/colony could become an important reference strain for this species, it might be a good idea to provide the strain/colony name.

**Methods**

Are the methods appropriate to the aims of the study, are they well described, and are necessary controls included? Choose an item.

**Conclusions**

Are the conclusions adequately supported by the data shown? Choose an item.

**Reporting Standards**

Does the manuscript adhere to the journal's guidelines on minimum standards of reporting? Choose an item.

Choose an item.

**Statistics**

Are you able to assess all statistics in the manuscript, including the appropriateness of statistical tests used? Choose an item.

**Quality of Written English**

Please indicate the quality of language in the manuscript: Choose an item.

**Declaration of Competing Interests**

Please complete a declaration of competing interests, considering the following questions:

- Have you in the past five years received reimbursements, fees, funding, or salary from an organisation that may in any way gain or lose financially from the publication of this manuscript, either now or in the future?
- Do you hold any stocks or shares in an organisation that may in any way gain or lose financially from the publication of this manuscript, either now or in the future?
- Do you hold or are you currently applying for any patents relating to the content of the manuscript?

- Have you received reimbursements, fees, funding, or salary from an organization that holds or has applied for patents relating to the content of the manuscript?
- Do you have any other financial competing interests?
- Do you have any non-financial competing interests in relation to this paper?

If you can answer no to all of the above, write 'I declare that I have no competing interests' below. If your reply is yes to any, please give details below.

I declare that I have no competing interests.

I agree to the open peer review policy of the journal. I understand that my name will be included on my report to the authors and, if the manuscript is accepted for publication, my named report including any attachments I upload will be posted on the website along with the authors' responses. I agree for my report to be made available under an Open Access Creative Commons CC-BY license (http://creativecommons.org/licenses/by/4.0/). I understand that any comments which I do not wish to be included in my named report can be included as confidential comments to the editors, which will not be published.

Choose an item.

To further support our reviewers, we have joined with Publons, where you can gain additional credit to further highlight your hard work (see: https://publons.com/journal/530/gigascience). On publication of this paper, your review will be automatically added to Publons, you can then choose whether or not to claim your Publons credit. I understand this statement.

Yes Choose an item.
